# CRISPR/Cas9 and TALENs generate heritable mutations for genes involved in small RNA processing of *Glycine max* and *Medicago truncatula*

**DOI:** 10.1111/pbi.12857

**Published:** 2017-12-04

**Authors:** Shaun J. Curtin, Yer Xiong, Jean‐Michel Michno, Benjamin W. Campbell, Adrian O. Stec, Tomas Čermák, Colby Starker, Daniel F. Voytas, Andrew L. Eamens, Robert M. Stupar

**Affiliations:** ^1^ Department of Plant Pathology University of Minnesota St. Paul MN USA; ^2^ Department of Agronomy and Plant Genetics University of Minnesota St. Paul MN USA; ^3^ Bioinformatics and Computational Biology Graduate Program University of Minnesota Minneapolis MN USA; ^4^ Department of Genetics, Cell Biology & Development Center for Genome Engineering University of Minnesota Minneapolis MN USA; ^5^ School of Environmental and Life Sciences The University of Newcastle Callaghan New South Wales Australia; ^6^ Present address: Plant Science Research Unit Agricultural Research Service United States Department of Agriculture St Paul MN USA; ^7^ Present address: Agricultural Research Service Inari Agriculture, Inc. Cambridge MA USA

**Keywords:** CRISPR/Cas9, mutagenesis, soya bean, Medicago, small RNA, *Drb2*

## Abstract

Processing of double‐stranded RNA precursors into small RNAs is an essential regulator of gene expression in plant development and stress response. Small RNA processing requires the combined activity of a functionally diverse group of molecular components. However, in most of the plant species, there are insufficient mutant resources to functionally characterize each encoding gene. Here, mutations in loci encoding protein machinery involved in small RNA processing in soya bean and *Medicago truncatula* were generated using the CRISPR/Cas9 and TAL‐effector nuclease (TALEN) mutagenesis platforms. An efficient CRISPR/Cas9 reagent was used to create a bi‐allelic double mutant for the two soya bean paralogous *Double‐stranded RNA‐binding2* (*GmDrb*2*a* and *GmDrb2b*) genes. These mutations, along with a CRISPR/Cas9‐generated mutation of the *M. truncatula Hua enhancer1* (*MtHen1*) gene, were determined to be germ‐line transmissible. Furthermore, TALENs were used to generate a mutation within the soya bean *Dicer‐like2* gene. CRISPR/Cas9 mutagenesis of the soya bean *Dicer‐like3* gene and the *GmHen1a* gene was observed in the T_0_ generation, but these mutations failed to transmit to the T_1_ generation. The irregular transmission of induced mutations and the corresponding transgenes was investigated by whole‐genome sequencing to reveal a spectrum of non‐germ‐line‐targeted mutations and multiple transgene insertion events. Finally, a suite of combinatorial mutant plants were generated by combining the previously reported *Gmdcl1a*,* Gmdcl1b* and *Gmdcl4b* mutants with the *Gmdrb2ab* double mutant. Altogether, this study demonstrates the synergistic use of different genome engineering platforms to generate a collection of useful mutant plant lines for future study of small RNA processing in legume crops.

## Introduction

Genome engineering technologies encompass the construction and introduction of site‐specific nucleases (SSNs) into host genomes to create double‐stranded breaks (DSB) at targeted loci. The repair of the DSBs by the error‐prone nonhomologous end‐joining (NHEJ) pathway of the host organism often leads to nucleotide insertion and/or deletion (indels) events that can result in gene disruption. By taking advantage of this endogenous repair mechanism, plant biologists can generate modifications at targeted loci ranging in size from a single to several hundreds of base pairs in length, and in some cases much larger megabase pair deletions (Cermak *et al*., 2017; Qi *et al*., 2013; Voytas, 2013).

Site‐specific nucleases are synthetic enzymes with programmable DNA target specificities and include the (i) zinc‐finger nuclease (ZFN; Kim *et al*., 1996; Sander *et al*., 2011), (ii) transcription activator–like effector nuclease (TALEN; Christian *et al*., 2010; Miller *et al*., 2011) and (iii) clustered, regularly interspaced, short palindromic repeat (CRISPR)‐associated protein (Cas9) (CRISPR/Cas9) system (Cong *et al*., 2013; Mali *et al*., 2013). ZFN and TALEN‐mediated site‐directed mutagenesis has been reported in several plant species including maize (*Zea mays*), tobacco (*Nicotiana tabacum*), barley (*Hordeum vulgare*), soya bean (*Glycine max*) and Arabidopsis (*Arabidopsis thaliana*) (Cermak *et al*., 2011; Curtin *et al*., 2011; Li *et al*., 2012; Mahfouz *et al*., 2011; Wendt *et al*., 2013). CRISPR/Cas9 is the most recent platform and is comprised of the endonuclease (Cas9) and a sequence determining single‐stranded spacer guide RNA (gRNA; Bhaya *et al*., 2011). When introduced to plant cells via *Agrobacterium*‐mediated transformation, the random genome integration of the transfer DNA (T‐DNA) leads to expression of both the Cas9 endonuclease and the spacer gRNA, which when combined, generate a site‐specific DSB. Following transformation, the regenerated population of plants, the T_0_ generation, can be screened directly for lines harbouring mutations at the targeted locus and the heritable transmission of the introduced mutation(s) confirmed in the subsequent T_1_ generation of plants. Once a mutation has been heritably transmitted, it is considered stable and can be readily and reliably transmitted to subsequent generations.

Multiple reports have described the generation of somatic mutations using CRISPR/Cas9 in soya bean (Cai *et al*., 2015; Du *et al*., 2016; Jacobs *et al*., 2015; Lowder *et al*., 2015; Michno *et al*., 2015; Tang *et al*., 2016). To date however, few studies have demonstrated whole‐plant soya bean mutagenesis (Cai *et al*., 2017; Li *et al*., 2015). Here, we describe a set of plant expression vectors that can accommodate either the CRISPR/Cas9 or TALEN entry vectors to target the genes encoding the machinery proteins involved in small RNA processing in soya bean and *M. truncatula* (Baltes *et al*., 2014; Curtin *et al*., 2017; Zhang *et al*., 2013). Small RNAs are central regulators of gene expression associated with legume reproductive development, symbiotic nitrogen fixation, pathogen defence and the response to either biotic or abiotic stress (Arikit *et al*., 2014; Bustos‐Sanmamed *et al*., 2013; Li *et al*., 2010; Yan *et al*., 2015; Zhai *et al*., 2011). We have previously demonstrated that the use of site‐specific nucleases to generate mutations in the genes encoding the core machinery proteins of RNA silencing pathways is a highly successful tool for the study of small RNA biology in nonmodel plant species (Curtin *et al*., 2016). The newly generated mutant lines reported here greatly advance such resources and pave the way for future advances in legume small RNA biology research.

## Results

### Assessment of CRISPR/Cas9 functionality in soya bean hairy‐root tissue and TAL‐effector nuclease activity in yeast

**Figure 1:**
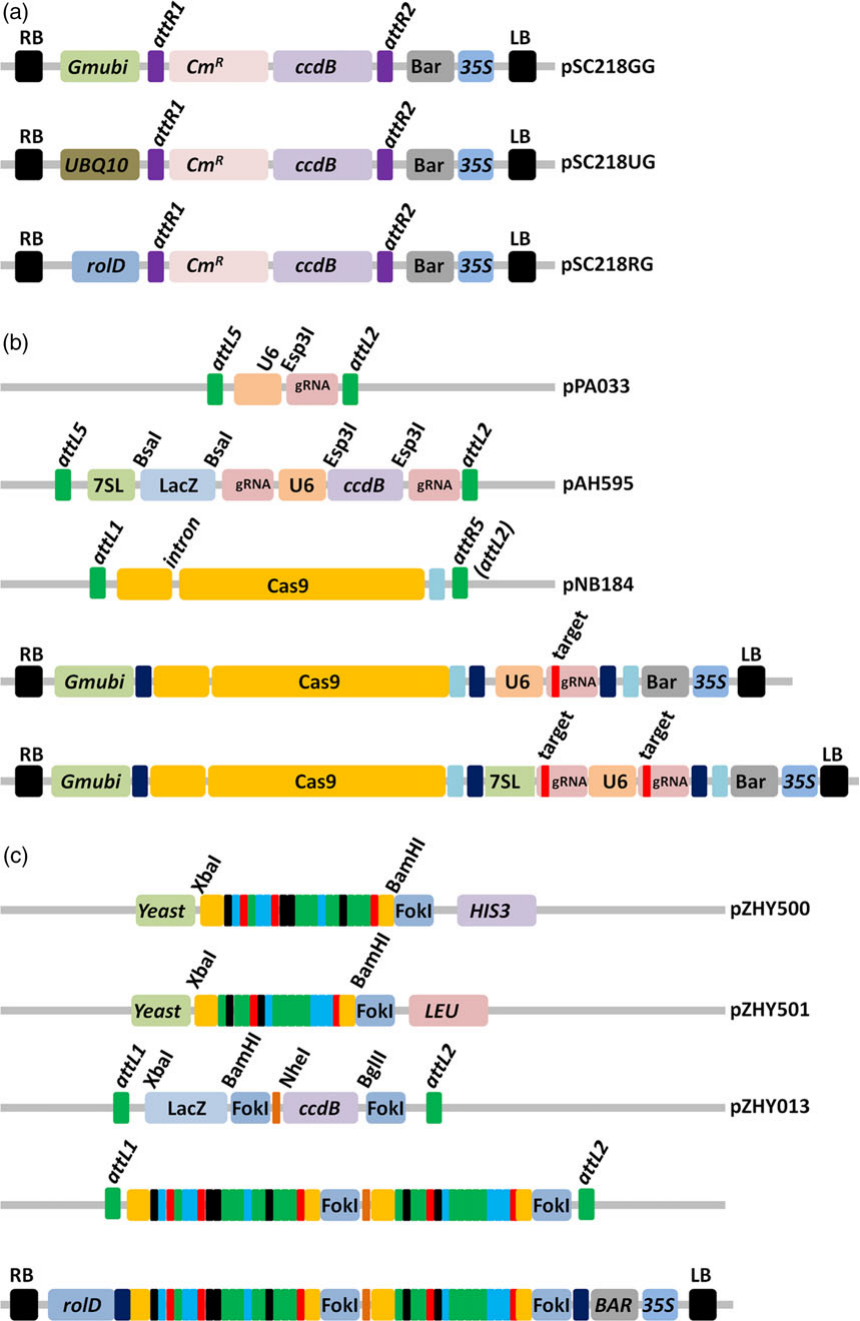
Constructs used in this study. (a) The plant expression vectors used to generate site‐specific mutations in whole‐plant soya bean: the *Glycine max* ubiquitin (*Gmubi*) promoter (pSC218GG), the *Arabidopsis* ubiquitin10 promoter (pSC218UG) and the *Agrobacterium rhizogenes rol*D promoter (pSC218RG). (b) The Cas9 and guide RNA entry vectors used for multisite Gateway™ assembly into the destination plant expression vectors. Target1 and Target2 gRNAs are cloned into the BsaI and Esp3I sites, respectively, using a golden gate ligation reaction. The ccdB and LacZ selection cassettes assist colony screening efficiency of the ligation reaction. The schematic representation of assembled reagents, a single (second from bottom) and a 2x‐plex (bottom) gRNA combined with a *Gmubi‐*expressed Cas9 cassette. (c) Schematic representations of the TAL‐effector assembly reagents. Target RVDs (shown as red, black, green and blue coloured rectangles) were assembled using golden gate ligation reaction into the yeast expression vectors, pZHY500 and pZHY501, for the assessment of their cleavage activity (Figure S3). Selected arrays were sequentially cloned using the depicted restriction enzymes into the pZHY013 entry vector and assembled into the plant expression vector pSC218RG by a Gateway™ reaction.

Three binary plant expression vectors, each with a unique promoter, were converted into Gateway™ destination vectors. The promoters were selected for their constitutive and/or germ‐line expression and included the *Glycine max* ubiquitin (*Gmubi*), *Arabidopsis* ubiquitin10 (UBQ10) and *Agrobacterium rhizogenes Root‐loci*D (*rol*D) gene promoters (Figure 1a). To assess the functionality of each vector in soya bean, we used a previously reported set of CRISPR/Cas9 entry vectors that allow for the incorporation of the Cas9, along with a single (1x‐plex), or double 2x‐plex gRNA cassette, via a multiplex LR clonase reaction (Life Technologies, CA) (Baltes *et al*., 2014; Curtin *et al*., 2017; Figure 1b). We next constructed two single gRNAs entry cassettes that targeted the soya bean *GmDcl3a* gene (Glyma.04g057400; herein referred to as *GmDcl3a*) and carried out the cloning reaction to combine the Cas9 entry cassettes into each destination vector (Figure S1a). All six combinations of the CRISPR/Cas9 plant expression vectors were next transformed into *A. rhizogenes* strain K599 for the generation of transgenic plant tissue using a soya bean hairy‐root *ex vitro* transformation assay (Figure S2a; Taylor *et al*., 2006). DNA extracted from 2‐week‐old hairy‐root tissue was enriched for the mutated sequence by a restriction enzyme assay to remove nonmutated DNA sequences, and this preparation was subsequently used as PCR template (Figure S2b). A *t*arget *a*mplicon *c*lone *a*nd *s*equence (TACAS) assay confirmed deletions at both *GmDcl3a* loci (*GmDcl3a‐1* and *GmDcl3a‐2*) from all three assessed expression vectors (Figure S2c).

To test whether the three assessed plant expression vectors could also be used in conjunction with a previously reported TALEN platform, we selected three soya bean genes to be targeted for TALEN‐directed mutagenesis, namely *GmDcl2a* (Glyma.09g025400), *GmDcl2b* (Glyma.09g025300) and *GmDcl3a* (Glyma.04g057400). Furthermore, the TAL‐effector Nucleotide Targeter 2.0 (TALE‐NT) software was used to identify suitable repeat variable domains (RVD) array configurations (Doyle *et al*., 2012; Zhang *et al*., 2013), and the configured arrays were rapidly assembled using the Golden Gate TAL‐effector nuclease (TALEN) kit. The assembled arrays were next tested for their respective cleavage activities using a yeast‐based assay (Figure 1c; Method S1) (Cermak *et al*., 2011; Christian *et al*., 2010), and TAL arrays determined to have a significant activity were incorporated into the plant expression vectors (Figure S3a–b).

### Successful mutagenesis and mutation transmission of the soya bean *Drb2ab* homoeologues

**Figure 2:**
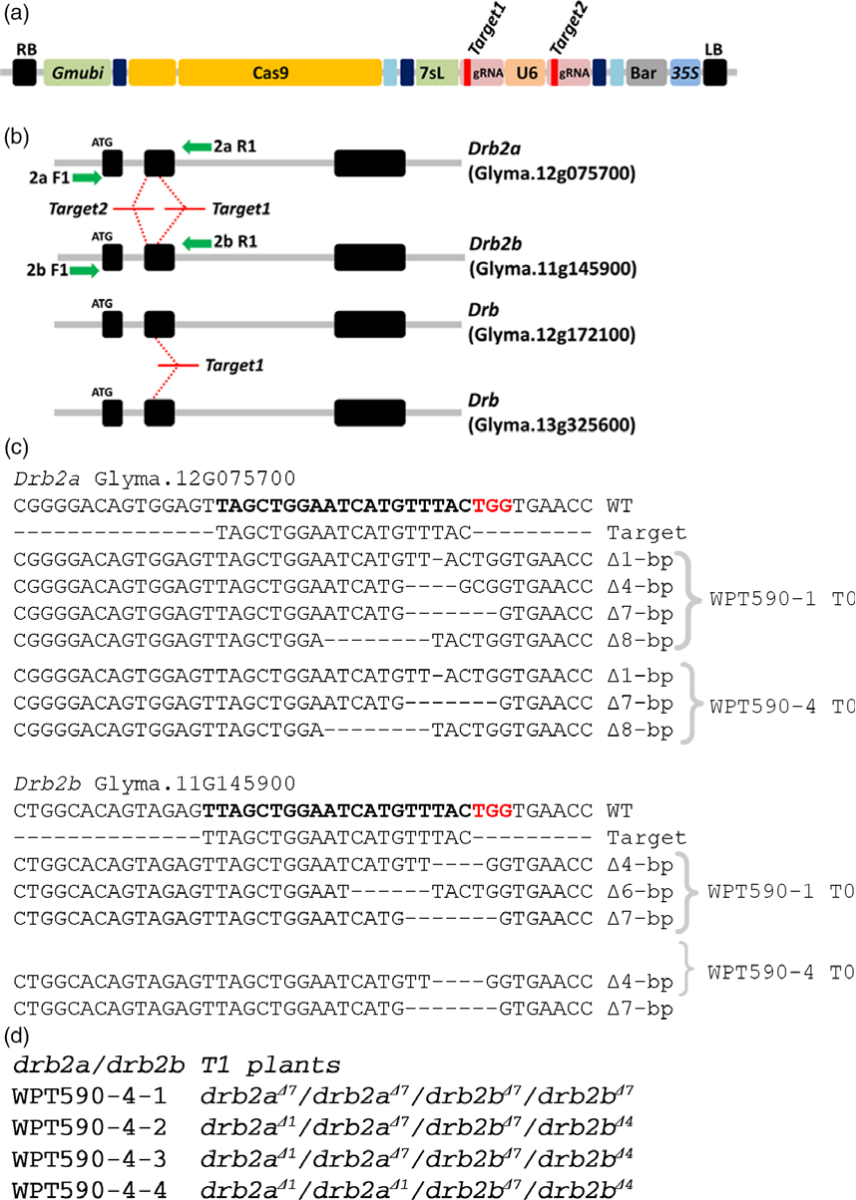
CRISPR/Cas9 mutagenesis and inheritance of mutations for the soya bean paralogous gene pair *GmDrb2a* and *GmDrb2b*. (a) Schematic representation of the CRISPR/Cas9 reagent transgene. (b) The genomic arrangement of *GmDrb2a* and *GmDrb2b*. The ‘*Target1*’ and ‘*Target2*’ represent gRNAs target sites, and the green arrows represent primer sites used to screen mutations (Table S5). (c) The multiple mutated alleles were observed in the WPT590‐1 and WPT590‐4 T_0_ plants from the both the TACAS and WGS analyses at the *Drb2a* and *Drb2b* loci. The target sequences are indicated in boldface, along with the PAM site (red). The ‘*Target 1*’ gRNA also targets two additional (although uncharacterized) *Drb* genes; however, no activity was observed at these sites. (d) The heritable transmission of mutated alleles was confirmed in WPT590‐1 and WPT590‐4 T_1_ plants. The four T_1_ progeny of the WPT590‐4 T_0_ plant shown here are examples of segregation of the Δ7‐bp and Δ1‐bp alleles for *Gmdrb2a* and the Δ7‐bp and Δ4‐bp alleles for *Gmdrb2b*.

A *Gmubi* promoter‐driven CRISPR/Cas9 reagent with two gRNAs targeting sites within both soya bean *Drb2* paralog copies was constructed (*Drb2a*, Glyma.12g075700, and *Drb2b*, Glyma.11g145900; referred to as *GmDrb2a* and *GmDrb2b* herein) (Figure 2a). The U6‐driven gRNA targeted *GmDrb2a* and *GmDrb2b* (‘*Target2*’), while the 7sL‐driven gRNA was designed to target four *Drb* genes, namely *GmDrb2a* and *GmDrb2b* and two additional *Drb* encoding loci (Glyma.12g172100 and Glyma.13g325600) (‘*Target1*’) (Figure 2b). As the 7sL promoter performs poorly in soya bean and *M. truncatula*, we did not expect to detect any activity associated with the 7sL‐driven gRNAs. However, the resulting reagent transgene was still introduced into soya bean via *Agrobacterium*‐mediated transformation and four T_0_ plants, WPT590‐1, WPT590‐2, WPT590‐3, and WPT590‐4, were recovered from this transformation experiment (Figure S4a–d). A PCR digestion assay was used to screen for introduced mutation and this approach showed that three of the four T_0_ plants returned digestion‐resistant amplicons (plants, WPT590‐1, WPT590‐2 and WPT590‐4) (Figure S4a). A heteroduplex and TACAS assay were applied to confirm the mutation status of these three T_0_ plant lines with the two T_0_ plants, WPT590‐1 and WPT590‐4, determined to harbour fully mutated alleles for both the *GmDrb2a* and *GmDrb2b* loci (Figures 2c and S4–S5), while mutated and wild‐type amplicon sequences were isolated from T_0_ plant, WPT590‐2 (Figures S4–S5). Sequence data generated from the TACAS assay also detected the presence of extra mutant amplicons sizes from the individual plants assessed. For example, three mutated *GmDrb2a* alleles (∆8‐bp, ∆7‐bp and a ∆1‐bp) were observed at high frequency across multiple leaf samples (Figures 2c and S5).

To confirm heritable transmission of the introduced mutations, T_0_ plants were self‐fertilized and progeny screened (T_1_ plants) using the heteroduplex and TACAS approach. This approach revealed that the targeted mutations in the WPT590‐1 and WPT590‐4 T_0_ plants had successfully transmitted to the T_1_ generation (Figure S4b,d). However, this analysis also showed that no mutation or reagent transgene was transmitted to the T_1_ progeny of the WPT590‐2 plant (Figures S4c and S5). Whole‐genome sequencing (WGS) on the genomic DNA extracted from T_0_ plants, WPT590‐1 and WPT590‐4, and on two T_1_ progeny of each T_0_ plant (including T1 plants WPT590‐1‐1, WPT590‐1‐2, WPT590‐4‐1, and WPT590‐4‐2, respectively) was used to investigate the targeted loci in greater detail. The WGS approach confirmed the previously identified mutant alleles of a ∆7‐bp and ∆1‐bp for *GmDrb2a* and a ∆7‐bp and ∆4‐bp for *GmDrb2b* in both the T_0_ and T_1_ generations of the WPT590‐1 and WPT590‐4 backgrounds (Table S1; Figures S6 and S7). Interestingly, the four transmitted mutant alleles were identical between the T_1_ progeny derived from T_0_ plants WPT590‐1 and WPT590‐4. This finding indicates that the two T_0_ plants may have been independent regenerants of the same transformation and mutagenesis event. Furthermore, TACAS assay assessment of WPT590‐4 T_1_ progeny indicated that the four identified mutant alleles were segregating in the T_1_ generation (Figure 2d).

Upon successful heritable transmission of each mutant allele, additional somatic mutations in T_1_ plants are not expected to be detected due to the removal of the target sites by the bi‐allelic mutation. This was determined to be the case in these analyses, as no new mutations were identified at the targeted sites of either the *GmDrb2a* or *GmDrb2b* locus. A single wild‐type read was detected for the WPT590‐1‐1 sample; however, it is assumed likely that this read originated from contamination of the assessed T_1_ sample. For the T_1_ progeny of the T_0_ plant, WPT590‐4, no somatic mutations nor the presence of any wild‐type alleles was detected (Table S1). In conclusion, the WPT590‐1 and WPT590‐4 plants have fully mutated *GmDrb2a* (∆7‐bp and a ∆1‐bp) and *GmDrb2b* (∆7‐bp and a ∆4‐bp) alleles that result in the creation of in‐frame premature stop codons (Figure S8).

### Identification of null‐segregant and phenotype analysis of *drb2a/drb2b* mutant plants

**Figure 3:**
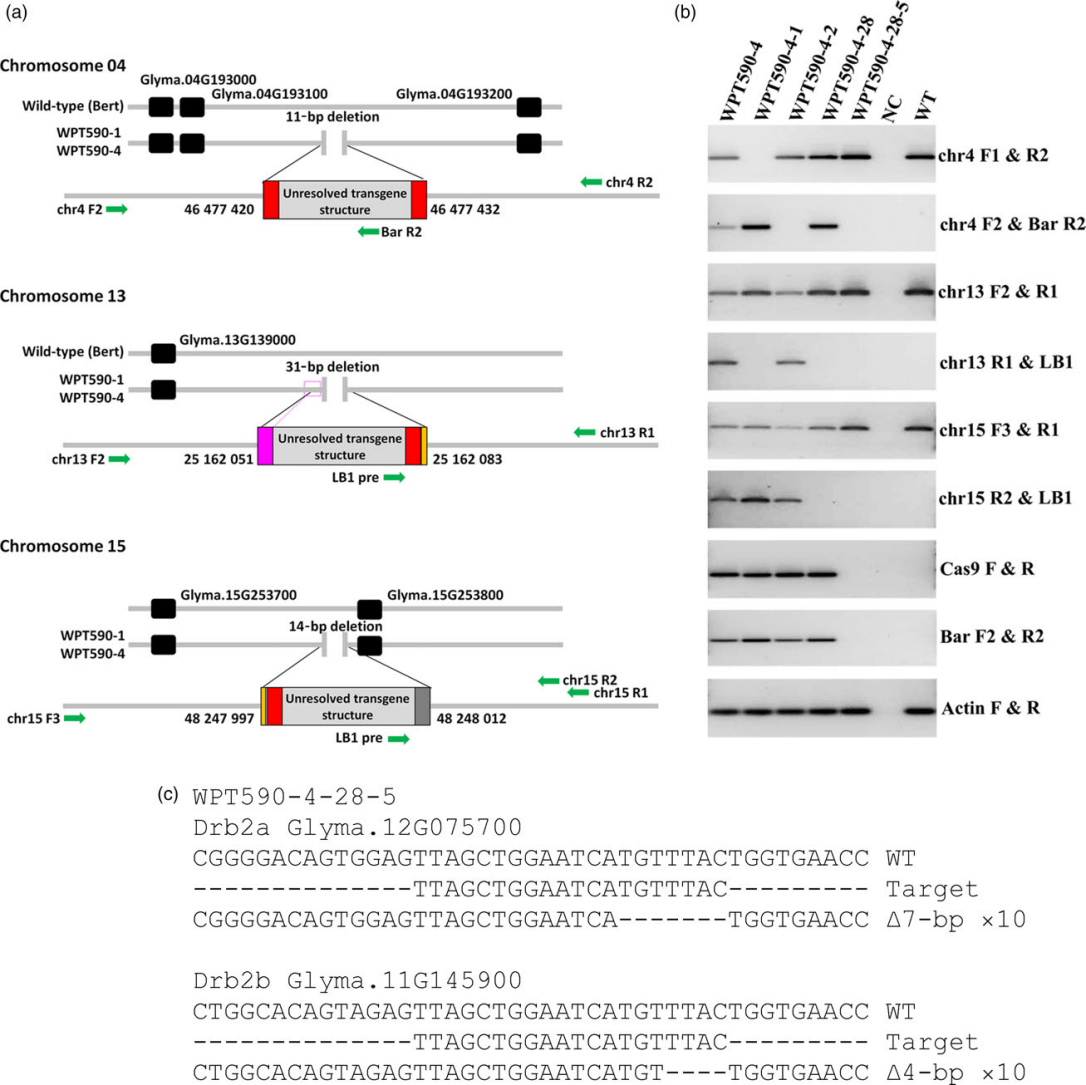
Integration sites and subsequent segregation of the CRISPR/Cas9 transgene targeting the *GmDrb2* loci. (a) WGS analysis identified three transgene integrations located on chromosomes four, thirteen and fifteen, respectively. Green arrows represent primers used to screen for null‐segregants (Table S5), the pink coloured box represents a duplicated region, the orange coloured box represents the DNA insertion, and the grey coloured rectangle represents the unknown sequence that does not return a BLASTn search result to any organism. (b) A PCR assay using transgene‐specific primers and primers spanning the genomic integration sites was used to screen for the removal of reagent transgenes by genetic segregation. No amplicons could be detected from the transgene corresponding to any of three transgene genomic locations in the WPT590‐4‐28‐5 plant. (c) The WPT590‐4‐28‐5 plant was sequence‐confirmed and found to be homozygous for the *drb2a* ^*Δ7*^ and *drb2b* ^*Δ4*^ mutant alleles, and termed the *Gmdrb2ab* double mutant.

Having established the double‐mutation transmission in the T_1_ progeny of WPT590‐1 and WPT590‐4 T_0_ plants, PCR‐based assays were next used to screen greater numbers (*n* ≈ 50) of each T_1_ generation to identify null‐segregant, transgene‐free plants. However, this approach failed to identify any null‐segregant plants (Figure S4b–d), suggesting that the WPT590‐1 and WPT590‐4 backgrounds harboured three or more transgenic events. We therefore used orphaned reads (reads where only one of the two paired ends map to the transgene cassette) generated as part of our WGS analysis to identify the genomic locations of transgene insertions in T_0_ plants. The WGS orphaned read approach failed to identify a transgene insertion event in the WPT590‐2 background. However, this approach identified three independent transgene insertions on soya bean chromosomes four, thirteen and fifteen in the WPT590‐1 and WPT590‐4 plants (Figure 3a). Each integration site was found adjacent to small chromosomal DNA deletions (ranging from 11 to 31 bp; Figures 3a and S9–S11) and three of the flanking regions included small genomic DNA duplication or insertion events (Figure 3a). Moreover, the three identified transgene insertions were mapped to identical genomic coordinates in the WPT590‐1 and WPT590‐4 backgrounds to again indicate that these two T_0_ plants were independent regenerants of the same transformation and mutagenesis event.

The genomic coordinates of each inserted transgene were next used to screen for null‐segregant T_1_ individuals and this approach revealed that T_1_ plant, WPT590‐4‐28, harboured only one of the three transgene insertions (Figure 3b). The applied screening also revealed that the transgene located on soya bean chromosome 4 was heterozygous (Figure 3b). Using the same approach to assess the T_2_ progeny of the T_1_ plant, WPT590‐4‐28, we identified a null‐segregant (WPT590‐4‐28‐5) that harboured no transgene‐derived sequences (Figure 3b) and confirmed via sequencing the mutant status of this plant (Figure 3c). A preliminary phenotype analysis was conducted on twenty‐four plants each of the WPT590‐4‐28 mutant (T1 progeny used in this analysis) and wild‐type ‘Bert’ backgrounds to assess the sensitivity/tolerance of the *drb2ab* double mutant to drought stress. Drought stress was selected as an abiotic stress of interest due to the large number of proteins associated with ‘drought stress’ or ‘osmotic stress’ responses demonstrated to have altered abundance following proteomic profiling of the *Arabidopsis drb2* (*Atdrb2*) mutant (Reis *et al*., 2015b). Reis *et al*. (2015b) demonstrated that *Atdrb2* plants were putatively more resistant to salt stress (specifically, 100 and 150 mm NaCl) than either wild‐type *Arabidopsis* (ecotype Columbia‐0; Col‐0), or the *Arabidopsis drb1* mutant. However, we observed that 21‐day‐old *Gmdrb2ab* mutant plants were significantly (*P* = 0.01) more sensitive to drought stress than wild‐type soya bean plants when water was withheld for a 7‐day period (Figure S12a). Scanning electron microscopy was next used to analyse the adaxial and abaxial leaf surfaces of 14‐day‐old plants, but this analysis failed to detect any obvious structural defects (Figure S12c). More specifically, the stomata of *Gmdrb2ab* and wild‐type leaves were determined to be of a similar size and had a comparable aperture. We also quantified the abundance of several microRNA (miRNA) species in the *Gmdrb2ab* double mutant due to this mutant line displaying dark green leaf coloration (Figure S5), a hallmark vegetative phenotype expressed by the *Atdrb2* mutant (Curtin *et al*., 2008; Eamens *et al*., 2012a). A stem‐loop primer‐based quantitative reverse transcription PCR (SL‐qPCR) approach was used to quantify the abundance of six miRNAs in the mutant and wild‐type backgrounds (Turner *et al*. 2013). Encouragingly, minor perturbations to the abundance of three of the six quantified miRNAs, miR156, miR163 and miR168, were detected in *GmDrb2ab* leaves (Figure S12b).

### Successful mutagenesis of the *GmDcl3a* locus followed by failure to transmit the mutation and transgene

**Figure 4:**
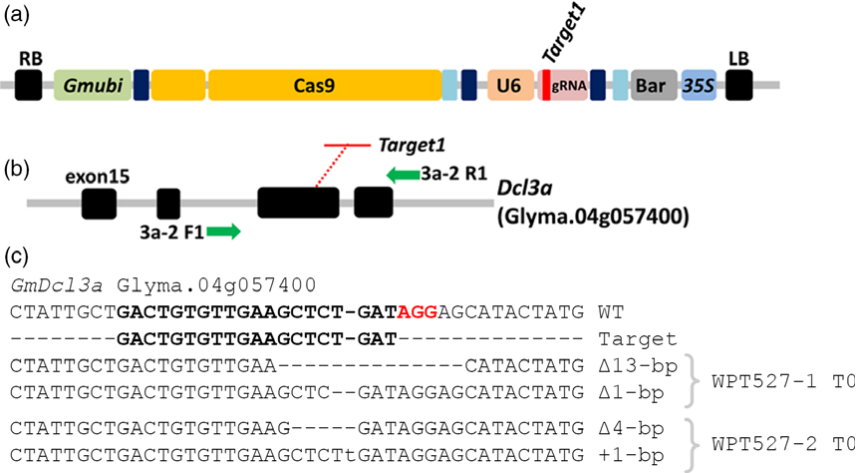
CRISPR/Cas9 mutagenesis of the soya bean *GmDcl3a* gene. (a) A schematic representation of the single gRNA CRISPR/Cas9 reagent targeting *GmDcl3a*. (b) The genomic arrangement of the *GmDcl3a* gene from exon 15 to exon 18, including the target site at exon 17. Green arrows represent primers used to screen mutant plants. (c) Sequence data generated from TACAS assays of the WPT527‐1 and WPT527‐2 T_0_ plants.

A *Gmubi* promoter‐driven CRISPR/Cas9 reagent with a single gRNA targeting the G*mDcl3a* locus was transformed into soya bean, and this transformation experiment generated five T_0_ plants (Figure 4a–b). Two T_0_ plants, WPT527‐1 and WPT527‐2, exhibited digestion‐resistant amplicons typical of germ‐line heterozygous mutations (Figure S13a). Sequencing of amplicons revealed ∆13‐bp and ∆1‐bp (~18%) and ∆4‐bp and ∆1‐bp (~12%) mutations to the *GmDcl3a* locus in T_0_ plants, WPT527‐1 and WPT527‐2, respectively (Figure 4c). Initial analysis of target site amplicons recovered mostly wild‐type alleles, rather than the expected 50% recovery of mutant amplicons normally associated with a heterozygous germ‐line mutation (Figure S14). We next screened the progeny of the WPT527‐1 and WPT527‐2 T_0_ plants (approximately 60 T_1_ plants from each T_0_ plant) and failed to confirm heritable transmission of any mutant allele, or the presence of the reagent transgene in either T_1_ population (Figure S13b). Whole‐genome sequencing was therefore next used to investigate each T_0_ event to determine both the fate of the mutant alleles, and to identify the genomic location of a putative reagent transgene. A comparable mutation frequency was obtained using WGS analysis to the TACAS assay for T_0_ plant, WPT527‐1 (17.7%) (Table S2; Figures S14 and S15) and mutations in the WPT527‐2 T_0_ plant were confirmed in both the *GmDcl3a* locus (9.1%) and the *GmDcl3b* pseudogene (17.8%) (Table S2; Figure S16). WGS orphaned reads identified a putative transgene location at Chr09: 36 274 816–36 275 314 for WPT527‐1; however, these same reads mapped to several genomic positions of low homology (Figure S17), and furthermore, a PCR‐based assay failed to detect any transgene sequences at the expected chromosome 9 coordinates (Figure S18). Finally, transgene analysis of the WPT527‐2 T_0_ plant line revealed low read counts and these lowly abundant reads failed to successfully map to any specific region of the soya bean genome.

### Targeted mutagenesis of the *Hen1* locus in soya bean and *Medicago truncatula*

**Figure 5:**
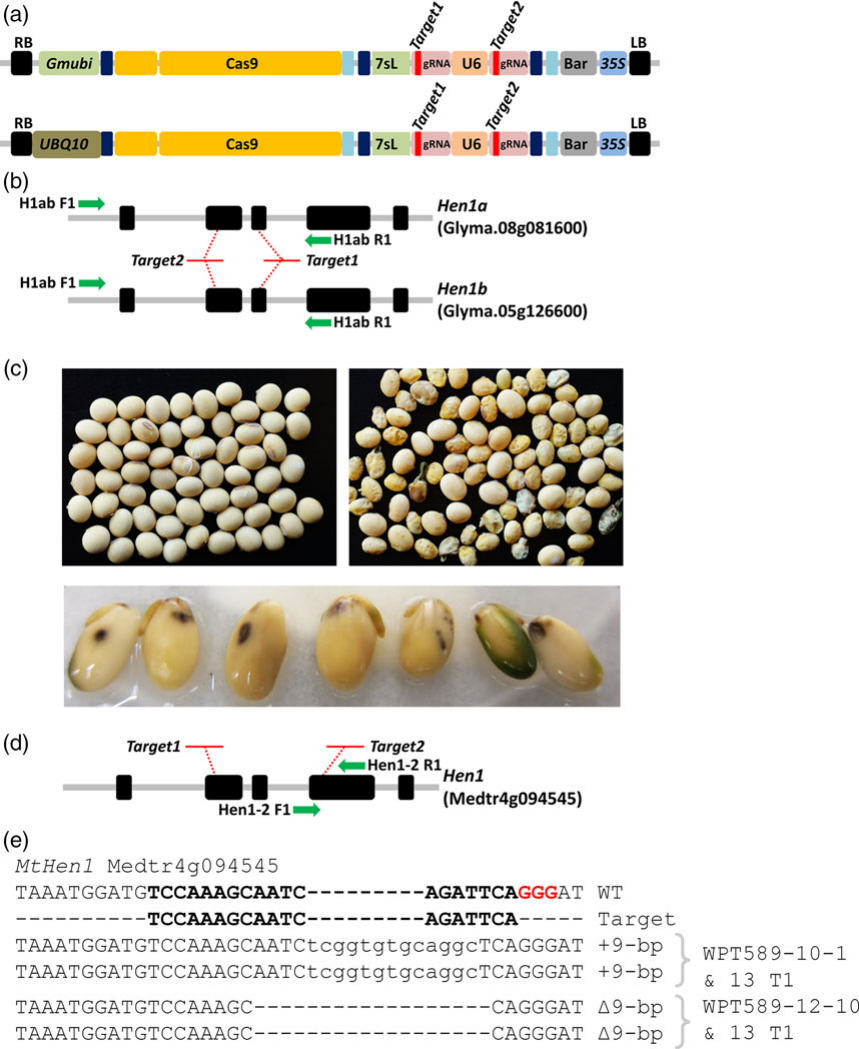
CRISPR/Cas9 mutagenesis of the soya bean and *Medicago truncatula Hen1* genes. (a) A schematic representation of the reagent transgene targeting two sites in both homeologue copies of the soya bean *Hen1* genes, *GmHen1a* and *GmHen1b*. The reagent transgene targeting sites in the single copy *M. truncatula Hen1* gene, *MtHen1*, uses the *At* UBQ10 promoter to drive Cas9 expression. (b) The genomic arrangement of the targeted soya bean *GmHen1a* and *GmHen1b* genes and the ‘*Target1*’ and ‘*Target2*’ sites. Green arrows represent primers used to screen mutant plants (c) The phenotypes of wild type (left) and the putative *Gmhen1ab* double‐mutant seed (right). Seeds with the shrunken developmental phenotype germinated poorly and did not progress past the stage of radicle emergence, and also displayed a mottled phenotype (bottom panel). (d) The genomic arrangement in *M. truncatula* gene, *MtHen1*, and the location of the ‘*Target1*’ and ‘*Target2*’ sites. Green arrows represent primers used to screen mutant plants. (e) The sequence confirmation of germ‐line mutations in *MtHen1* WPT222‐10 and WPT222‐12 mutant plants. The in‐frame mutations fail to disrupt the open‐reading frame of *Mthen1*, and no obvious *hen1*‐like mutant phenotype was observed.

A *Gmubi* and UBQ10 promoter‐driven CRISPR/Cas9 reagent, each containing two gRNAs targeting both homoeologue copies of soya bean *Hen1* locus (*GmHen1a*, Glyma.08g081600; *GmHen1b*, Glyma.05g126600) and two sites in the single copy *MtHen1* gene (Medtr4g094545) were transformed into soya bean and *M. truncatula* (Figure 5a–b). Two T_0_ soya bean plants, WPT589‐1 and WPT589‐2, were screened in tissue culture for the introduction of targeted mutations using a PCR digestion assay and the WPT589‐1 plant exhibited digest‐resistant amplicons typical of a germ‐line heterozygous mutation at both the *GmHen1a* and *GmHen1b* locus (Figure S18a–d). However, this plant failed to progress through the subsequent stages of the tissue culturing process; therefore, we could not collect seed for additional analyses in the subsequent generations. The second plant, WPT589‐2, initially failed to exhibit strong evidence for germ‐line mutation. However, faint digestion‐resistant bands indicative of somatic mutations were observed in the target amplicons for both *GmHen1a* and *GmHen1b* (Figure S19a–d). The WPT589‐2 plant was recovered from tissue culture and allowed to self‐fertilize to obtain seed for further analysis. A shrunken, shrivelled seed phenotype, a phenotype similar to that expressed by the *Gmdcl1a/dcl1b* double mutant, was observed in approximately half of the T_1_ seed obtained from the WPT598‐2 T_0_ plant (Figure 5c). We next attempted to screen the T_1_ progeny of the WPT589‐2 T_0_ plant for introduced mutations. However, this was unsuccessful due to our inability to obtain viable seedlings from these developmentally defective seeds, as only imbibition and radical emergence were observed (Figure 5c). Intriguingly, we observed the appearance of seed mottling upon imbibition, a trait typically associated small RNA‐mediated suppression from virus infection and most recently described in soya bean plants defective in ARGONAUTE5 (AGO5) activity (Figure 5c; Cho *et al*., 2017; Senda *et al*., 2004). We next screened seeds exhibiting the mottling phenotype to identify mutations in the *GmHen1a* or *GmHen1b* loci by performing both a TACAS and heteroduplex assay across both target sites using enriched and unenriched template; however, no evidence of mutagenesis was observed (Figure S19c–d).

Twelve *M. truncatula* T_0_ plants were recovered from tissue culture and four T_0_ plants, namely WPT222‐1, WPT222‐6, WPT222‐10 and WPT222‐12, returned digestion‐resistant amplicons indicative of germ‐line heterozygous mutations. The progeny of these four plants were screened and segregating germ‐line mutations were confirmed in the WPT222‐1, WPT222‐10 and WPT222‐12 T_1_ plants (Figures 5d,e and S20). Multiple WPT222‐1 and WPT222‐12 T_1_ plants were found to harbour an identical ∆9‐bp deletion, suggesting that these plants originated from the same transgenic event (Figure 5e). Screening of WPT222‐10 T_1_ plants confirmed a +9‐bp insertion (Figure 5e), and null‐segregant plants were identified from each line at a frequency that suggested each was the result of a single transgene insertion events (Figure S20).

### Targeted mutagenesis of soya bean genes using TAL‐effector nuclease (TALEN)

**Figure 6:**
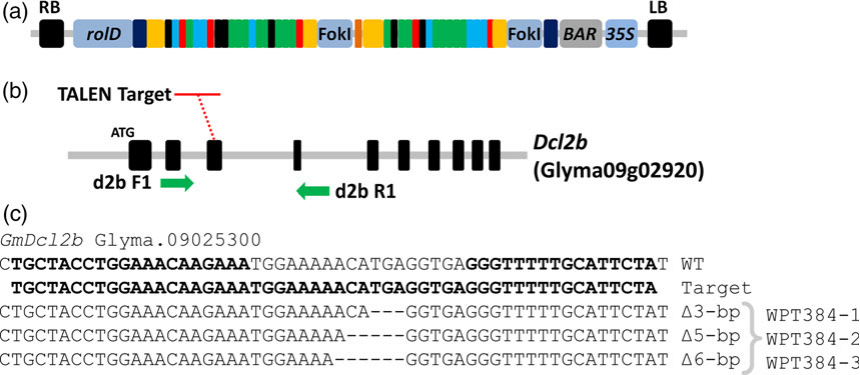
TALEN mutagenesis of the soya bean *GmDcl2b* gene. (a) The schematic representation of the *GmDcl2b* TALEN reagent expressed from the *A. rhizogenes rol*D promoter. (b) The genomic arrangement of *GmDcl2b* and the target site at the third exon. The green arrows indicate PCR primer locations used to screen mutants. (c) The sequence data from target amplicons of three T_0_ plants show targeted mutations. The sequences labelled as ‘*Target*’ indicate the 54‐bp TALEN target sequence.

In previous work, the soya bean *dicer‐like* genes *GmDcl2a*,* GmDcl2b* and *GmDcl3a* proved recalcitrant to mutagenesis using the zinc‐finger nuclease platform. Furthermore, and as outlined above, we also failed to generate a transmissible allele for *GmDcl3a* using the CRISPR/Cas9 platform. Therefore, we next attempted to use a TALEN platform to generate mutations in each of these soya bean *Dcl* genes. TAL arrays that showed a significant cleavage activity were cloned into a *rolD* expression vector (pSC218RG) and introduced into soya bean using a whole‐plant *Agrobacterium*‐mediated transformation assay (Table S3; Figure 6a). In total, 25 herbicide‐resistant T_0_ plants were recovered from tissue culture and included (i) 20 plants from the *GmDcl2a* TALEN transformation (plants WPT383‐1 to WPT383‐20), (ii) 4 plants from the *GmDcl2b* TALEN transformation (plants WPT384‐1 to WPT384‐4) and (iii) 1 plant from the *GmDcl3a* TALEN transformation, the WPT423‐1 T_0_ plant. Screening of *GmDcl2a* and *GmDcl3a* T_0_ plants failed to identify mutations at the targeted loci. However, three of the four T_0_ plants targeting *Dcl2b* (WPT384‐1, WPT384‐2 and WPT384‐3) exhibited digestion‐resistant amplicons indicative of a mutated target sequence (Figures 6b and S21a–b). Amplicons from the *Dcl2b* target site were sequenced and monoallelic deletions of ∆3‐bp for plant WPT384‐1, ∆5‐bp for plant WPT384‐2 and ∆6‐bp for plant WPT384‐3 were confirmed (Figure 6c). T_0_ plants were self‐fertilized, and their T_1_ progeny screened to confirm heritable transmission of the mutagenesis transgenes. Sequencing of the target sites in T_1_ progeny of the WPT384‐1 T_0_ plant confirmed segregation of the ∆3‐bp deletion, with six heterozygous, four homozygous and two wild‐type plants, including two transgene‐free heterozygous lines, plants WPT384‐1‐8 and WPT384‐1‐12 (Figure S22). These results confirmed the previous findings of Haun *et al*. (2014) and Demorest *et al*. (2016) that TALENs can generate nontransgenic heritable mutant plants in soya bean. A screening of T_1_ progeny from the T_0_ plants, WPT384‐2 and WPT384‐3, failed to confirm heritable transmission of the T_0_ mutations. Instead, screening identified mostly wild‐type genotypes and some somatic mutations (Figure S23).

### Generating combinatorial soya bean mutant plants by cross‐fertilization

**Figure 7:**
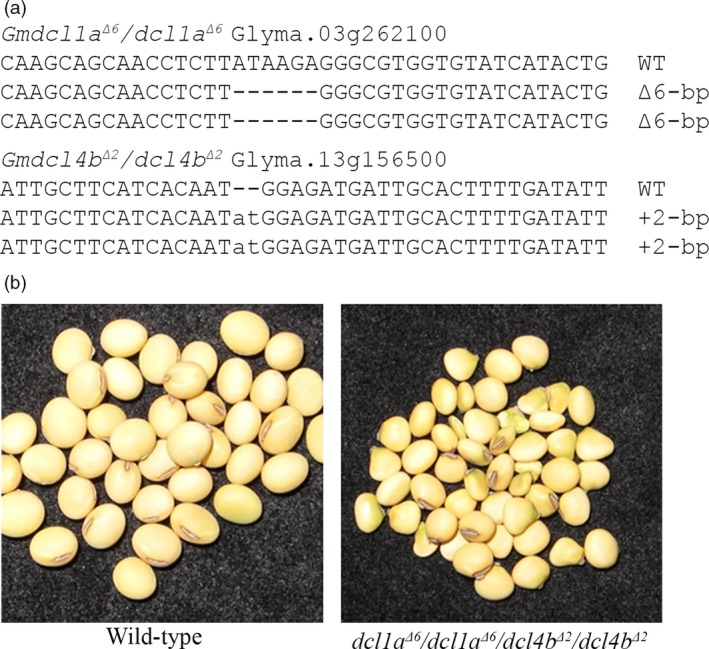
Combining soya bean *dicer‐like* mutated alleles. (a) Sequence confirmation of a double mutant developed via a standard genetic crossing approach using the confirmed soya bean *dcl1a* mutant line and the *dcl4b* mutant plant. (b) In a preliminary phenotype analysis, the *dcl1a* ^*Δ6*^ */dcl1a* ^*Δ6*^ */dcl4b* ^*Δ2*^ */dcl4b* ^*Δ2*^ double‐mutant seed has a significantly reduced seed weight compared with wild type.

We have previously reported the generation of single nontransgenic *Gmdcl1a*,* Gmdcl1b* and *Gmdcl4b* soya bean mutants via the use of the CoDA ZFN platform (Curtin *et al*., 2011, 2016). Mutant alleles from two nontransgenic plants, namely DCL4a/DCL4a/*dcl4b* ^Δ2^/*dcl4b* ^Δ2^ and *dcl1a* ^*Δ7*^/*dcl1a* ^*Δ6*^/*DCL1b/dcl1b* ^*Δ3*^, were combined by cross‐fertilization to generate a combinatorial *Gmdcl1a/dcl4b* double mutant (Figure S24a; Method S2). The resulting seeds were bulked, and the mutant status of F_1_ plants was confirmed by a PCR digestion assay that confirmed wild‐type, *dcl1a* ^*Δ6*^ and *dcl4b* ^*Δ2*^ allelic backgrounds (Figure S24b). The F_2_ progeny of these F_1_ plants were next screened and the homozygous double mutant was identified (Figures 7a and S24–S25). A preliminary phenotypic analysis comparing seed weight of the mutant and wild‐type plants was determined to be significantly reduced in the double mutant (*P* < 0.00001). These results demonstrate that the stacking of single mutants to generate a combinatorial *dcl1a/dcl4b* double mutant is a successful approach for the generation of novel mutant backgrounds that display unique phenotypes. We next combined the *Gmdcl4b* ^*Δ2*^ and an additional *Gmdcl1a* ^*Δ7*^ and *Gmdcl1b* ^*Δ15*^ mutant alleles with the *Gmdrb2a* ^*Δ7*^ */drb2a* ^*Δ1*^/*drb2b* ^*Δ7*^ */drb2b* ^*Δ4*^ plant, the *Gmdrb2ab* double mutant, described above (Figures S26–S30). The F_1_ plants resulting from this crossing event had their mutational status confirmed by a sequence‐based approach and the F_2_ progeny bulked for the identification of single, double and triple mutant combinations of *Gmdcl1*,* Gmdcl1b*,* Gmdcl4b*,* Gmdrb2a* and *Gmdrb2b* plants (Table 1).

**Table 1:** The generation of combinatorial *dcl1ab/drb2ab* and *dcl4b/drb2ab* mutant plants

Reference	Direction of cross	Mutant alleles	Mutants
M13‐719	*dcl1a* × *dcl4b*	*dcl1a* ^*Δ6*^, *dcl4b* ^*Δ2*^	*dcl1a/dcl4b‐1* b
M17‐725	*drb2ab* × *wt*	*drb2a* ^*Δ1*^, *drb2b* ^*Δ4*^	*drb2a‐1*,* drb2b‐1* b
M17‐726	*wt* × *drb2ab*	N.D.	
M17‐727	*drb2ab* × *dcl1b*	*drb2a* ^*Δ1*^, *drb2b* ^*Δ4*^, *dcl1b* ^*Δ15*^	*drb2a‐1*,* drb2b‐1* b, *drb2a/dcl1b‐1* b, *drb2b/dcl1b‐1* b, *drb2a/drb2b/dcl1b‐1* b
M17‐728	*dcl1b* × *drb2ab*	N.D.	
M17‐729	*drb2ab* × *dcl4b*	*drb2a* ^*Δ7*^, *drb2b* ^*Δ7*^, *dcl4b* ^*Δ2*^	*drb2a‐2*,* drb2b‐2* b, *drb2a/dcl4b‐1* b, *drb2b/dcl4b‐1* b, *drb2a/drb2b/dcl4b‐1* b
M17‐730	*dcl4b* × *drb2ab* a	N.D.	
M17‐733	*dcl1a* × *drb2ab* a	*drb2a* ^*Δ7*^, *drb2b* ^*Δ4*^, *dcl1a* ^*Δ7*^	*drb2a‐2*,* drb2b‐1*,* drb2a/dcl1a‐1*,* drb2b/dcl1a‐1*,* drb2a/drb2b/dcl1a‐1*
M17‐734	*dcl1a* × *drb2ab* a	N.D.	
M17‐735	*dcl4b* × *dcl1a* a	*dcl1a* ^*Δ7*^, *dcl4b* ^*Δ2*^	*dcl1a/dcl4b‐2*
M17‐736	*dcl1a* × *dcl4b* a	N.D.	

N.D. not determined.

a M_1_ screening in progress.

b M_2_ screening in progress.

## Discussion

Here, we report the generation of targeted mutations in loci central to the small RNA pathways of soya bean and *M. truncatula* using the ZFN, TALEN and CRISPR/Cas9 nuclease platforms. This approach enabled the recovery of a wide range of stable, nontransgenic plants, including those with in‐frame, frame‐shift, single homozygous and double homozygous mutations. The CRISPR/Cas9 constructs proved straightforward to produce and were efficient at generating targeted mutations *in planta* in both species. One of the key findings of this study was the ability of CRISPR/Cas9 reagents to generate double mutants with fully mutated alleles in the initial T_0_ generation, as evidenced by the *GmDrb2a* and *GmDrb2b* paralog pair. Soya bean is a highly duplicated palaeo‐polyploid plant where approximately 70% of the protein‐coding sequences encoded by the genome are further represented by a duplicated copy (Schmutz *et al*., 2010). An important requirement for any reagent designed for use in soya bean is the ability to simultaneously mutate multiple gene copies in a single transformation event. While the capacity of the CRISPR/Cas9 reagent to readily generate mutants is quite remarkable, we have also identified double mutants in a single generation using the ZFN platform (Curtin *et al*., 2016). However, ZFNs have a reduced workable target range in most plant species and require considerably more effort to design and construct (Sander *et al*., 2011).

We also report here on the generation of two T_0_ double bi‐allelic mutations of the *GmDrb2a* and *GmDrb2b* loci, and further, heritable transmission of these mutations was demonstrated in T_1_ progeny. The mutated alleles in these two plants were identical (this was true for both gene targets, *GmDrb2a* and *GmDrb2b*), and at least one CRISPR/Cas9 transgene insertion was transmitted to all T_1_ progeny. Our initial suspicion that these plants arose from the same transgenic explants was confirmed by WGS analysis that identified three transgene insertions in exactly the same genomic coordinates in both T_0_ plants. Using these genomic coordinates, null‐segregant transgene‐free plants were identified and deregulated article status granted by USDA‐APHIS, thereby paving the way for future field testing of this line (https://www.aphis.usda.gov/aphis/ourfocus/biotechnology/am-i-regulated) (Figure S31). The *drb2ab* double‐mutant plant will also be a valuable genetic resource for studying the function of DRB2 function in soya bean, having previously been demonstrated to play functionally diverse roles in multiple small RNA‐directed RNA silencing pathways in *Arabidopsis*, including the production stages of the repeat‐associated small‐interfering RNA (rasiRNAs) and *trans*‐acting small‐interfering RNAs (tasiRNAs) pathways (Pelissier *et al*., 2011) and the inhibition of viral replication (Barton *et al*., 2017). *Arabidopsis* DRB2 has also been shown to be an important requirement for the production of a cohort of miRNAs in developmentally important *Arabidopsis* tissues, namely the shoot apex and maturing pollen (Eamens *et al*., 2012a,b). Many of the target genes of this *At*DRB2‐dependent miRNA cohort appear to be involved in stress responses and are not involved in normal plant development pathways (Reis *et al*., 2015b). Therefore, in view of these findings, the generation of the *Gmdrb2ab* double mutant provides an excellent opportunity to characterize miRNA‐directed responses to stress in soya bean and for the subsequent comparison of *Gm*DRB2 function to the well‐documented functionality of the *At*DRB2 protein.

The transgenesis and mutagenesis of the *GmDcl3a* locus with our CRISPR/Cas9 reagent generated curious results. First, we identified two putative T_0_ mutant plants that initially appeared to be germ‐line heterozygous mutants (Figure S13a). However, the quantity of mutated amplicons obtained was significantly less than that expected for a heterozygous mutant plant, which normally returns an approximate mutant to wild‐type amplicon ratio of 1 : 1 (i.e. 50% mutant and 50% wild‐type amplicons). Secondly, and most importantly, neither the targeted mutation nor the introduced transgene was heritably transmitted to the T_1_ generation (Figure S13b). We used the generated WGS data, specifically orphaned reads, to identify the genomic location of the transgene insertion on chromosome nine for the WPT527‐1 T_0_ plant. However, we failed to identify a transgene insertion for the WPT527‐2 T_0_ plant, despite orphaned reads mapping to the transgene at a very low frequency. There are at least three possible explanations for this finding: (i) the detected T‐DNA was present at contaminant levels; (ii) the T‐DNA was integrated into the genome; however, a stochastic process led to poor sequencing coverage; or (iii) the T‐DNA was integrated in only a small number of cells, and this cell number was insufficient for reliable detection at 20× genome coverage. Presently, insufficient data are available to make a conclusive statement on the exact reason(s) why both *GmDcl3a* mutations were not heritably transmitted.

We also targeted the *Hen1a* and *Hen1b* homeologue copies in soya bean and the single *Hen1* copy in *M. truncatula* in an attempt to generate mutations in these three HEN1 encoding loci. In soya bean, we identified one plant that appeared to harbour mutations in both the *Hen1a* and *Hen1b* locus; however, this plant failed to survive through the tissue culture. A second plant was successfully recovered, and the resulting T_1_ progeny exhibited a seed mottling phenotype typical of small RNA perturbation. Moreover, these seeds failed to fully germinate, and we could not conclusively identify germ‐line mutations using either an enriched or nonenriched PCR template. The screening of *M. truncatula* T_1_ plants identified three mutants each with transmittable in‐frame mutations that likely do not disrupt gene function. Furthermore, none of the T_1_ plants exhibited the striking pleiotropic phenotypes associated with mutation of the *hen1* locus in *Arabidopsis* and rice (Abe *et al*., 2010; Chen *et al*., 2002). Using an identical reagent platform to introduce mutations to other targeted *M. truncatula* genes, we have previously observed high mutagenesis rates of 50%–75% in the T_0_ generation plants (Curtin *et al*., 2017). It was therefore surprising that only in‐frame mutations were recovered at reduced mutagenesis efficiencies. Taken together, these results suggest that a heterozygous frame‐shift knockout of *Hen1* may be embryo lethal in legumes.

The TALENs and CRISPR/Cas9 platforms are complementary genome engineering technologies, with the CRISPR/Cas9 platform recently emerging as the platform of choice for most applications. The TALEN platform has a limitless targeting range that might be advantageous for targeting a highly specific locus (Joung and Sander, 2013). However, the striking disadvantage of the TALEN approach, in our limited experience in soya bean, is the inferior mutational efficiency offered by this platform. Here, nine TALEN constructs that targeted three *Dcl* loci (*GmDcl2a*,* GmDcl2b* and *GmDcl3a*) were engineered. However, three of these reagents exhibited activity in yeast and just one producing a transmittable mutation. In comparison, each of the four CRISPR/Cas9 constructs used in this study showed mutation activity in whole plants and two of them produced transmittable mutations.

A combinatorial *Gmdcl1a/dcl4b* mutant plant was identified by combining single *Gmdcl1a* and *Gmdcl4b* mutants previously generated via the use of the ZFN platform. A similar approach has been used in *Arabidopsis* to establish both hierarchical and redundant roles for each of the five members of the *DRB* gene family in the parallel RNA silencing pathways (Curtin *et al*., 2008; Eamens *et al*., 2009, 2012a,b). We also confirmed the functional activity of *GmDcl2b* and *GmDcl3a* TALEN and CRISPR/Cas9 reagents that could be introduced into the nontransgenic *Gmdcl1a/dcl4b* double mutant to quickly combine additional mutations. Similarly, the *Gmdrb2ab* double mutant was crossed with *Gmdcl1a*,* Gmdcl1b* and *Gmdcl4b* single mutants, and single, double and triple mutant combinations are currently being screened. The generation of this particular suite of soya bean mutant germplasm is of high priority when *Arabidopsis* research is considered; that is, *At*DRB2 appears to be able to bind both imperfectly and perfectly dsRNA templates, and to form functional partnerships with *At*DCL1 and *At*DCL4 for miRNA and siRNA (rasiRNAs and tasiRNAs) production, respectively (Eamens *et al*., 2012a; Pelissier *et al*., 2011; Reis *et al*., 2015a). Therefore, this collection of small RNA‐associated mutants forms a valuable genetic resource, not only for the legume research community but also for the greater plant biology community. Several recent reports have used whole‐genome, RNA and small RNA sequence analyses to document the roles played by small RNA‐directed gene expression regulation in both development and response to various environmental stresses (Arikit *et al*., 2014; Curtin *et al*., 2012; Zhai *et al*., 2011). To date however, small RNA studies in plant species outside of *Arabidopsis* have been largely limited to cataloging alterations in the accumulation of various species of small RNAs in developmentally distinct tissues, or after exposure to stress. Therefore, in legumes, as in other plant species, there has been a conspicuous lack of mutant analyses to establish gene function for loci encoding the orthologous central machinery proteins involved in either the production or activity stages of small RNA‐directed RNA silencing pathways.

Genome engineering reagents are rapidly becoming central tools for legume functional genomic studies as well as being used as an alternate approach to improve the soya bean germplasm (Cai *et al*., 2017; Curtin *et al*., 2017; Demorest *et al*., 2016). The limitations for targeted mutagenesis are no longer a result of inefficient genome engineering reagents. The bottleneck now appears to be the current plant transformation technologies that are highly labour intensive. Future efforts to overcome such barriers will come from the development of nontransgenic delivery methods that utilize morphogenic regulators to increase the efficiency and ease of transformation (Lowe *et al*., 2016; Mookkan *et al*., 2017). Another approach could deliver gRNAs directly to the plant that constitutively expresses Cas9 (Figure S32a–c). The ZFN, TALEN and CRISPR/Cas9 SSN platforms comprise a genome engineering toolkit that will be useful for future functional genomics projects in soya bean and in other plant species. The efficiency, multiplex capabilities and increased overall activity of the CRISPR/Cas9 platform will likely make it the preferred SSN. However, certain scenarios may arise, such as the targeting of a highly specific genomic sequence for which no CRISPR/Cas9 or ZFN target could be identified, and in this case, the TALEN platform may provide an alternate route to mutant generation.

## Material and methods

### Generation of plant expression vectors for site‐specific nuclease delivery

The plant expression vectors were constructed using a previously reported binary vector pNB96 (Curtin *et al*., 2011). A 35S::OCS cassette from pArt7 was cloned into the NotI sites of the T‐DNA alongside the 35S::BAR cassette for phosphinothricin herbicide selection (pSC218). The pSC218 vector was digested with SacII and XhoI to remove the 35S promoter and replaced with either *Gmubi*,* At*UBQ or *rol*D promoters that were amplified from templates using primers with SacII and XhoI overhangs (Grefen *et al*., 2010; Hernandez‐Garcia *et al*., 2010; Wally *et al*., 2008). The Gateway™ reading frame cassette was removed from the pTDTO (Valdes‐Lopez *et al*., 2008) vectors by XhoI and SpeI digestion and cloned into the XhoI and XbaI sites to generate the pSC218GG, pSC218UG and pSC218RG vectors. The resulting plant expression vectors were used for delivery of the CRISPR/Cas9 or the TAL‐effector nuclease reagents.

### Hairy‐root and whole‐plant transformation assays of soya bean and *M. truncatula*

*Agrobacterium rhizogenes* strain, K599, was used for rapid generation of transgenic plant tissue via a soya bean hairy‐root *ex vitro* transformation assay (Figure S2) (Curtin *et al*., 2011; Taylor *et al*., 2006). The disarmed *A. rhizogenes* strain, 18r12, was used for a previously reported whole‐plant transformation assay (Curtin *et al*., 2011; Paz *et al*., 2006). All whole‐plant soya bean transformations were performed in the same genetic background, the Bert cultivar. *Medicago truncatula* whole‐plant transformation was carried out in the cultivar, R108 (HM340), using the *A. tumefaciens* strain, EAH105 (Table S7; Cosson *et al*., 2006; Curtin *et al*., 2017).

### CRISPR/Cas9 design and assembly

Targets sites were identified either by the ‘find’ function with the target template (G‐N^20^—GG) in VectoNTI (Life Technologies, CA) or were designed using the Broad Institute's sgRNA designer website (Table S4) (Doench *et al*., 2014). Guide RNAs for each target were engineered using a PNK oligo annealing assay and cloned into three binary vectors using Gateway™ cloning reactions. A detailed assembly method has been previously published (Curtin *et al*., 2017).

### PCR, target amplicon cloning and the sequence (TACAS) and heteroduplex assays

All PCRs were carried out using the Hot Start Plus master mix (Qiagen) according to the manufacturer's guidelines. For the TACAS assay, PCR amplicons were purified using the QIAquick PCR Purification Kit (Qiagen) and cloned into pGem‐T‐Easy cloning vector (Promega). Colony PCR amplicons were diluted 1 : 12 with sterile deionized water and directly sequenced. The heteroduplex assay has been previously reported (Jacobs *et al*., 2015; Zhu *et al*., 2014) and can viewed online under the Method S3 section. Primers used in this report are provided in Table S5.

### Analysis of transgene insertion sites and targeted and off‐target mutations

The whole‐genome sequence (WGS) analyses used to identify mutations at targeted loci and to detect the genomic location of transgene integration sites have been previously reported (Anderson *et al*., 2016; Srivastava *et al*., 2014). A more detailed description of this protocol can be found online in the Method S4. Putative off‐target mutagenesis activity in transformed plants was manually screened using WGS data, specifically via manually analysing the generated list of potential off‐target sites for each construct. The list was generated using the CRISPR‐P site (http://cbi.hzau.edu.cn/crispr/) (Table S6; Lei *et al*., 2014).

### Phenotypic characterization of mutants

Detailed methods for characterization mutant of phenotypes that include drought assay, SL‐qPCR and SEM sample preparation can be viewed online under the Method S5.

## Conflict of interest

Čermák and Voytas are named inventors on several patents involving the use of TALENs to create targeted genome modifications.

## Supporting information


**Figure S1** The plant expression vectors used for the delivery of CRISPR/Cas9 and TALEN site‐specific nucleases.
**Figure S2** Hairy‐root transformation protocol for rapid introduction of transgenes into soybean.
**Figure S3** The yeast cleavage assay used to screen TAL‐effector nuclease activity.
**Figure S4** PCR‐digest and hetero‐duplex gel analysis of WPT590 soybean plants.
**Figure S5** TACAS assay mutation data for WPT590‐1, WPT590‐2 and WPT590‐4 plants.
**Figure S6** Whole genome sequence analysis of Drb2a (Glyma.12g075700) target sites in Bert, WPT590‐1, WPT590‐1‐1, WPT590‐1‐2, WPT590‐2, WPT590‐4, WPT590‐4‐1 and WPT590‐4‐2 plants.
**Figure S7** Whole genome sequence analysis of Drb2b (Glyma.11g145900) target sites in Bert, WPT590‐1, WPT590‐1‐1, WPT590‐1‐2, WPT590‐2, WPT590‐4, WPT590‐4‐1 and WPT590‐4‐2 plants.
**Figure S8** The target site encodes the start of the highly conserved second double‐stranded RNA‐binding motif (dsRBM).
**Figure S9** Coverage of reads mapping to the reference genome surrounding transgene at chromosome 4.
**Figure S10** Coverage of reads mapping to the reference genome surrounding transgene at chromosome 13.
**Figure S11** Coverage of reads mapping to the reference genome surrounding transgene at chromosome 15.
**Figure S12** Phenotype analysis of the *drb2ab* mutant.
**Figure S13** Screening of T_0_ and T_1_ soybean plants for CRISPR‐mediated targeted mutations at *Dcl3a*.
**Figure S14** TACAS sequence data of the *Dcl3a* T_0_ mutant plants.
**Figure S15** The WGS analysis of mutations at Glyma.04g057400 in the WPT527‐1 & WPT527‐2 plants.
**Figure S16** The WGS analysis of mutations at Glyma.06G05800 in the WPT527‐1 & WPT527‐2 plants.
**Figure S17** WGS paired end reads mapping to the putative genomic location *the Dcl3a* reagent in WPT527‐1 and WPT 527‐2 T0 plant.
**Figure S18** PCR assay of the putative *Dcl3a* reagent at the chromosome 9 locus.
**Figure S19** PCR‐digestion assay of *GmHen1a* and *GmHen1b* WPT589‐1 and WPT 589‐2 T_0_ plants.
**Figure S20** Screening of *Mt Hen1* T_1_ mutant plants by PCR digestion assay.
**Figure S21** PCR‐digestion assays of T_0_ plants from samples taken from different parts of the plant harboring the TALEN targeting *Dcl2b* plants.
**Figure S22** The heritable transmission of *Dcl2b* mutation in WPT384‐1 T_1_ plants and removal of the transgene by genetic segregation.
**Figure S23** The heritable transmission of the *Dcl2b* mutations was not observed in WPT384‐2 and WPT384‐3 T_0_ plants.
**Figure S24** Combining *dcl1a* and *dcl4b* mutations.
**Figure S25** The *dcl1a* ^*Δ6*^ */dcl1a* ^*Δ6*^ */dcl4b* ^*Δ2*^ */dcl4b* ^*Δ2*^ and wild‐type plant.
**Figure S26** *Gmdrb2a* ^*Δ7*^ */drb2a* ^*Δ1*^/*drb2b* ^*Δ7*^ */drb2b* ^*Δ4*^ cross to wild type to recover single *drb2a* and *drb2b* mutant plants.
**Figure S27** Combining the *Gmdrb2a* ^*Δ7*^ */drb2a* ^*Δ1*^/*drb2b* ^*Δ7*^ */drb2b* ^*Δ4*^ and *Gmdcl1b* ^*Δ15*^ mutant alleles.
**Figure S28** Combining the *Gmdrb2a* ^*Δ7*^ */drb2a* ^*Δ1*^/*drb2b* ^*Δ7*^ */drb2b* ^*Δ4*^ and *Gmdcl4b* ^*Δ2*^ mutant alleles.
**Figure S29** Combining the *Gmdrb2a* ^*Δ7*^ */drb2a* ^*Δ1*^/*drb2b* ^*Δ7*^ */drb2b* ^*Δ4*^ and *Gmdcl1a* ^*Δ7*^ mutant alleles.
**Figure S30** Combining the *Gmdcl4b* ^*Δ2*^ and *Gmdcl1a* ^*Δ7*^ mutant alleles.
**Figure S31** USDA‐APHIS confirmation that *Glycine max* (soybean) line WPT590‐4‐28‐5 is not a regulated article.
**Figure S32** The identification of Cas9 over‐expression cassettes in whole soybean and *M. truncatula* plants.
**Method S1** TALEN design and assembly.
**Method S2** Soybean genetic hybridization assay.
**Method S3** Heteroduplex assay for detection of targeted mutations.
**Method S4** Identifying transgene junctions and CRIPSR deletions.
**Method S5** Phenotypic characterization of mutants.

**Table S1** Analysis of WGS reads form WPT590‐1, WPT590‐2 and WPT590‐4.

**Table S2** Analysis of WGS reads form WPT527‐1 and WPT527‐2.

**Table S3** List of TAL‐effect nuclease RVD binding arrays and gene targets.

**Table S4** List of CRISPR gRNA sequences and gene targets.

**Table S5** Primers used in this study.

**Table S6** Potential off‐target sites generated for *Dcl3a*,* Drb2a* and *Drb2b* using CRISPR‐P.

**Table S7** List of reagents and their targets in Medicago and soybean.
