# Supplementary material for: CRISPR/Cas9 and TALENs generate heritable mutations for genes involved in small RNA processing of Glycine max and Medicago truncatula
Source: Plant Biotechnol J. 2017 Dec 4;16(6):1125–37. doi: 10.1111/pbi.12857 (PMC5978873; doi:10.1111/pbi.12857)
Supplement: Supplementary file 1 — Figure S1 The plant expression vectors used for the delivery of CRISPR/Cas9 and TALEN site‐specific nucleases. Figure S2 Hairy‐root transformation protocol for rapid introduction of transgenes into soybean. Figure S3 The yeast cleavage assay used to screen TAL‐effector nuclease activity. Figure S4 PCR‐digest and hetero‐duplex gel analysis of WPT590 soybean plants. Figure S5 TACAS assay mutation data for WPT590‐1, WPT590‐2 and WPT590‐4 plants. Figure S6 Whole genome sequence analysis of Drb2a (Glyma.12g075700) target sites in Bert, WPT590‐1, WPT590‐1‐1, WPT590‐1‐2, WPT590‐2, WPT590‐4, WPT590‐4‐1 and WPT590‐4‐2 plants. Figure S7 Whole genome sequence analysis of Drb2b (Glyma.11g145900) target sites in Bert, WPT590‐1, WPT590‐1‐1, WPT590‐1‐2, WPT590‐2, WPT590‐4, WPT590‐4‐1 and WPT590‐4‐2 plants. Figure S8 The target site encodes the start of the highly conserved second double‐stranded RNA‐binding motif (dsRBM). Figure S9 Coverage of reads mapping to the reference genome surrounding transgene at chromosome 4. Figure S10 Coverage of reads mapping to the reference genome surrounding transgene at chromosome 13. Figure S11 Coverage of reads mapping to the reference genome surrounding transgene at chromosome 15. Figure S12 Phenotype analysis of the drb2ab mutant. Figure S13 Screening of T0 and T1 soybean plants for CRISPR‐mediated targeted mutations at Dcl3a. Figure S14 TACAS sequence data of the Dcl3a T0 mutant plants. Figure S15 The WGS analysis of mutations at Glyma.04g057400 in the WPT527‐1 & WPT527‐2 plants. Figure S16 The WGS analysis of mutations at Glyma.06G05800 in the WPT527‐1 & WPT527‐2 plants. Figure S17 WGS paired end reads mapping to the putative genomic location the Dcl3a reagent in WPT527‐1 and WPT 527‐2 T0 plant. Figure S18 PCR assay of the putative Dcl3a reagent at the chromosome 9 locus. Figure S19 PCR‐digestion assay of GmHen1a and GmHen1b WPT589‐1 and WPT 589‐2 T0 plants. Figure S20 Screening of Mt Hen1 T1 mutant plants by PCR digestion assay. Figure S21 [file PBI-16-1125-s004.docx]

**Supporting information (CURTIN, et al. 2017) “CRISPR/Cas9 and TALENs generate heritable mutations for genes involved in small RNA processing of *Glycine max* and *Medicago truncatula*”**

Additional Supporting Information may be found online in the supporting information tab for this article:

**Figure S1** The plant expression vectors used for the delivery of CRISPR/Cas9 and TALEN site-specific nucleases.

**Figure S2** Hairy-root transformation protocol for rapid introduction of transgenes into soybean

**Figure S3** The yeast cleavage assay used to screen TAL-effector nuclease activity

**Figure S4** PCR-digest and hetero-duplex gel analysis of WPT590 soybean plants.

**Figure S5** TACAS assay mutation data for WPT590-1, WPT590-2 and WPT590-4 plants

**Figure S6** Whole genome sequence analysis of Drb2a (Glyma.12g075700) target sites in Bert, WPT590-1, WPT590-1-1, WPT590-1-2, WPT590-2, WPT590-4, WPT590-4-1 and WPT590-4-2 plants.

**Figure S7** Whole genome sequence analysis of Drb2b (Glyma.11g145900) target sites in Bert, WPT590-1, WPT590-1-1, WPT590-1-2, WPT590-2, WPT590-4, WPT590-4-1 and WPT590-4-2 plants.

**Figure S8** The target site encodes the start of the highly conserved second double-stranded RNA-binding motif (dsRBM)

**Figure S9** Coverage of reads mapping to the reference genome surrounding transgene at chromosome 4

**Figure S10** Coverage of reads mapping to the reference genome surrounding transgene at chromosome 13

**Figure S11** Coverage of reads mapping to the reference genome surrounding transgene at chromosome 15

**Figure S12** Phenotype analysis of the *drb2ab* mutant.

**Figure S13** Screening of T_0_ and T_1_ soybean plants for CRISPR-mediated targeted mutations at *Dcl3a*

**Figure S14** TACAS sequence data of the *Dcl3a* T_0_ mutant plants

**Figure S15** The WGS analysis of mutations at Glyma.04g057400 in the WPT527-1 & WPT527-2 plants

**Figure S16** The WGS analysis of mutations at Glyma.06G05800 in the WPT527-1 & WPT527-2 plants

**Figure S17** WGS paired end reads mapping to the putative genomic location *the Dcl3a* reagent in WPT527-1 and WPT 527-2 T0 plant

**Figure S18** PCR assay of the putative *Dcl3a* reagent at the chromosome 9 locus

**Figure S19** PCR-digestion assay of *GmHen1a* and *GmHen1b* WPT589-1 and WPT 589-2 T_0_ plants

**Figure S20** Screening of *Mt Hen1* T_1_ mutant plants by PCR digestion assay

**Figure S21** PCR-digestion assa­­ys of T_0_ plants from samples taken from different parts of the plant harboring the TALEN targeting *Dcl2b* plants

**Figure S22** The heritable transmission of *Dcl2b* mutation in WPT384-1 T_1_ plants and removal of the transgene by genetic segregation

**Figure S23** The heritable transmission of the *Dcl2b* mutations was not observed in WPT384-2 and WPT384-3 T_0_ plants

**Figure S24** Combining *dcl1a* and *dcl4b* mutations

**Figure S25** The *dcl1a^Δ6^/dcl1a^Δ6^/dcl4b^Δ2^/dcl4b^Δ2^* and wild-type plant

**Figure S26** *Gmdrb2a^Δ7^/drb2a^Δ1^*/*drb2b^Δ7^/drb2b^Δ4^* cross to wild type to recover single *drb2a* and *drb2b* mutant plants

**Figure S27** Combining the *Gmdrb2a^Δ7^/drb2a^Δ1^*/*drb2b^Δ7^/drb2b^Δ4^* and *Gmdcl1b^Δ15^* mutant alleles

**Figure S28** Combining the *Gmdrb2a^Δ7^/drb2a^Δ1^*/*drb2b^Δ7^/drb2b^Δ4^* and *Gmdcl4b^Δ2^* mutant alleles

**Figure S29** Combining the *Gmdrb2a^Δ7^/drb2a^Δ1^*/*drb2b^Δ7^/drb2b^Δ4^* and *Gmdcl1a^Δ7^* mutant alleles

**Figure S30** Combining the *Gmdcl4b^Δ2^* and *Gmdcl1a^Δ7^* mutant alleles

**Figure S31.** USDA-APHIS confirmation that *Glycine max* (soybean) line WPT590-4-28-5 is not a regulated article

**Figure S32**The identification of Cas9 over-expression cassettes in whole soybean and *M. truncatula* plants

**Supplemental Method1** TALEN design and assembly

**Supplemental Method2** Soybean genetic hybridization assay

**Supplemental Method3** Heteroduplex assay for detection of targeted mutations

**Supplemental Method4** Identifying transgene junctions and CRIPSR deletions

**Supplemental Method5** Phenotypic characterization of mutants

**Table S1** Analysis of WGS reads form WPT590-1, WPT590-2 and WPT590-4

**Table S2** Analysis of WGS reads form WPT527-1 and WPT527-2

**Table S3** List of TAL-effect nuclease RVD binding arrays and gene targets

**Table S4** List of CRISPR gRNA sequences and gene targets

**Table S5** Primers used in this study

**Table S6** Potential off-target sites generated for *Dcl3a*, *Drb2a* and *Drb2b* using CRISPR-P

**Table S7** List of reagents and their targets in Medicago and soybean


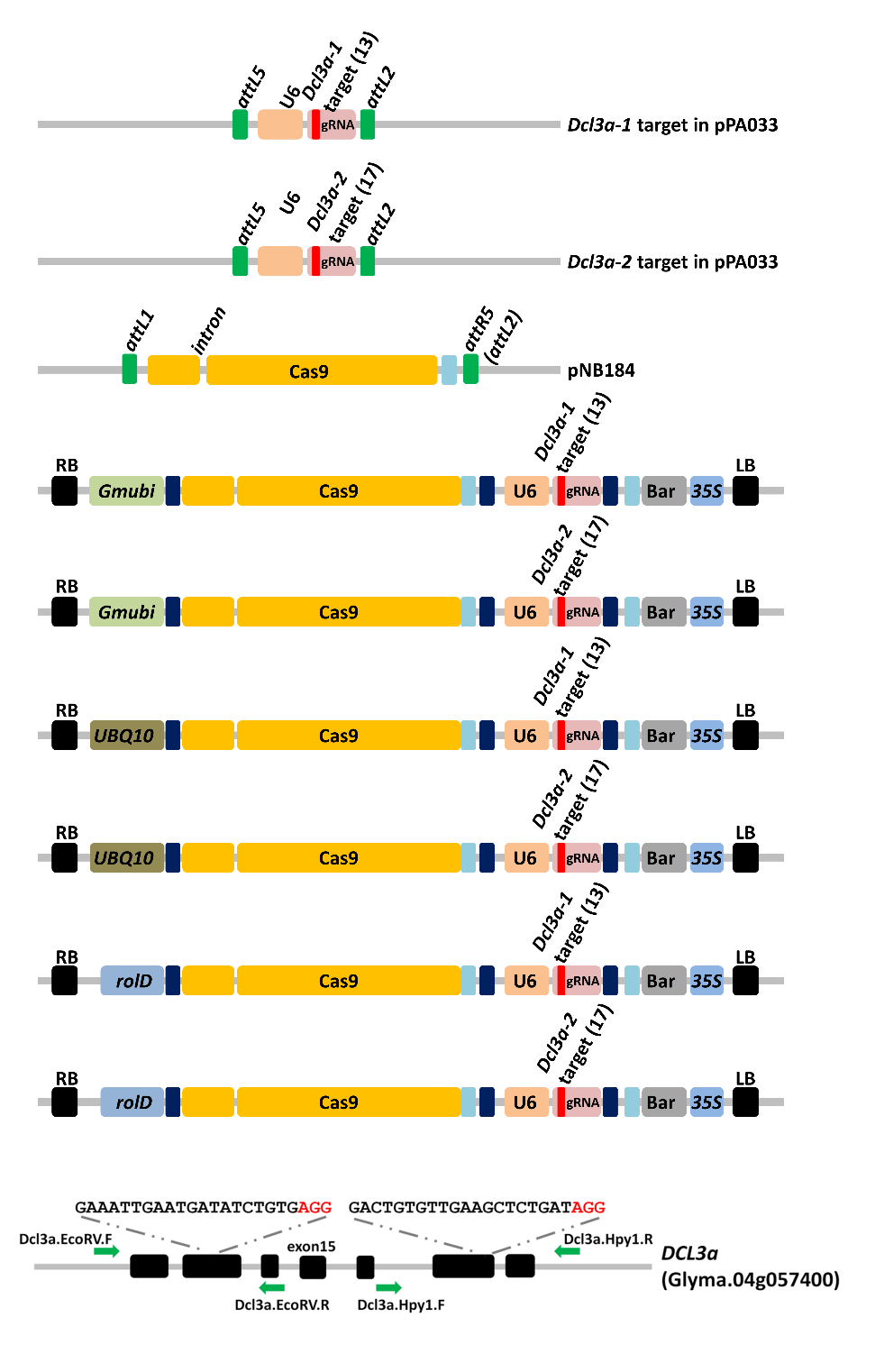
a

b

**Figure S1**. (a) Schematic representations of the CRISPR/Cas9 reagent vectors targeting *GmDcl3a* used to test vector function in hairy-root tissue. The first vector utilizes the constitutively expressed *Glycine max* ubiquitin promoter (*Gmubi*) to drive Cas9 (pSC218G). The second vector uses the *Arabidopsis* Ubiquitin10 promoter selected for its reputed expression in germ-line tissues (pSC218U). The third vector uses the *A. rhizogenes* *rol*D promoter reported to be highly expressed in regenerating tissue and in callus (pSC218R). (b) A schematic representation of the two gRNA targets in exon 13 and 17 of the *GmDcl3a* gene (Glyma.04g057400). The red sequence represents the PAM and the green arrows represent primers used to screen mutant hairy roots.


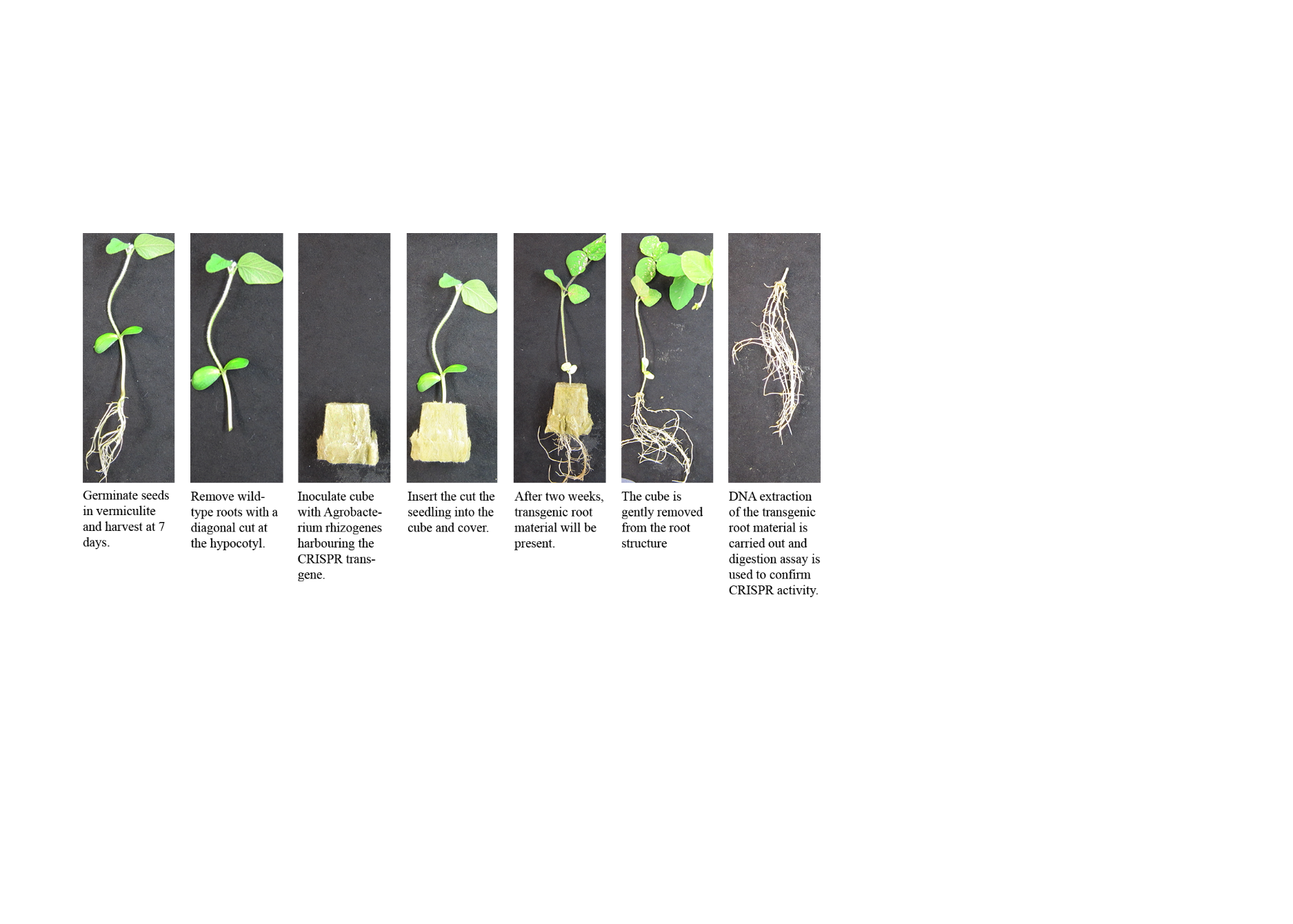
a


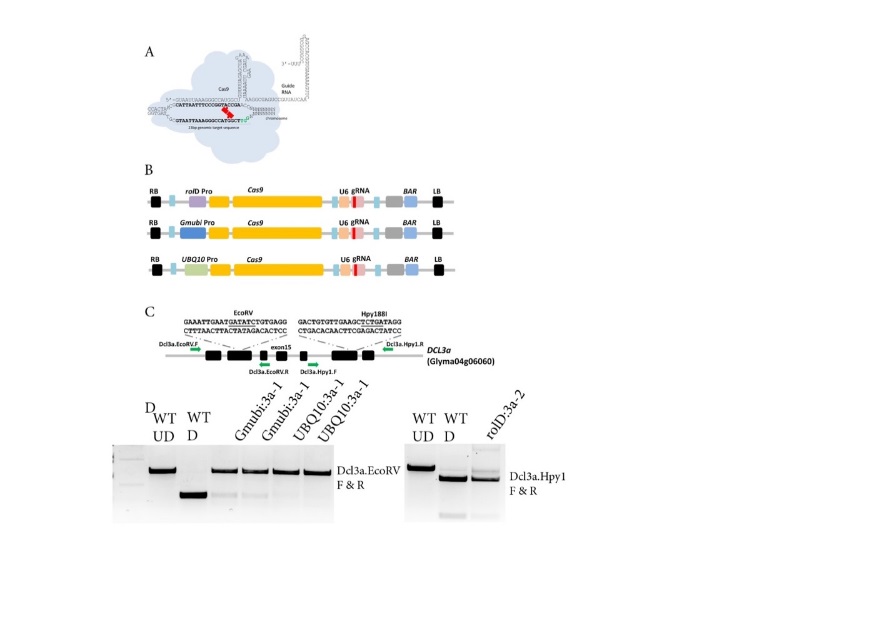
b


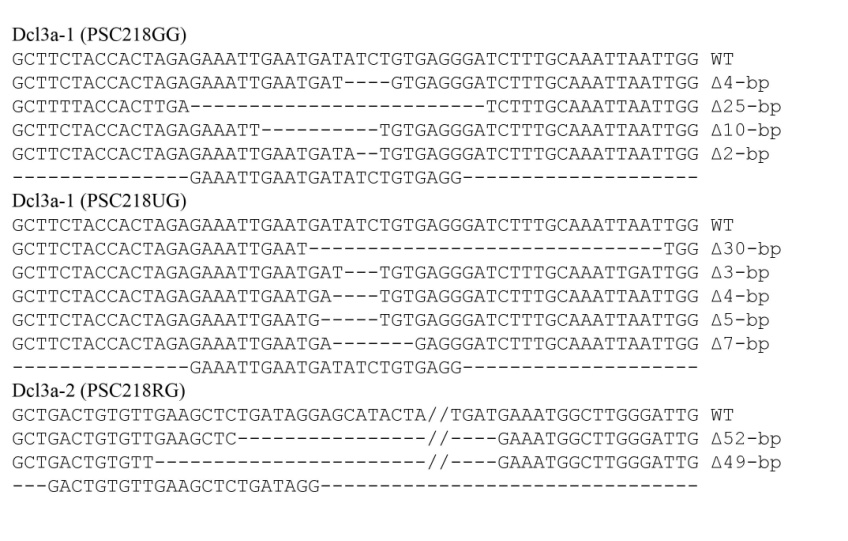
c

**Figure S2**. (a) The *ex vitro* hairy-root transformation assay developed by Taylor et al. 2006 was used to rapidly introduce transgenes into somatic soybean tissue and test for site-directed mutagenesis (b) A PCR assay using restriction enzyme digested enriched template from transformed hairy-root tissue was separated by gel electrophoresis. PCR digest resistant bands identical to undigested wild-type control (the lanes labeled as 'WT UD’) is indicative of mutated sequence. **(c)** Sequence confirmation of mutated target amplicons demonstrate the correct function of each expression vector.


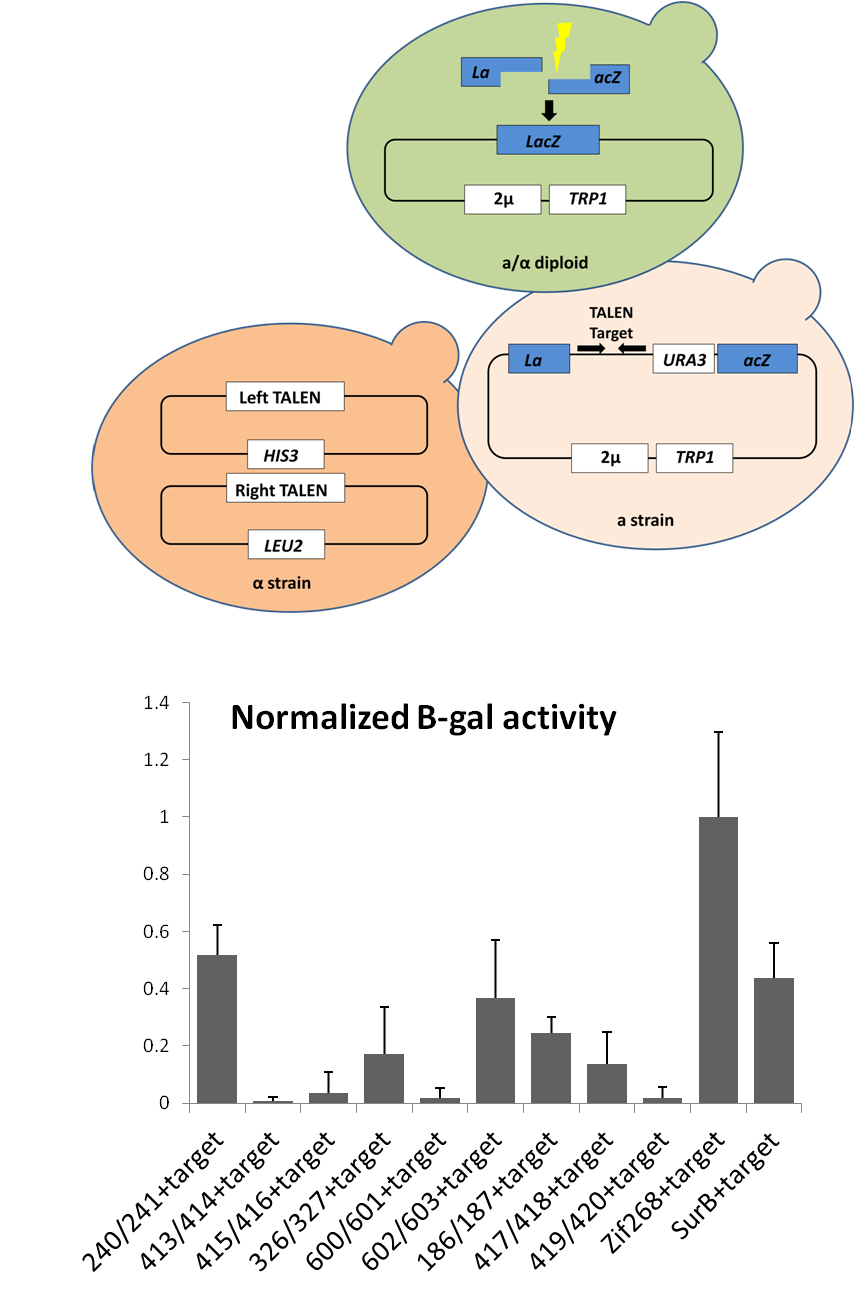
a

b

**Figure S3**. (a) A representation of the yeast assay used to screen TALEN function prior to transformation into whole plant soybean. A haploid yeast strain expressing a TALEN pair is mated with a second haploid yeast strain carrying the corresponding target plasmid. A functional TALEN pair will cleave the target in diploid yeast and restore function to the LacZ gene. The levels of β-galactosidase activity correlate with reagent cleavage activity. (b) The nuclease activity of the nine TALEN pairs were tested with β-galactosidase levels normalized to the Zif268 zinc finger array (Christian *et al.*, 2010). Three candidate TALEN pairs with significant cleavage activity were identified *Dcl2a*^240/241^, *Dcl2b*^602/603^ and *Dcl3a*^186/187^.


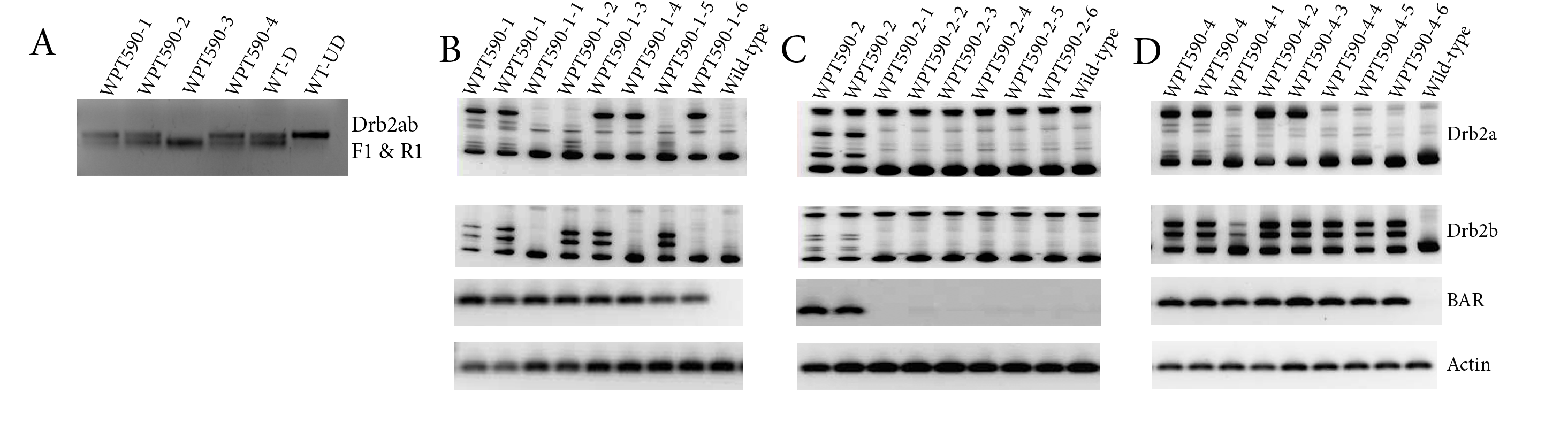


a


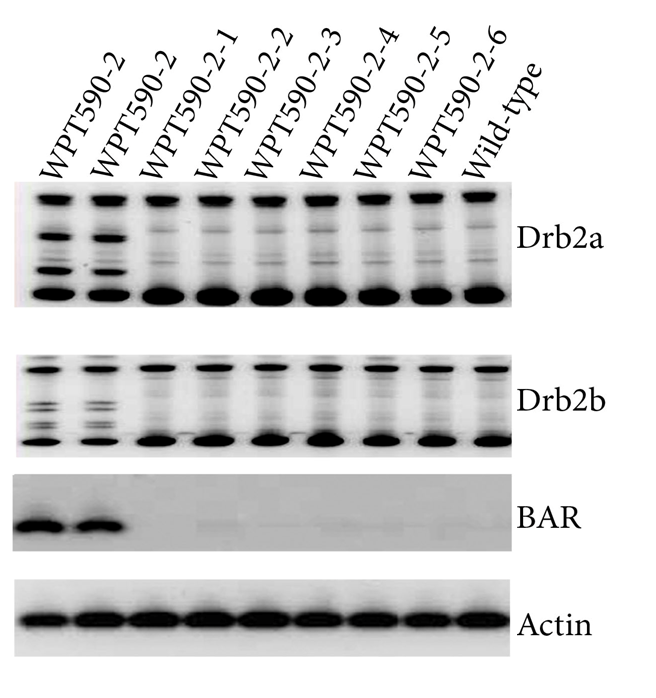

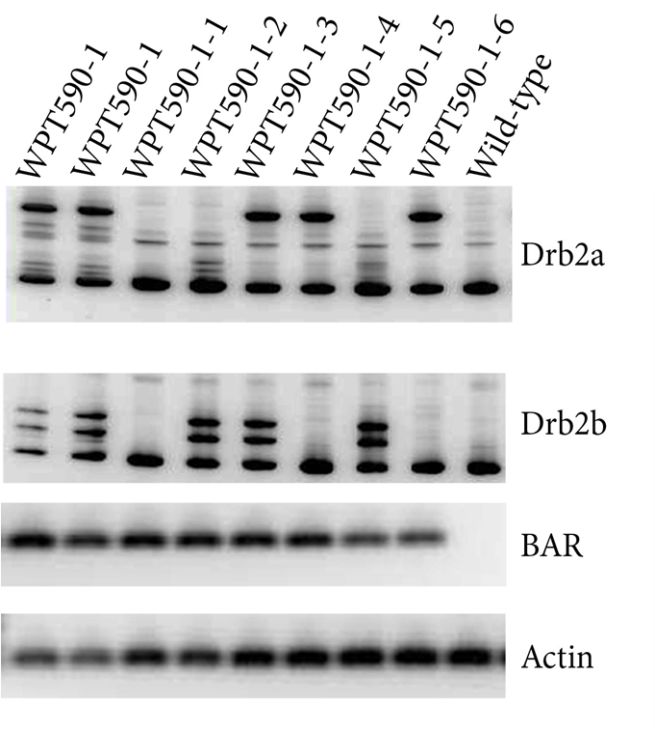


b c


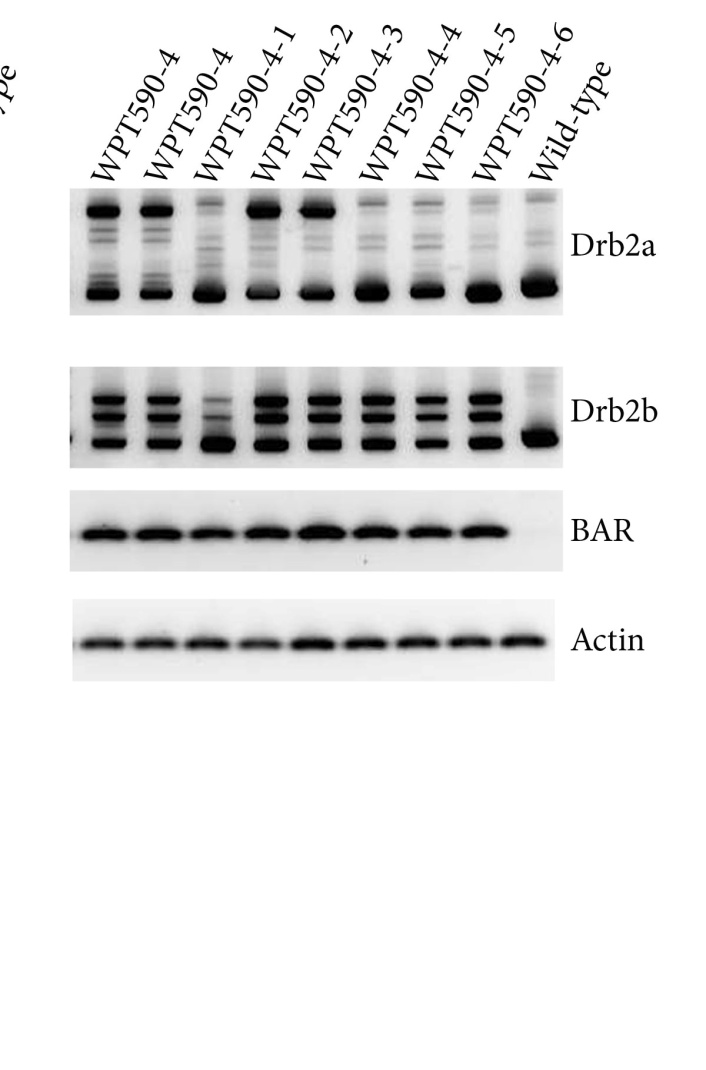


d

**Figure S4**. PCR-digest (a) and hetero-duplex (b-d) gel analysis of WPT590-1, WPT590-2, WPT590-3 and WPT590-4 T_0_ and T_1_ soybean plants. The PCR digest shows evidence for mutations (the presence of upper bands) in WPT590-1, WPT590-2, and WPT590-4, but not WPT590-3. The heteroduplex data (b-d) shows evidence for the presence of heterozygous mutations (indicated by unique banding patterns) in some T_1_ progeny of WPT590-1 and WPT590-4, but not WPT590-2.

**
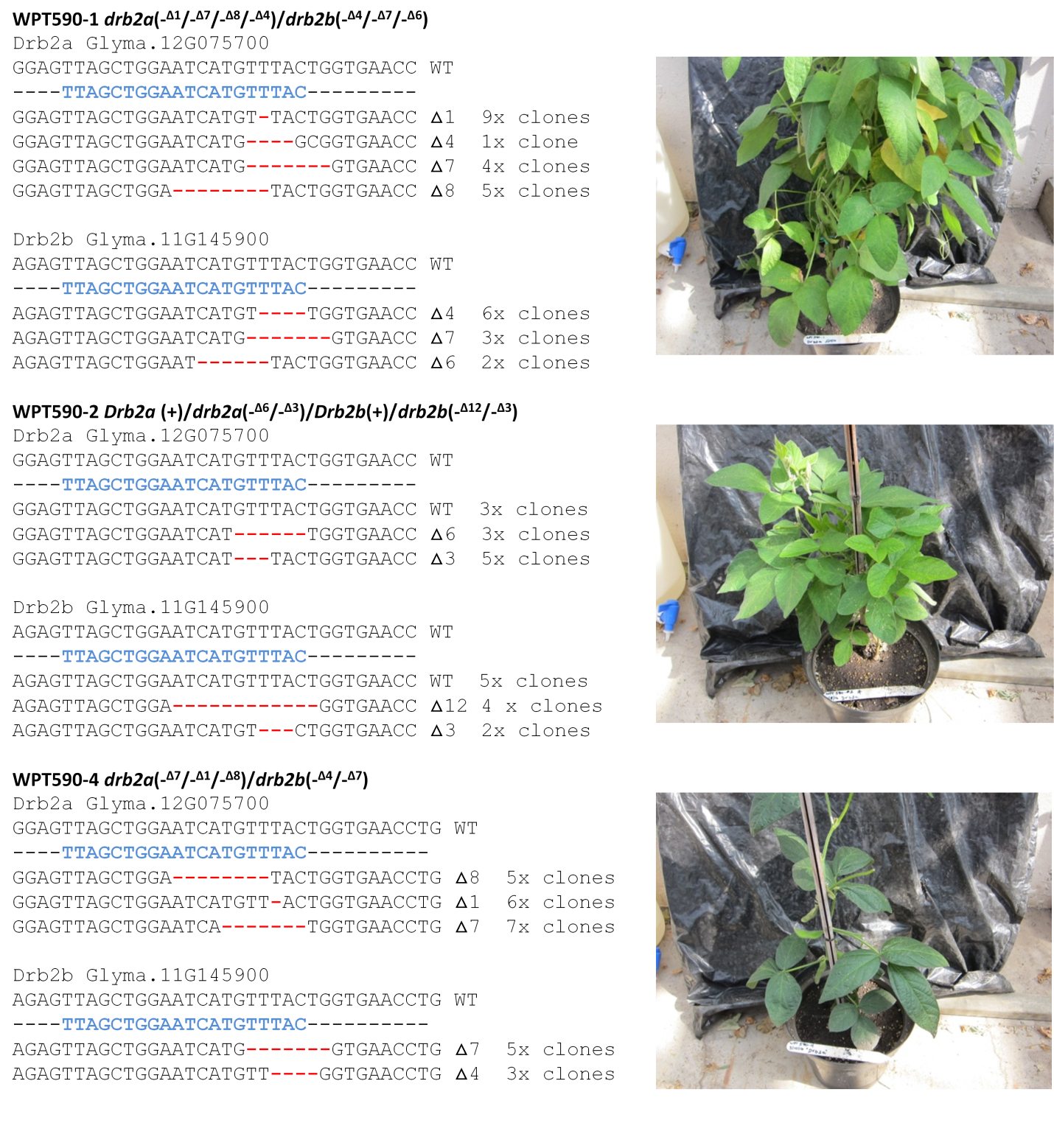
**

**Figure S5**. A target amplicon clone and sequence (TACAS) assay was used to validate mutations in the WPT590-1, WPT590-2 and WPT590-4 plants. WPT590-3 was also screened but no evidence of mutation was observed.


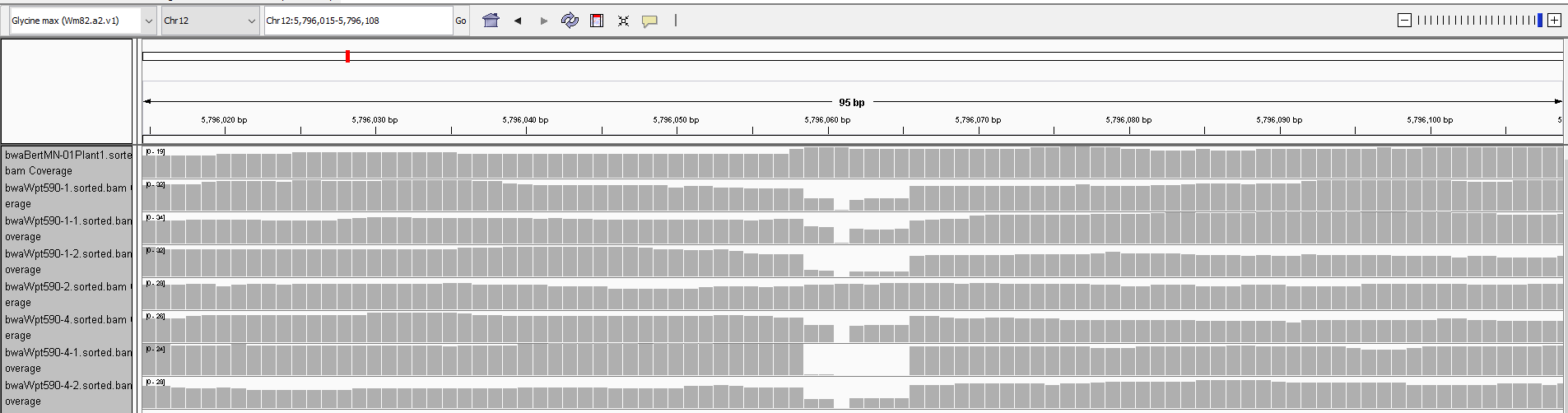

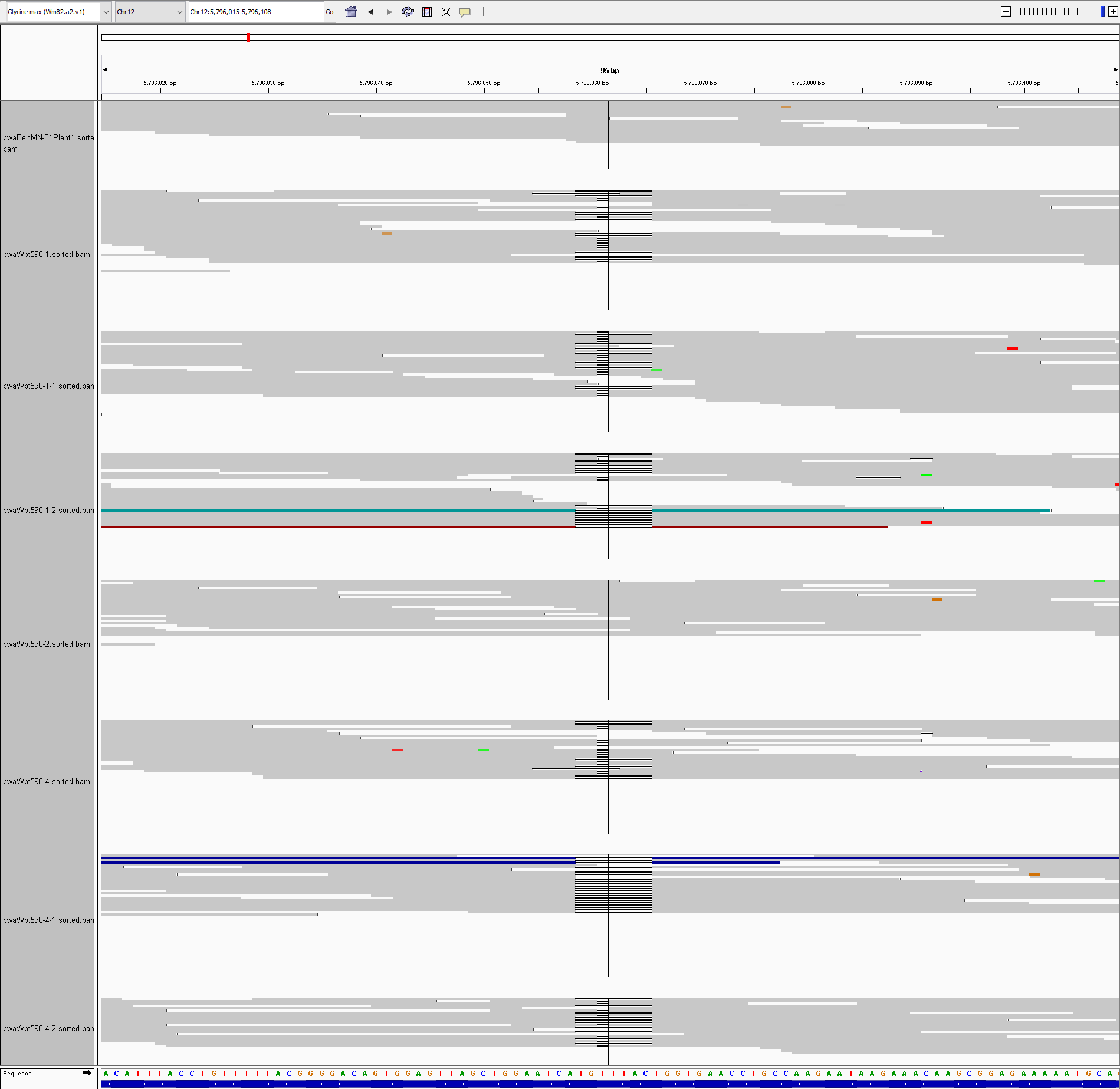


**Figure S6**. Whole genome sequence analysis of *Drb2a* (Glyma.12g075700) target sites in Bert, WPT590-1, WPT590-1-1, WPT590-1-2, WPT590-2, WPT590-4, WPT590-4-1 and WPT590-4-2 plants.


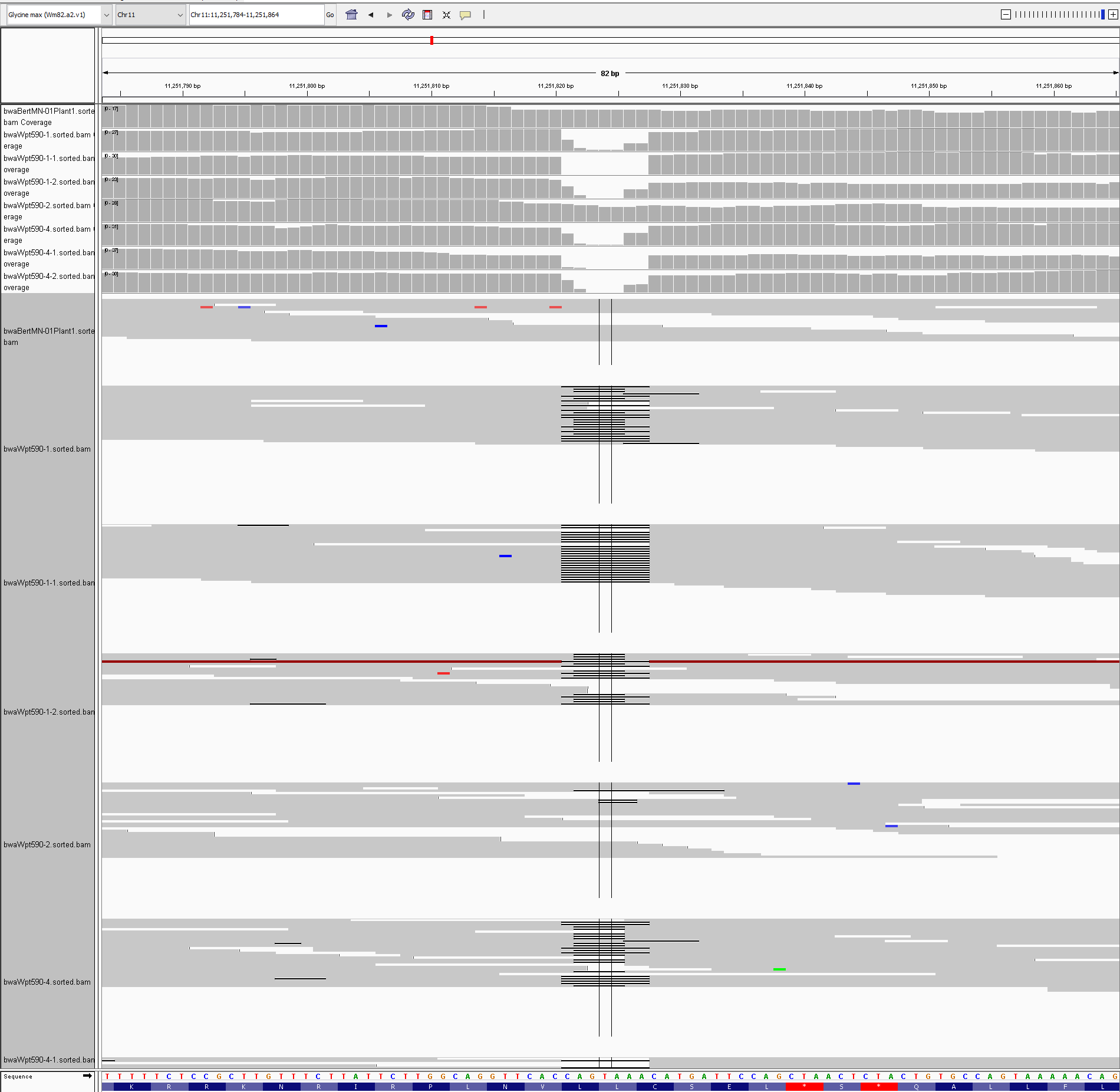

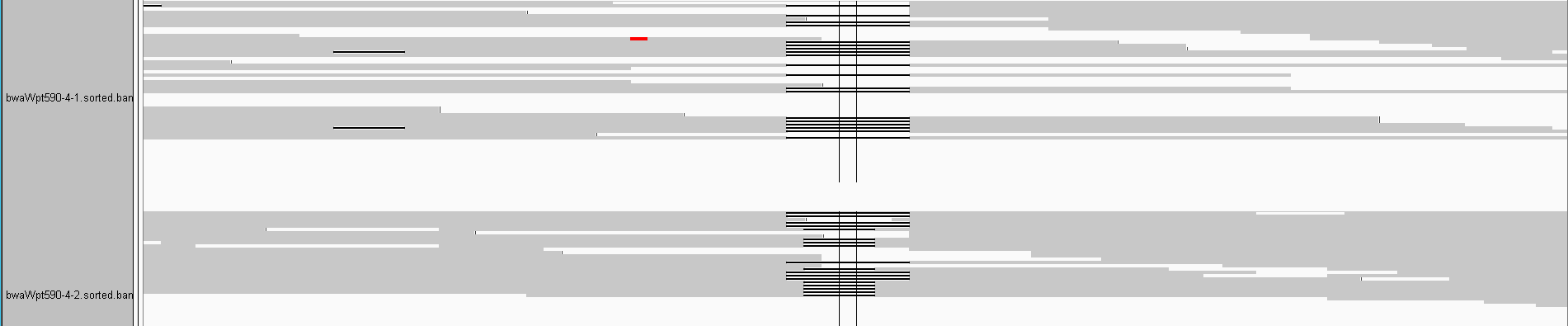


**Figure S7**. Whole genome sequence analysis of Drb2b (Glyma.11g145900) target sites in Bert, WPT590-1, WPT590-1-1, WPT590-1-2, WPT590-2, WPT590-4, WPT590-4-1 and WPT590-4-2 plants.


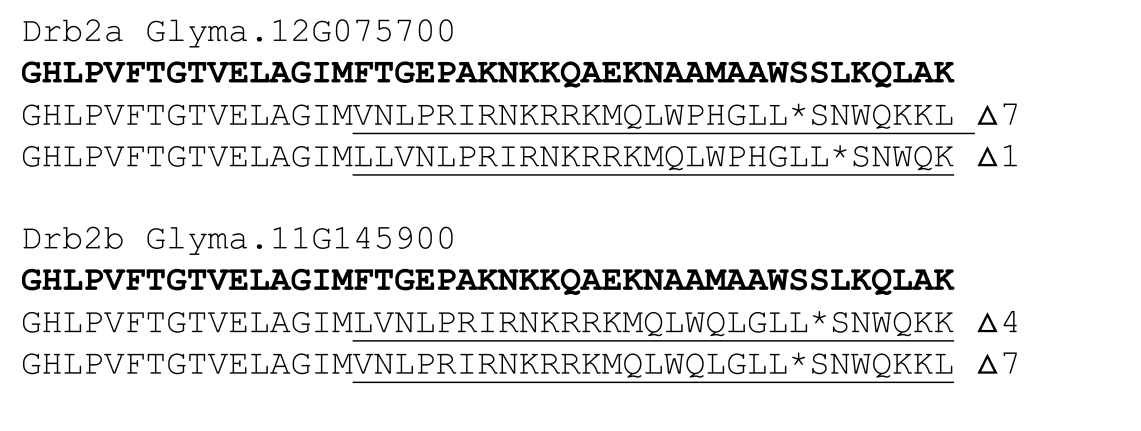


**Figure S8**. The target site encodes the start of the highly conserved second double-stranded RNA-binding motif (dsRBM) of the protein that results in a frame-shift mutation and premature stop codon in all four mutated alleles.


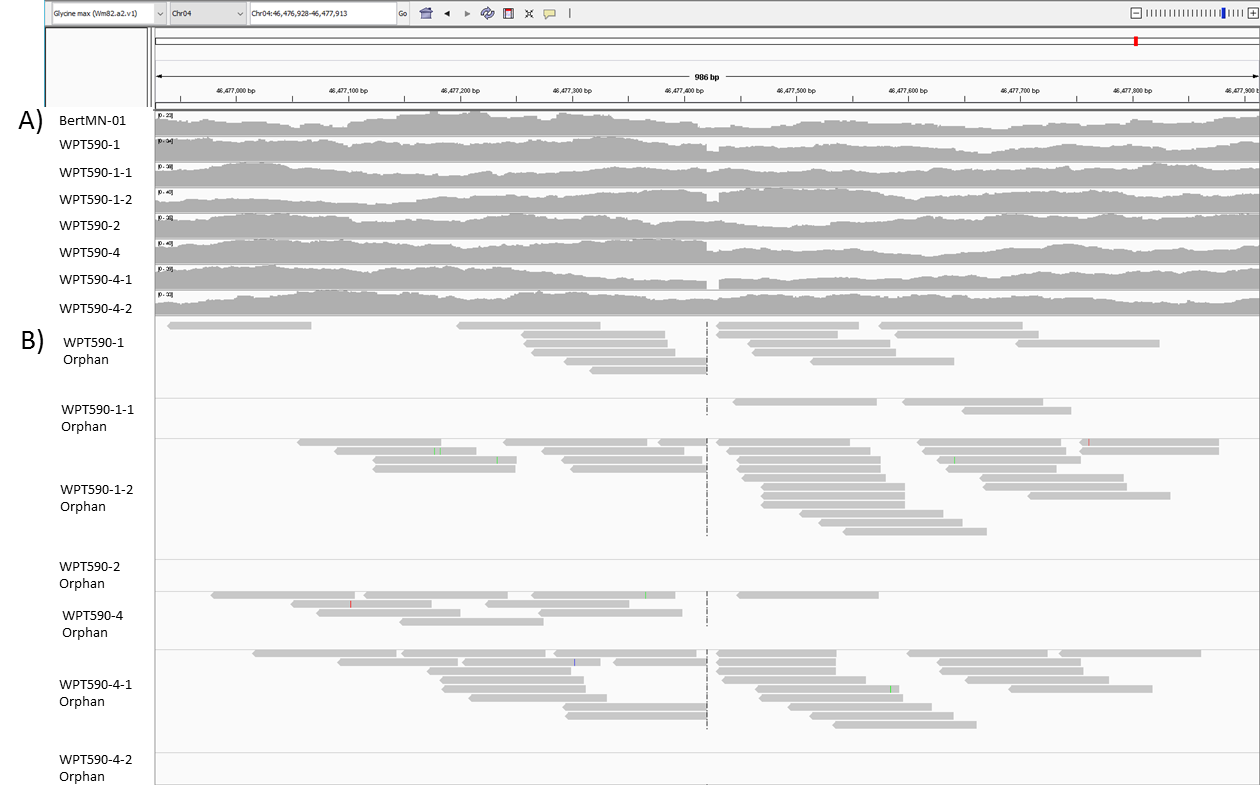


**Figure S9**. Coverage of reads mapping to the reference genome surrounding the transgene insertion site at chromosome 4 in WPT590-1 and WPT590-4. (Data from WPT590-2 is also shown, but there is no transgene insertion at this site in that line.) In the upper panels, taller grey peaks represent greater sequence coverage, while abrupt dips in coverage may represent homozygous or heterozygous deletions. Transgene segregation can be inferred from this data. For example, the reads in WPT590-4-1 indicate an abrupt dip to the baseline in the middle of the screen, indicating a potential homozygous deletion (presumably accompanied by a homozygous transgene insertion at this site). Whereas, there is no dip at this site in WPT590-4-2, indicating no deletion (and presumably no transgene insertion at this site). In the lower panels, the horizontal grey bars represent extracted orphan reads that were mapped back to the reference genome.

**
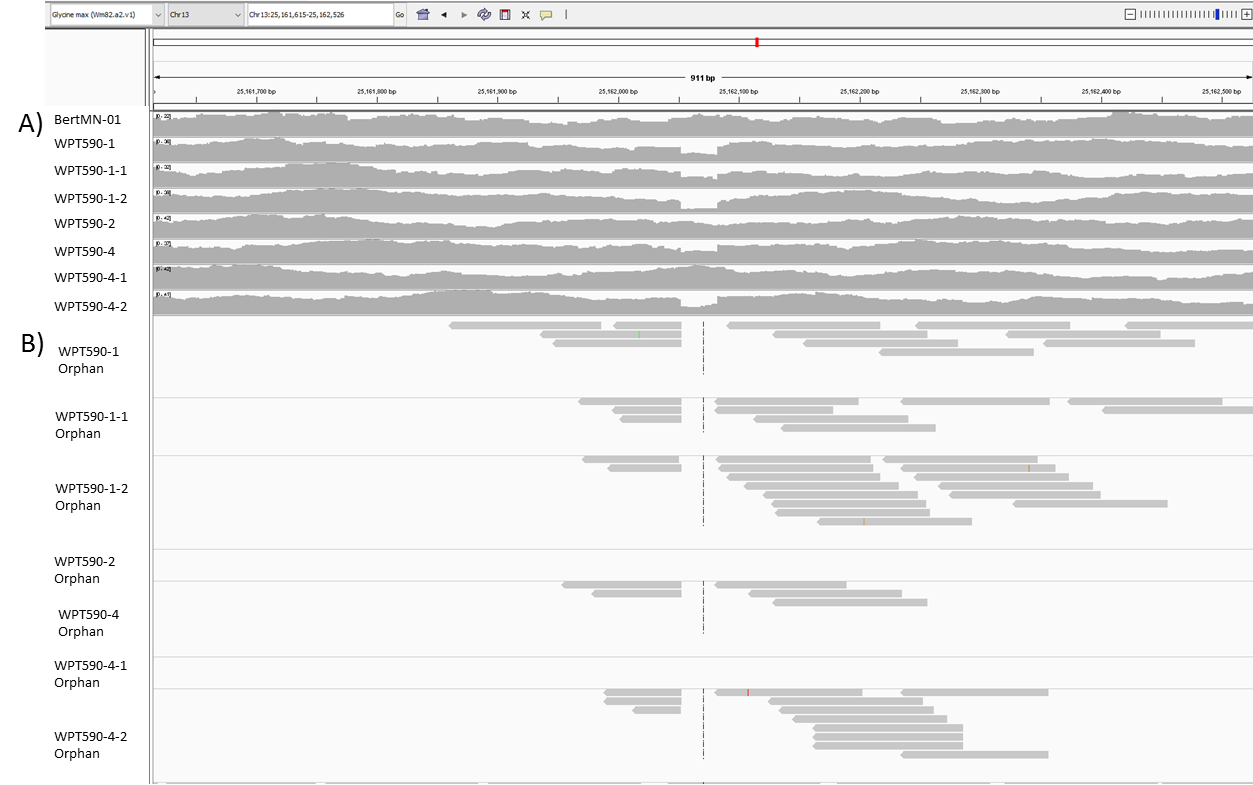
**

**Figure S10**. Coverage of reads mapping to the reference genome surrounding transgene insertion sites at chromosome 13. In the upper panels, taller grey peaks represent greater sequence coverage, while abrupt dips in coverage may represent homozygous or heterozygous deletions. Transgene segregation can be inferred from this data.


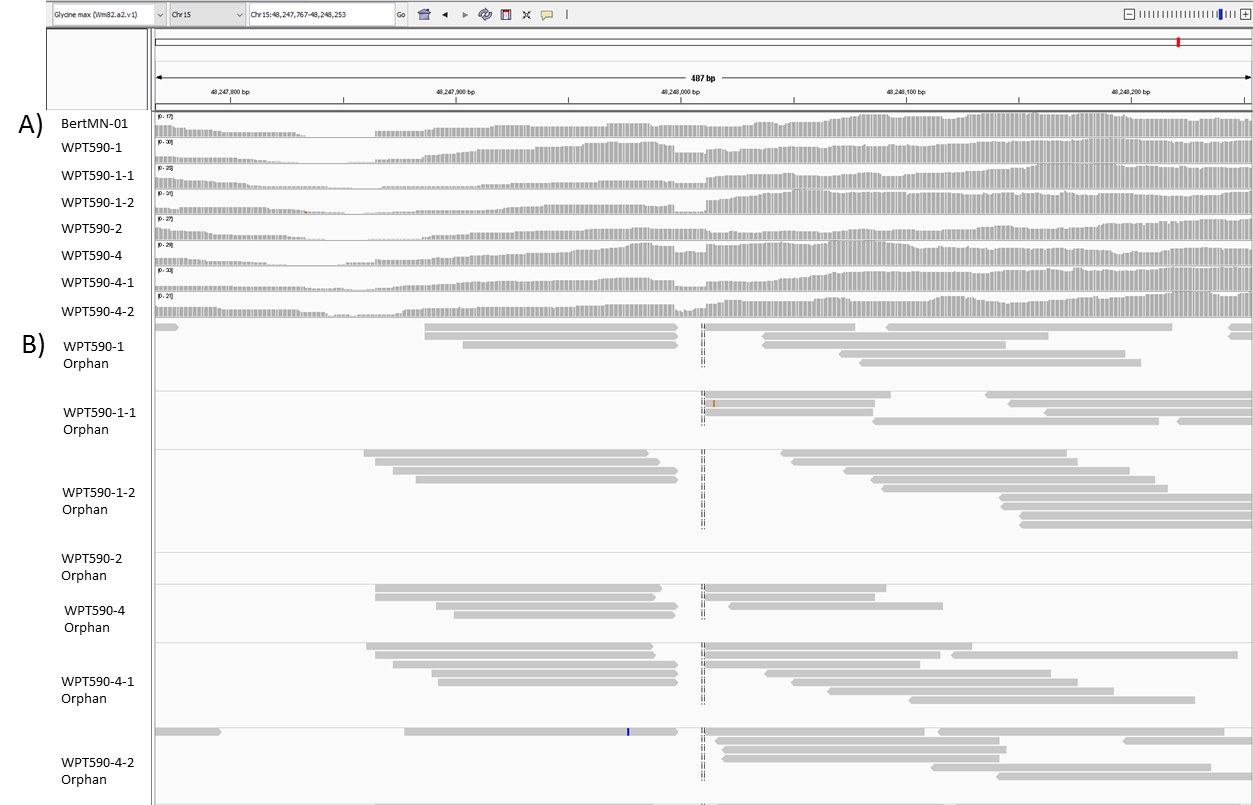


**Figure S11**. Coverage of reads mapping to the reference genome surrounding transgene insertion sites at chromosome 15. In the upper panels, taller grey peaks represent greater sequence coverage, while abrupt dips in coverage may represent homozygous or heterozygous deletions. Transgene segregation can be inferred from this data.


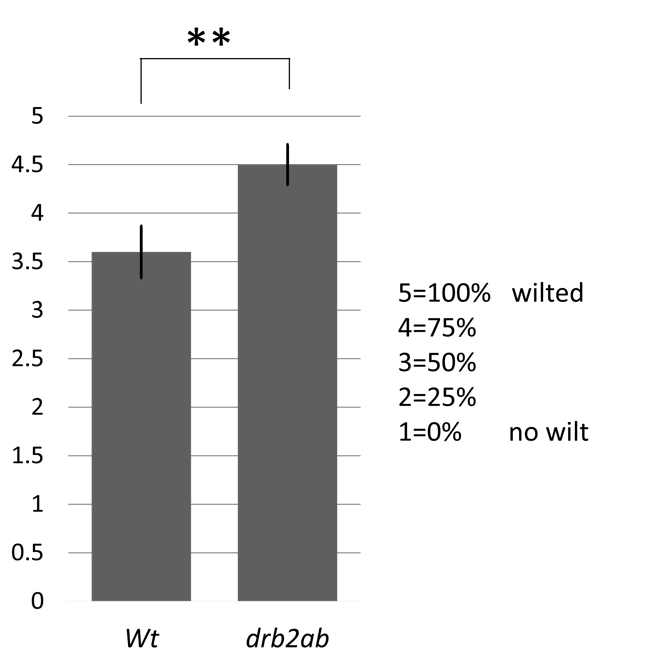


a

b

**
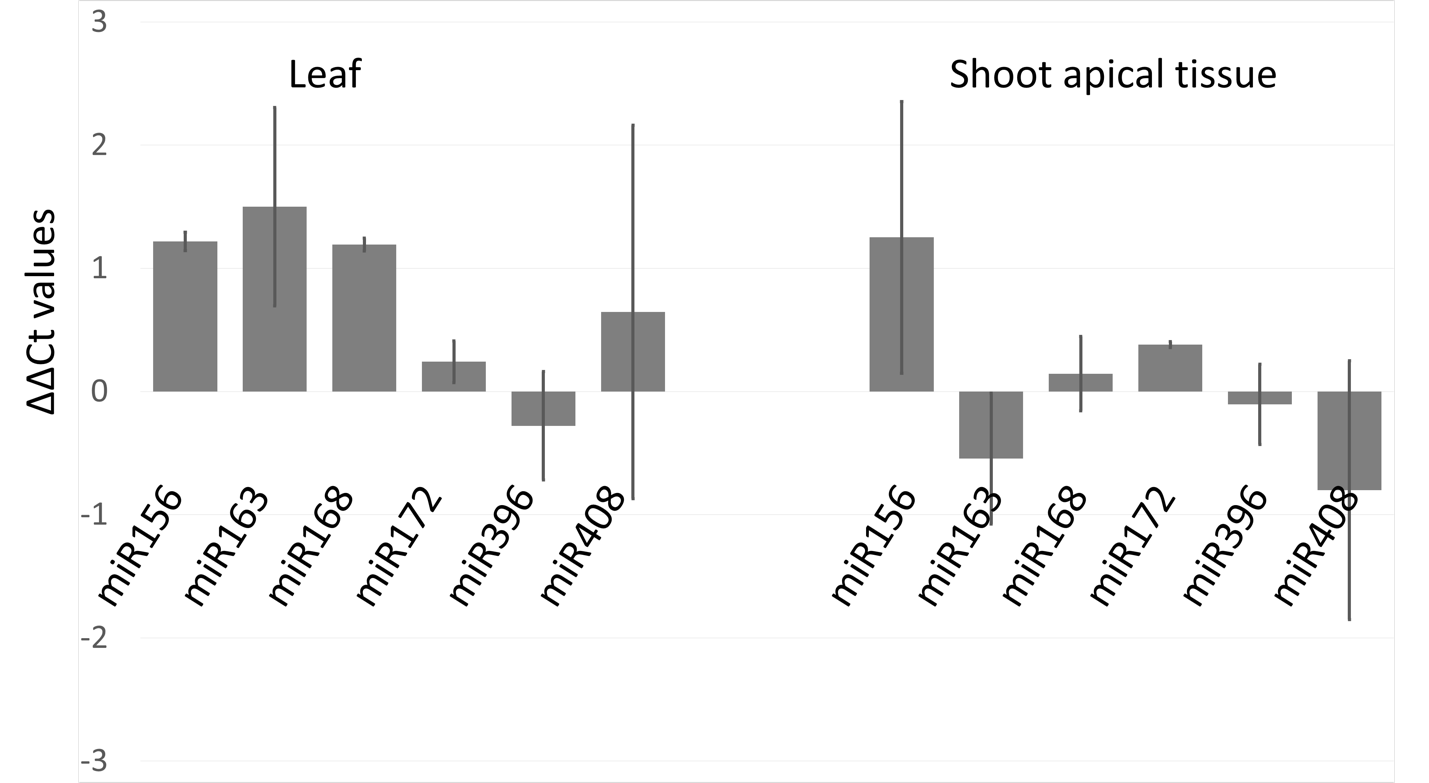
**

**
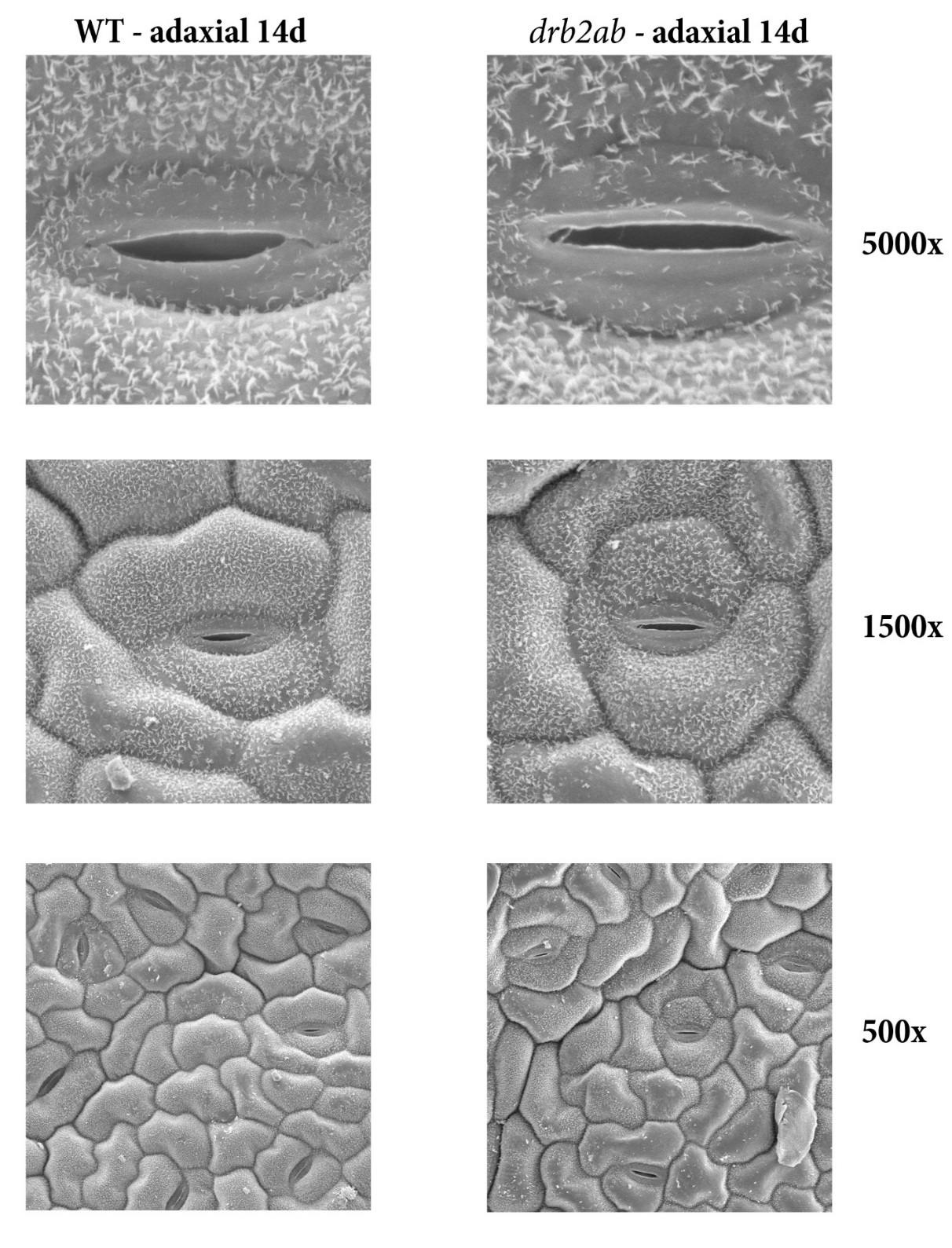
c**

**Figure S12** Phenotype analysis of the *drb2ab* double mutant (a) A preliminary phenotype analysis was carried out on 24 mutant and 24 ‘Bert’ wild-type 14 day old seedlings. Statistically significant sensitivity to drought stress (p=0.01) was observed in the mutant plants. (b) Stem-loop quantitative reverse transcription PCR (SL-qPCR) was used to compare the expression of six miRNAs in mutant and wild-type plants (c) SEM analysis of *drb2ab* mutant on 14 day old adaxial leaf surfaces.


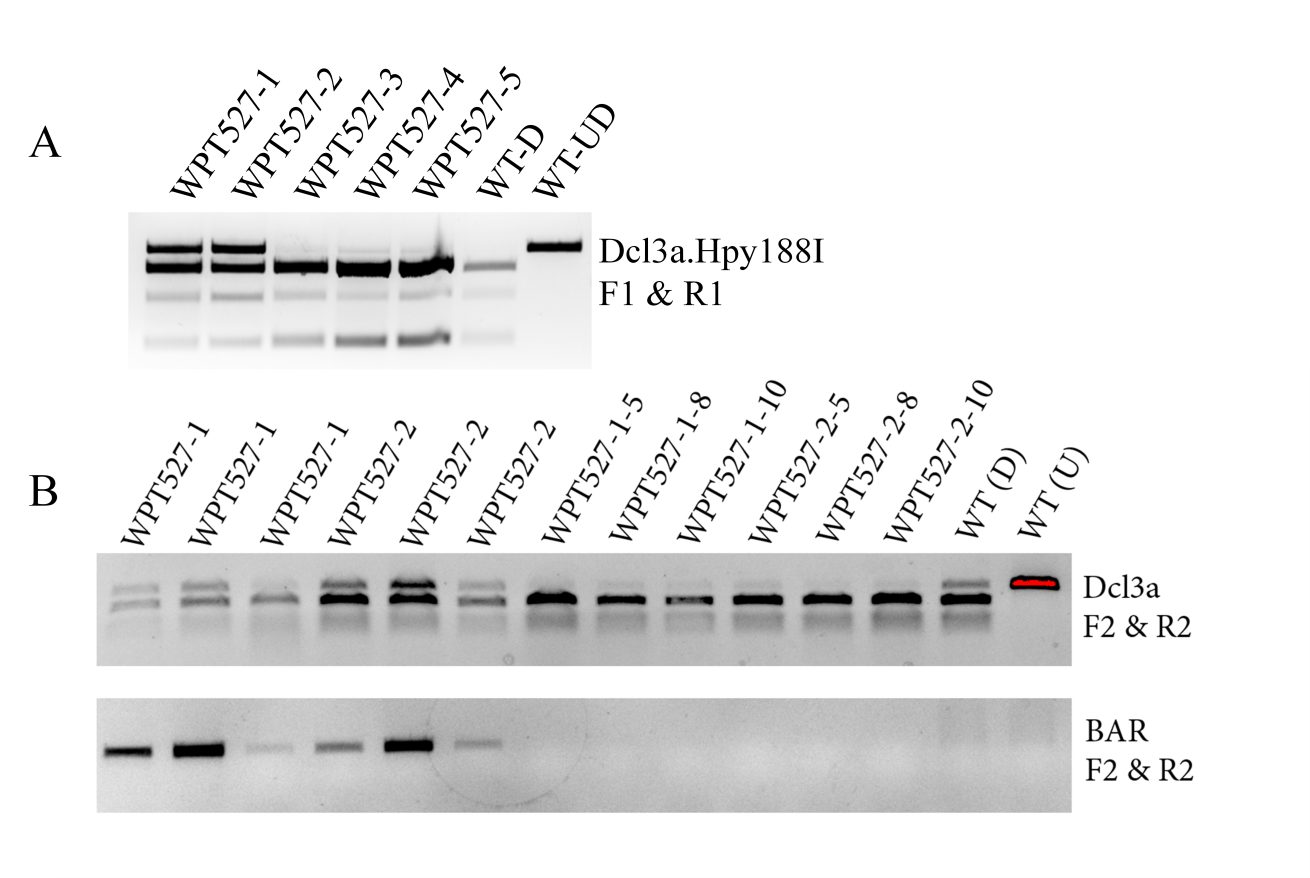


a

b

**Figure S13** (a) A PCR-digestion assay on DNA template using primers spanning the *GmDcl3a* target site in the WPT527 series. Hpy188I resistant amplicons similar to the un-digested wild-type amplicons indicate mutated target sequence. T_0_ plants WPT527-1 and WPT527-2 showed strong evidence for mutations. (b) A PCR-digestion assay of three leaf samples taken from different parts of the WPT527-1 and WPT527-2 T_0_ plants along with three T_1_ plants from each event. Approximately sixty T_1_ plants from both WPT527-1 and WPT527-2 were screened, with no evidence for heritable transmission observed in any of the progeny. In addition, plants were screened to detect for the presence of the transgene, however no transgene was observed in any T_1_ plants.


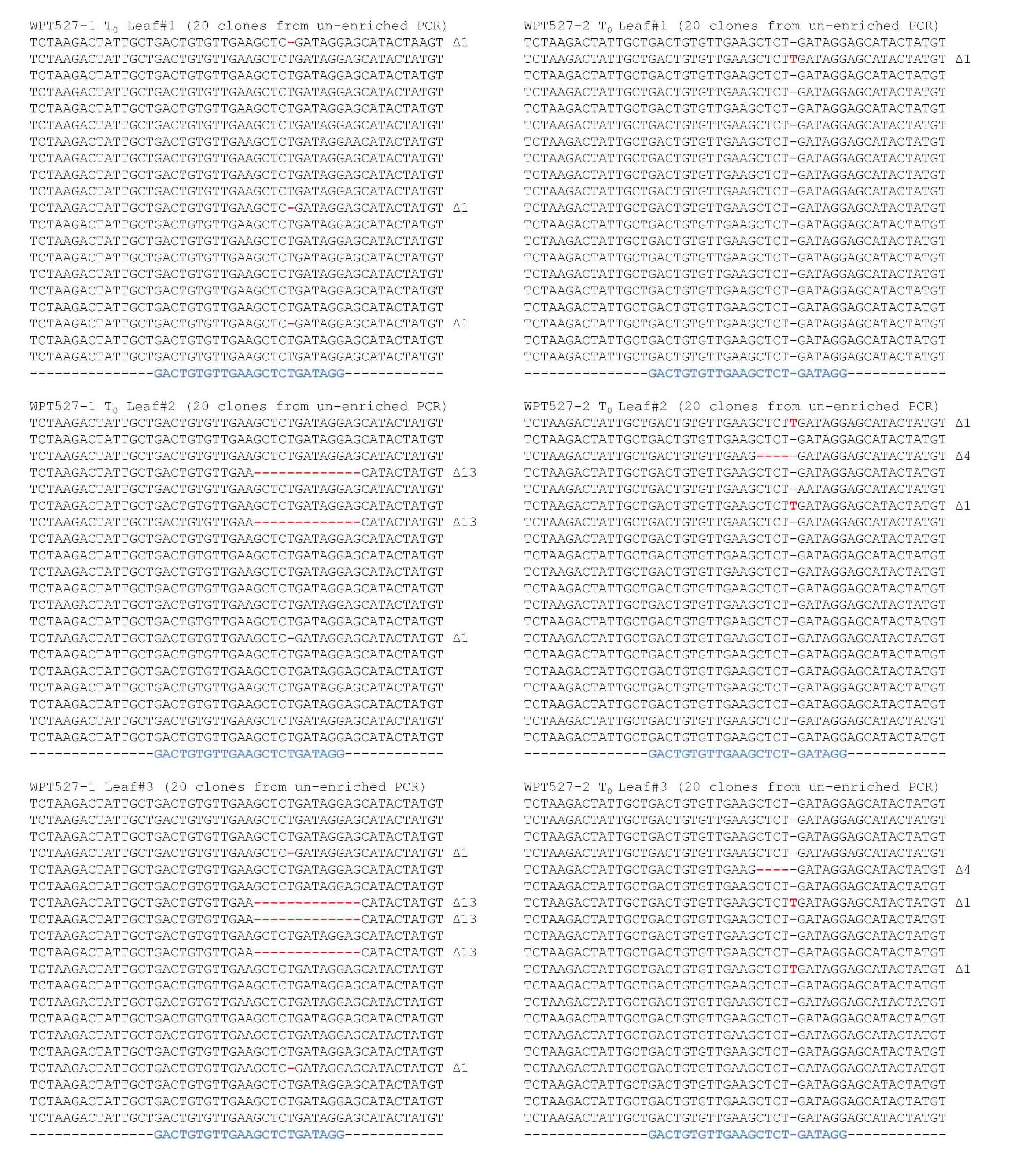


**Figure S14**. Sequence data from three leaf samples from WPT527-1 and WPT527-2 T_0_ plants. The proportion of mutated alleles to wild-type alleles suggests that neither plant was a bone fide heterozygous mutant. Curiously, the same mutations in each leaf sample for each event was observed. However, it is unclear whether these mutations traced back to the same cell, or if they were independently derived in different sectors of the plant.


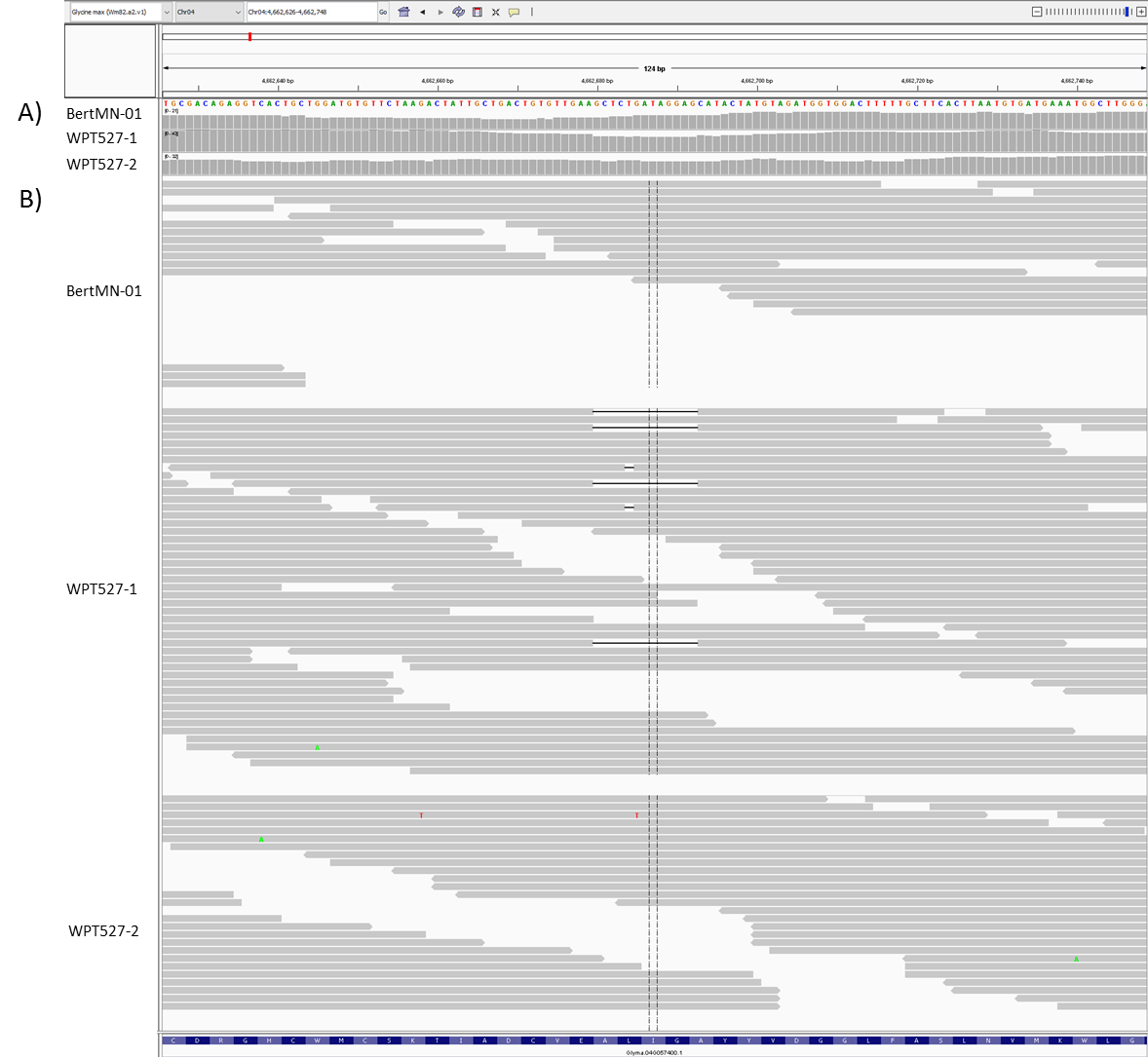


**Figure S15**. Base pair coverage and read mapping of the CRISPR target for Glyma.04g057400. In the upper panels, each vertical grey bar represent a single base; the taller the bar, the greater the sequence coverage. In the lower panels, horizontal grey bars represent reads that map to the genome. A black line splitting a read represents a deletion, in this case the 1-bp and 13-bp deletions observed in the TACAS assay.


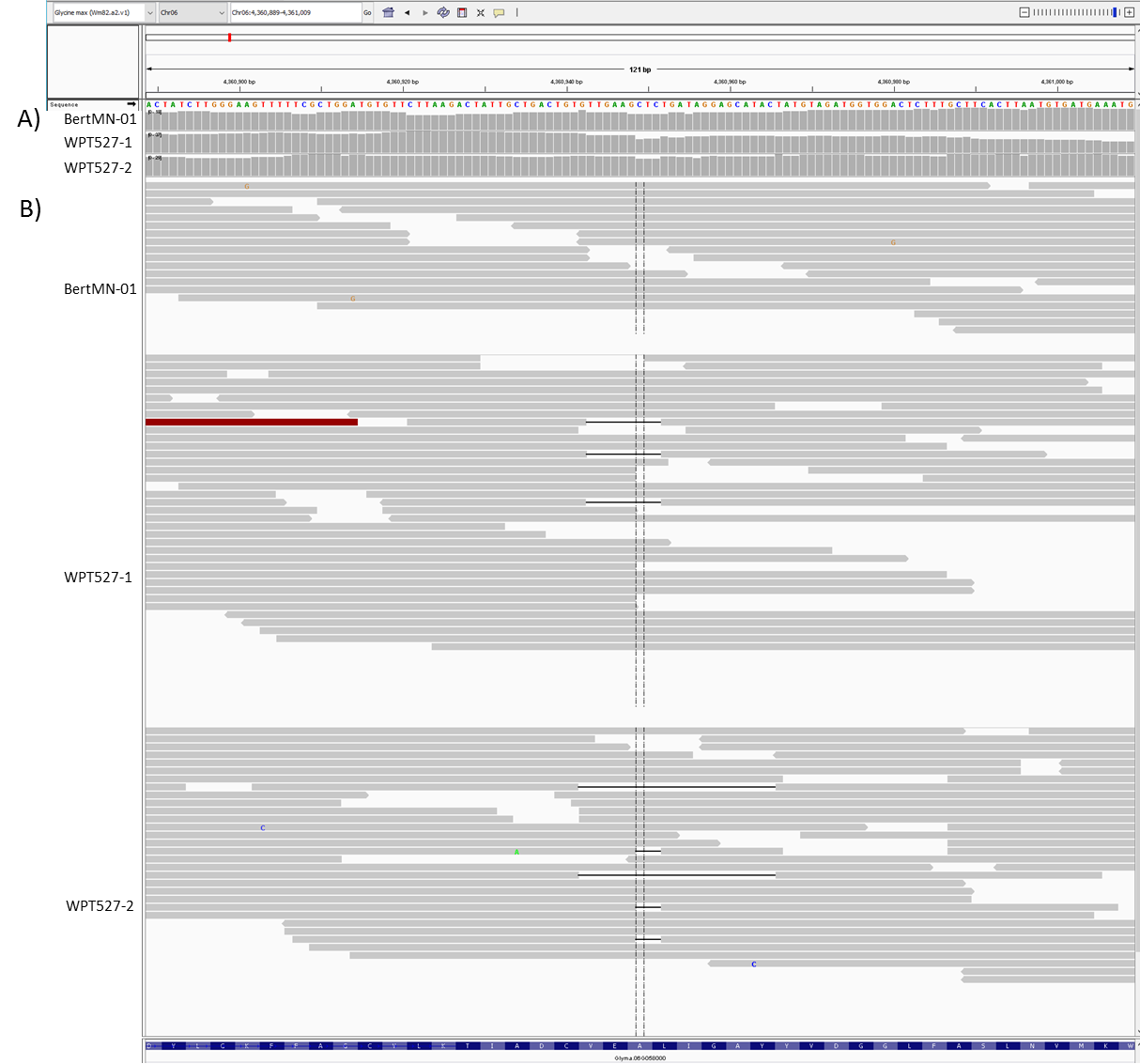


**Figure S16**. Base pair coverage and read mapping of the CRISPR target for the non-functional *Gmdcl3b* pseudo-gene (Glyma.06G05800) in WPT527-1 and WPT527-2 T_0_ plants. In the upper panels, each vertical grey bar represent a single base; the taller the bar, the greater the sequence coverage. In the lower panels, horizontal grey bars represent reads that map to the genome. A black line splitting a read represent a deletion.


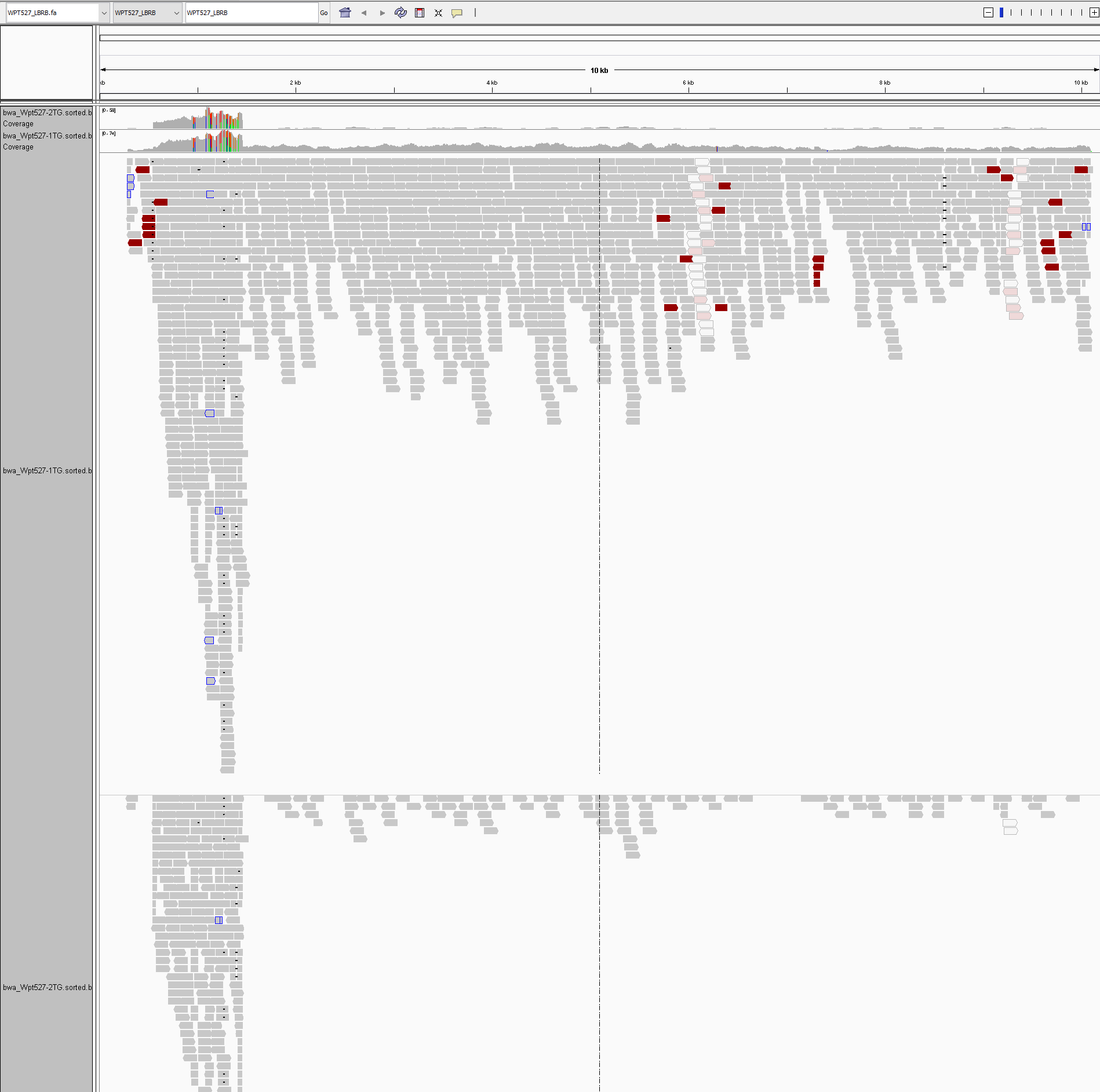


**Figure S17**. Reads that map to the *dcl3a* reagent transgene. WGS reads of WPT527-1 and WPT527-2 that map to left-border and right-border of the reagent transgene cassette.


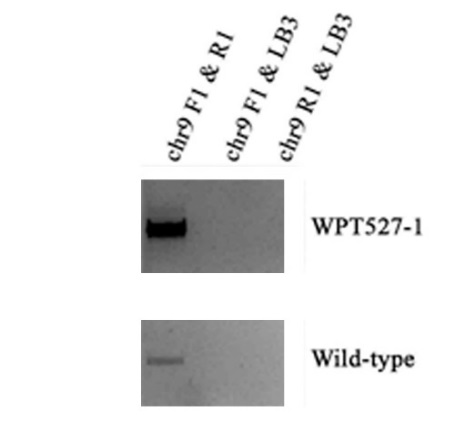


**Figure S18**. PCR primers were designed spanning the putative chromosome nine position of the reagent transgene identified in WPT527-1. Results from the PCR assay suggested that there is no transgene at this locus


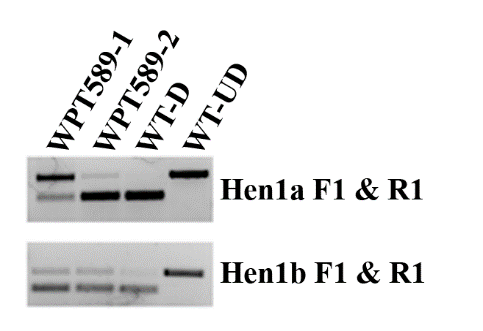
a


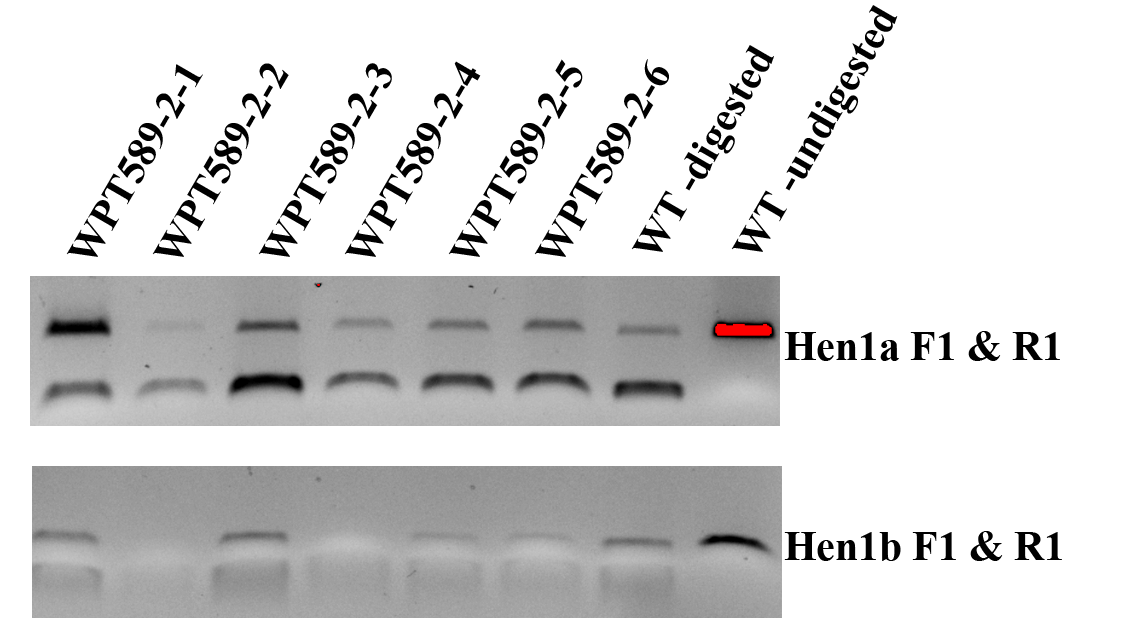


b


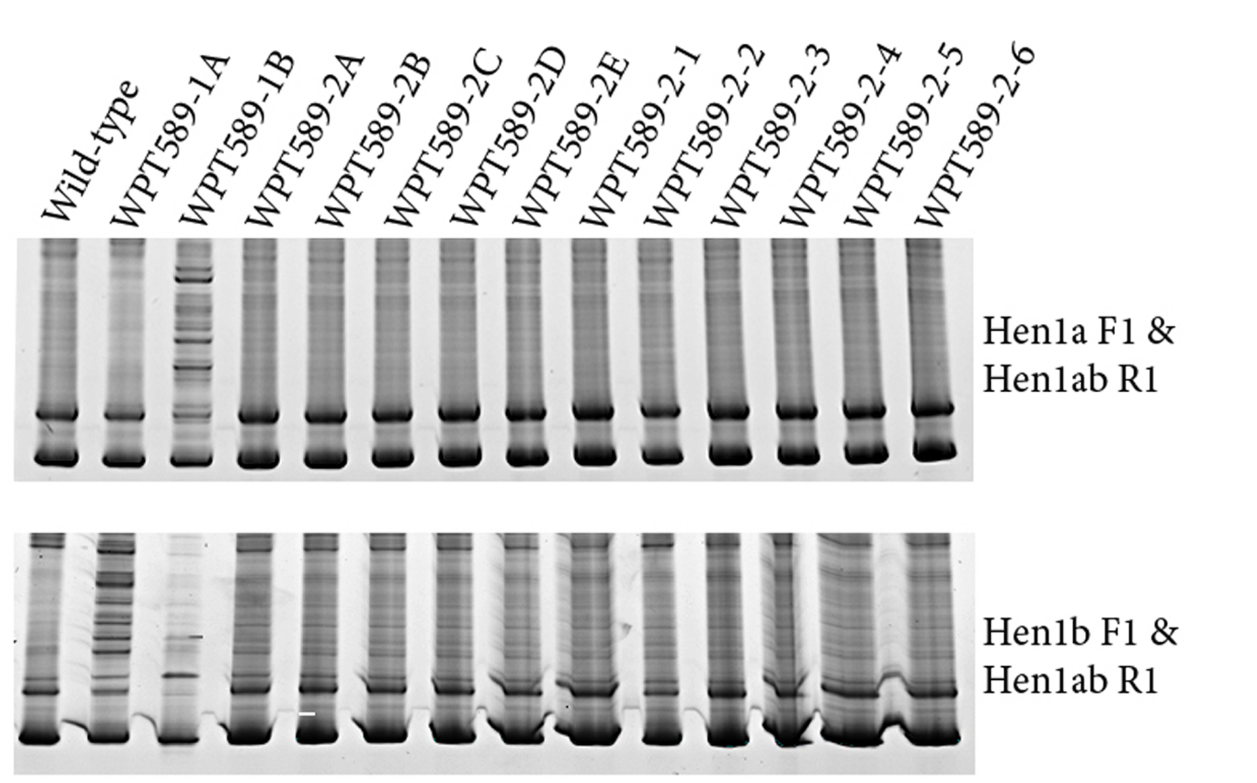


c


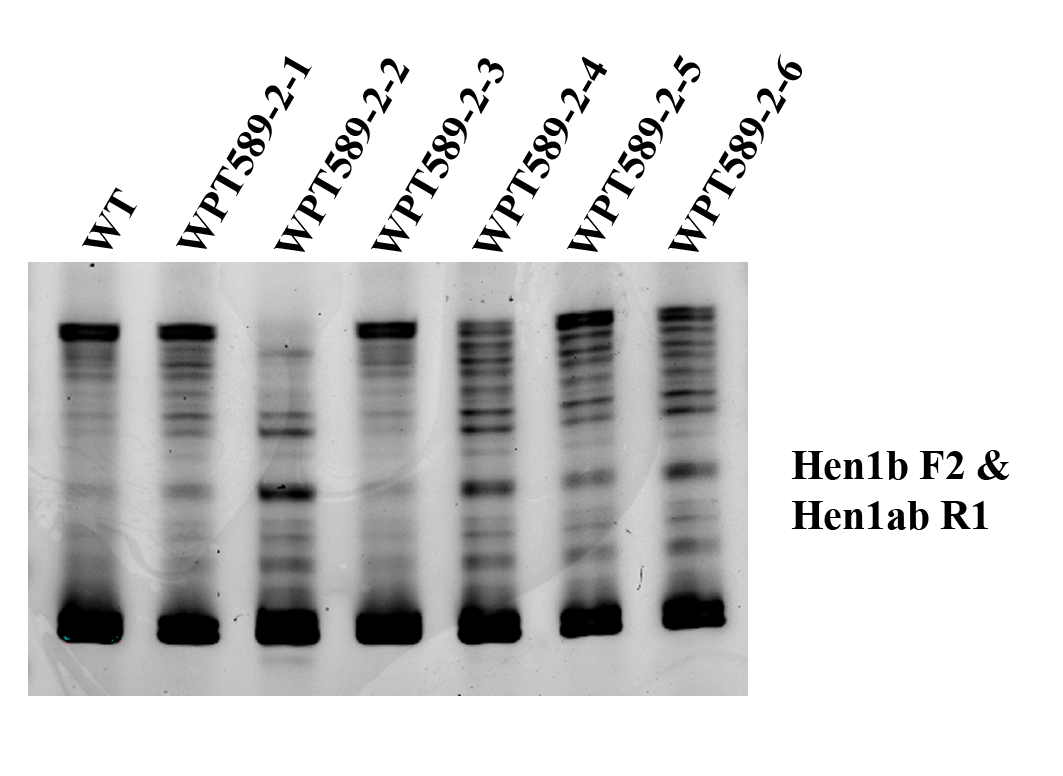


d

**Figure S19**. (a) PCR-digestion assay of *Hen1a* (Glyma.08g081600) and *Hen1b* (Glyma.05g126600) target amplicons from un-enriched DNA templates from WPT589-1 and WPT589-2 T_0_ plants. Digestion resistant bands indicate putative mutations; WPT589-1 exhibited strong evidence for mutation(s) and WPT589-2 exhibited some evidence. (b) A digestion-PCR-digestion (enriched template) of *Hen1a* and *Hen1b* amplicons from WPT589-2-1 and six T_1_ geminating seed template DNA. The template was enriched for mutated sequence by Hpy188I digestion of genomic DNA prior to PCR and amplicons were also digested post-PCR. Sequencing of these digest resistant amplicons revealed only wild-type sequence. (c) A Heteroduplex PCR assay using *Hen1a* and *Hen1b* specific primers on WPT589-1 and WPT589-2 T_0_ plants from multiple leaves (A-E) and six WPT589-2 T_1_ plants was carried out. The assay detected mutated sequence in the WPT589-1 T_0_ plants at both *Hen1a* and *Hen1b* loci however no mutants were detected in the WPT589-2 T_0_ or T_1_ plants. (d) Somatic mutations at the ‘Target1’ site were screened using enriched WPT589-2 T_1_ template and gel patterns suggesting mutated amplicons were observed, however these putative mutated amplicons could not been confirmed by sequencing.


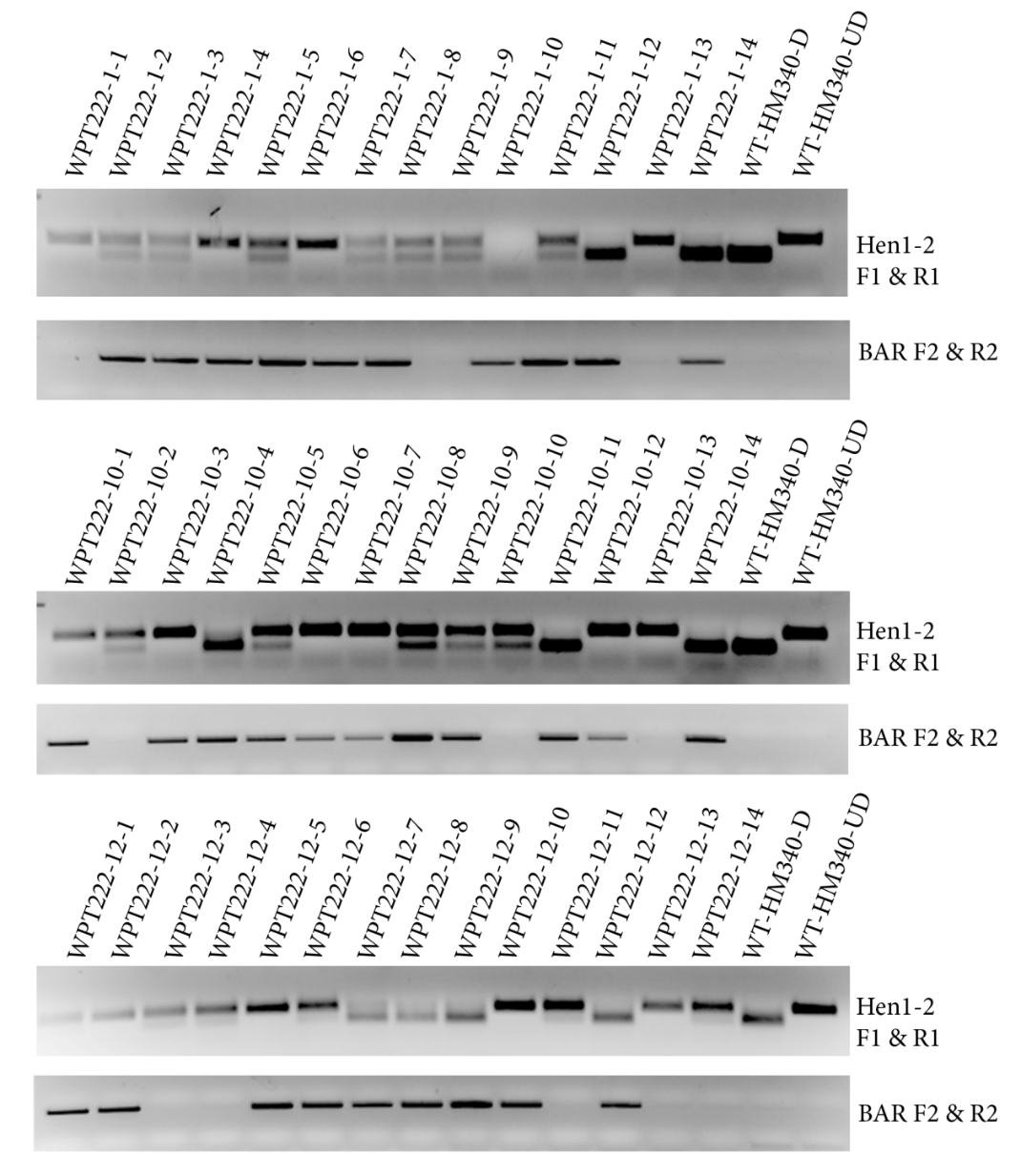


**Figure S20**. Screening for the segregation of mutations and transgenes in *M. truncatula* Hen1 T_1_ plants by PCR digestion assays. Undigested products (visualized as upper bands) indicate the presence of mutations relative to the wild-type allele. WT-HM340-D: Control where the PCR product from the wild-type digested genotype was digested. WT-HM340-UD: Control where the PCR product from the wild-type digested genotype was undigested.


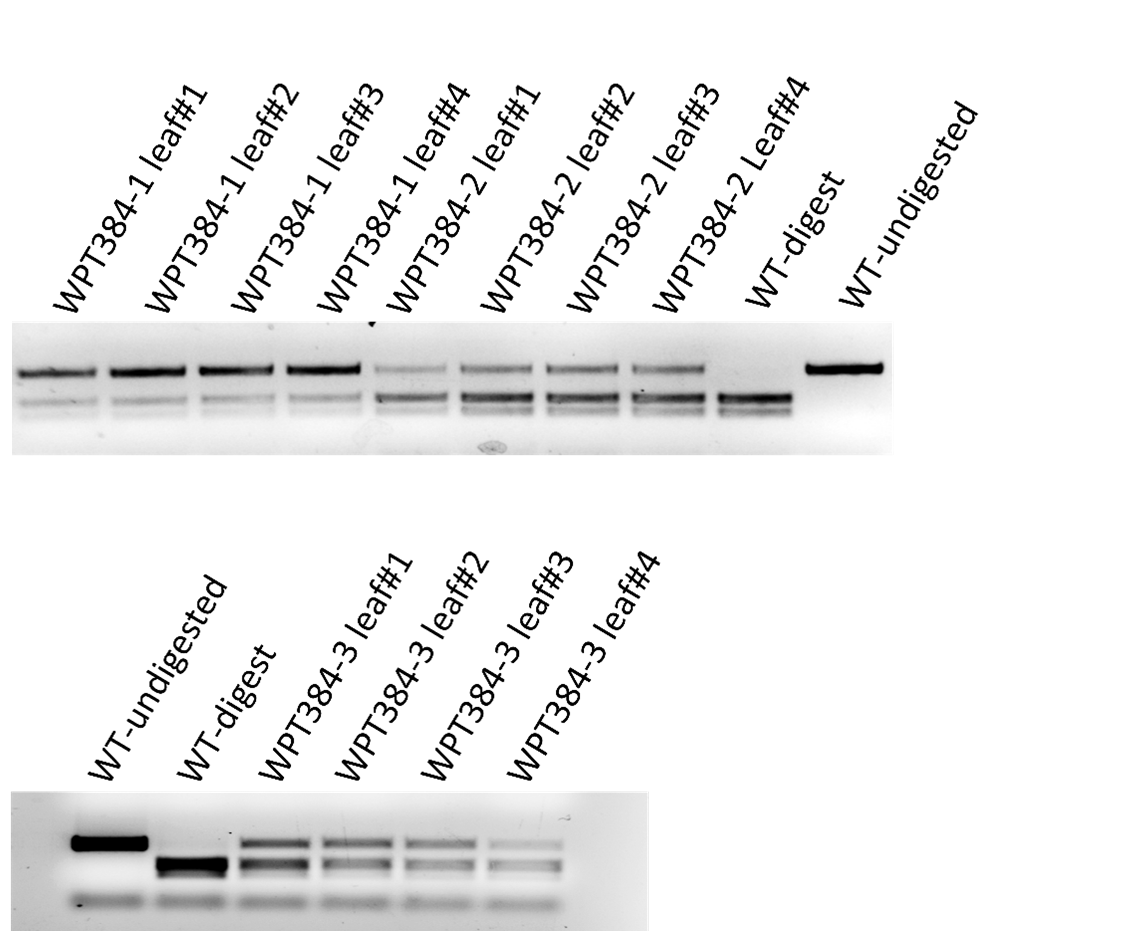
a


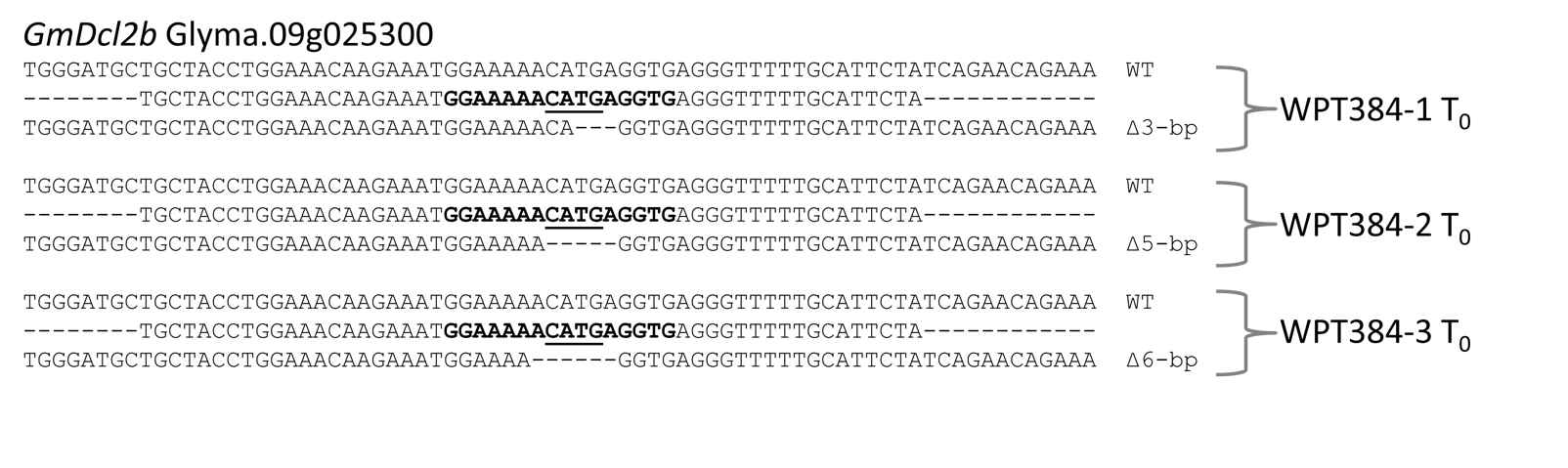
b

**Figure S21**. PCR-digestion assays of DNA template from four separate leaf samples from T_0_ plants transformed with a TALEN targeting *Dcl2b*. (a) Strong digestion resistant amplicons correlated with heritable transmission of the mutation (WPT384-1) compared to fainter amplicons that were not indicative of heritable transmission (WPT384-2 & WPT384-3) (b) Sequence confirmation of T_0_ mutations in WPT384-1, WPT384-2 and WPT384-3 plants. The bold sequence designates the spacer region between to two TAL binding array target sequences. The underlined sequences designate the restriction enzyme site of NlaIII used to screen mutants.


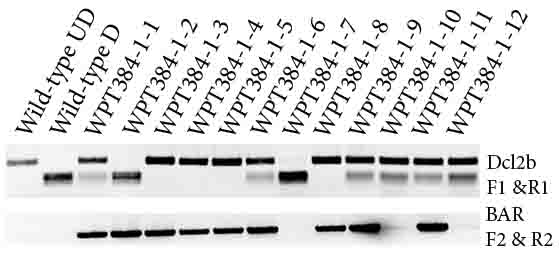


**Figure S22.** The heritable transmission of the *Dcl2b* mutation in WPT384-1 T_1_ plants and removal of the transgene by genetic segregation. The ‘Wild-type UD’ refers to undigested control amplicons and the ‘Wild-type D’ refers to digested wild-type amplicons. A PCR assay using primers to amplify the BAR gene was used to detect removal of the transgene by genetic segregation.


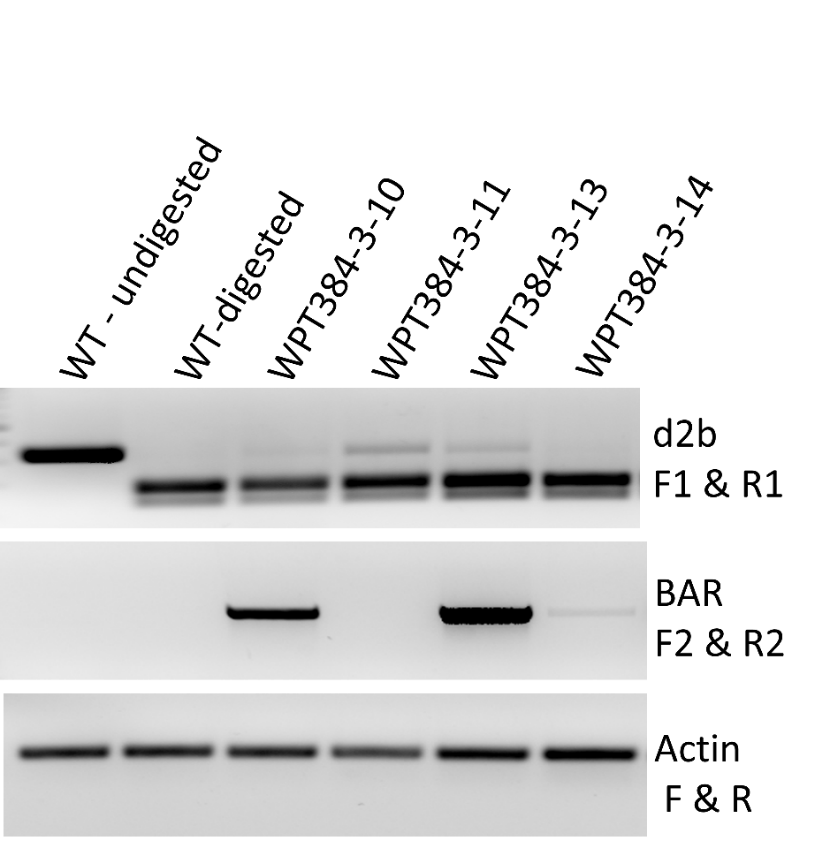
a

b

**
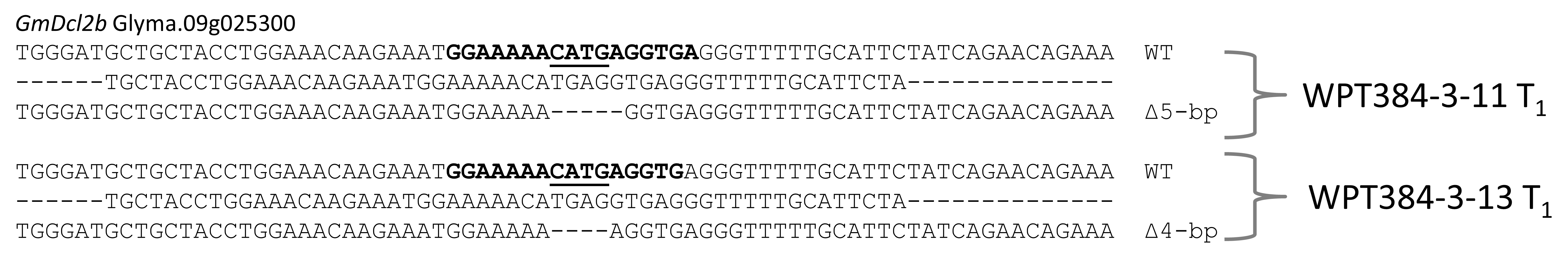
**

**Figure S23**. The heritable transmission of *Dcl2b* mutations was not observed in WPT384-2 and WPT384-3 T_0_ plants. (a) A PCR-digestion assay failed to detect germline heritable transmission in WPT384-2 and WPT384-3 plants. However, faint digestion resistant amplicons in T_1_ plants were observed suggesting the presence of somatic, non-germline mutations. The ‘WT undigested’ refers to undigested control amplicons and the ‘WT-digested’ refers to digested wild-type amplicons (b) Sequencing of these amplicons confirmed mutant sequences that likely resulted from an active reagent, though WPT384-3-11 appears to be a null-segregant. The bold sequence designates the spacer region between to two TAL binding array target sequences. The underlined sequences designates the restriction enzyme site of NlaIII used to screen mutants.


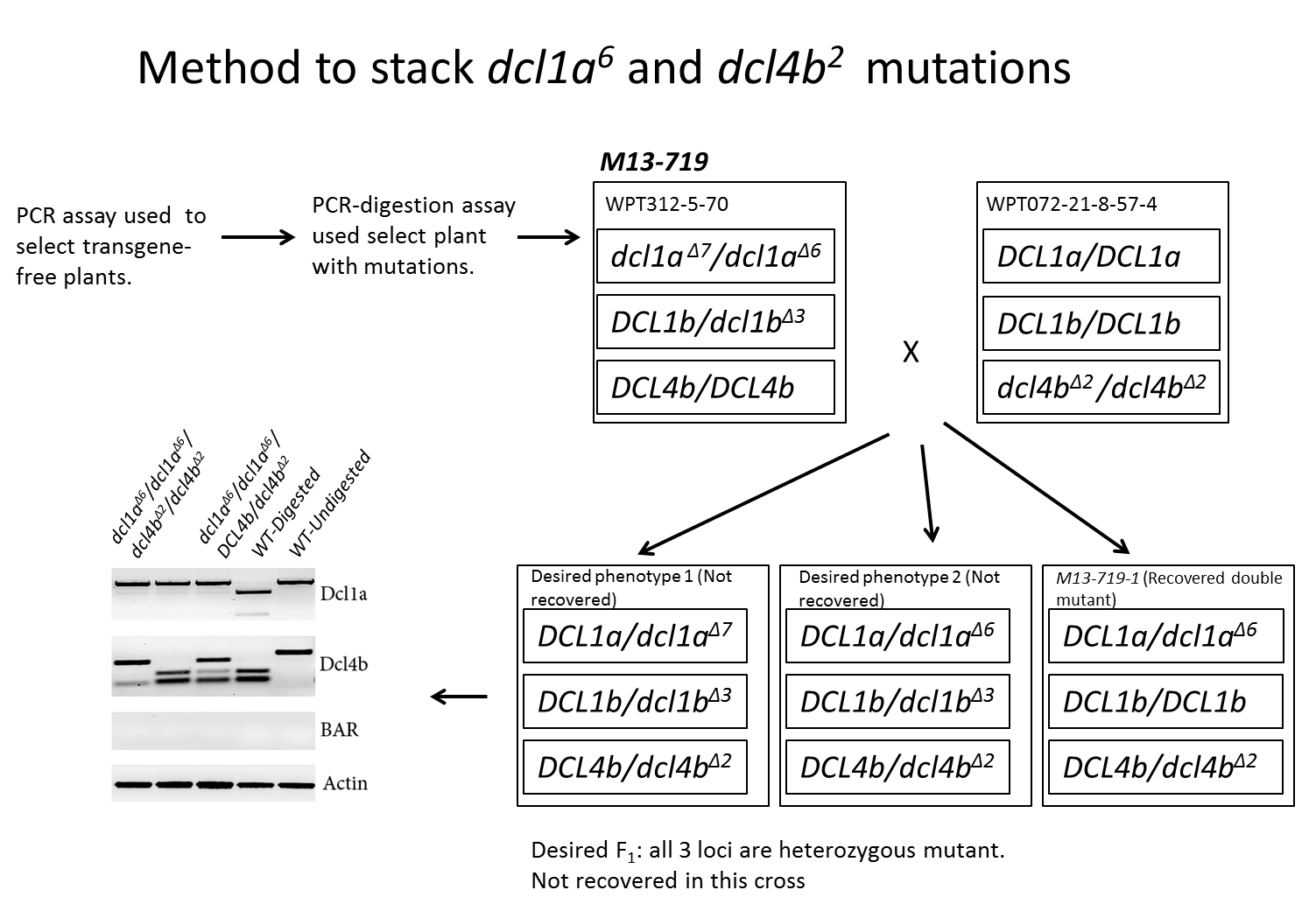


a

b

**Figure S24**. Combining *dcl1a* and *dcl4b* mutations. (a) The method used to stack the *dcl1a* and *dcl4b* mutations and (b) the PCR-digestion assay used to identify the double mutant (*dcl1a^Δ6^/dcl1a^Δ6^/dcl4b^Δ2^/dcl4b^Δ2^*) plant. The gel shows the progeny of M13-719-1, specifically a homozygous double mutant plant (first lane). The ‘WT undigested’ refers to undigested control amplicons and the ‘WT-digested’ refers to digested wild-type amplicons. PCR of the BAR gene failed to detect the presence of transgenic sequences.

**
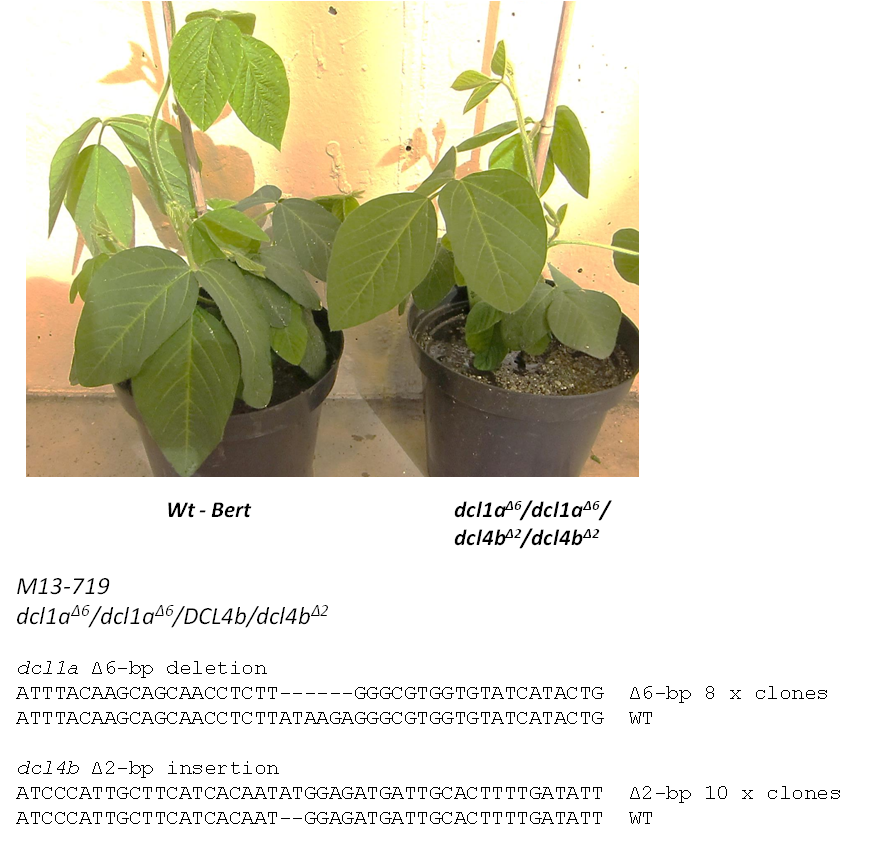
**a

b

**Figure S25.** The *dcl1a^Δ6^/dcl1a^Δ6^/dcl4b^Δ2^/dcl4b^Δ2^* and wild-type plant. (a) Three to four week old seedling plants of ‘Bert’ wild-type and the double mutant plant. (b) Target amplicons for *Dcl1a* and *Dcl4b* were cloned into pGem T easy and 8-10 clones sequenced. We observed the expected 6-bp deletion for *Dcl1a* and the 2-bp insertion for *Dcl4b* in all sequenced clones. No wild-type sequences were detected.


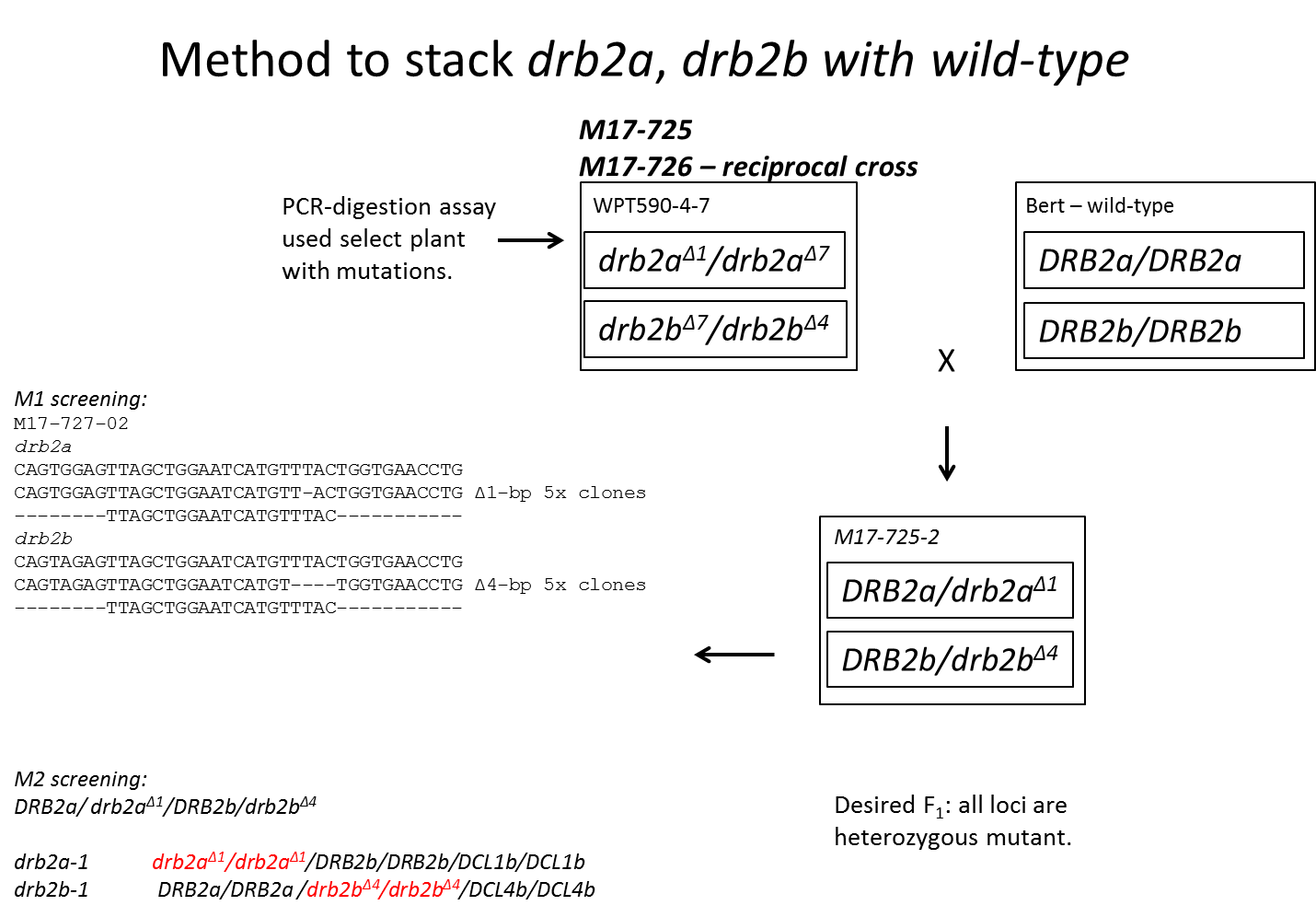


**Figure S26.** *Gmdrb2a^Δ7^/drb2a^Δ1^*/*drb2b^Δ7^/drb2b^Δ4^* was crossed to wild type to recover single *drb2a* and *drb2b* mutant plants. Initial M_1_ progeny were sequenced to confirm wild-type alleles. The red lettering indicates expected M_2_ genotype based on the mutant alleles observed in M_1_ plants.


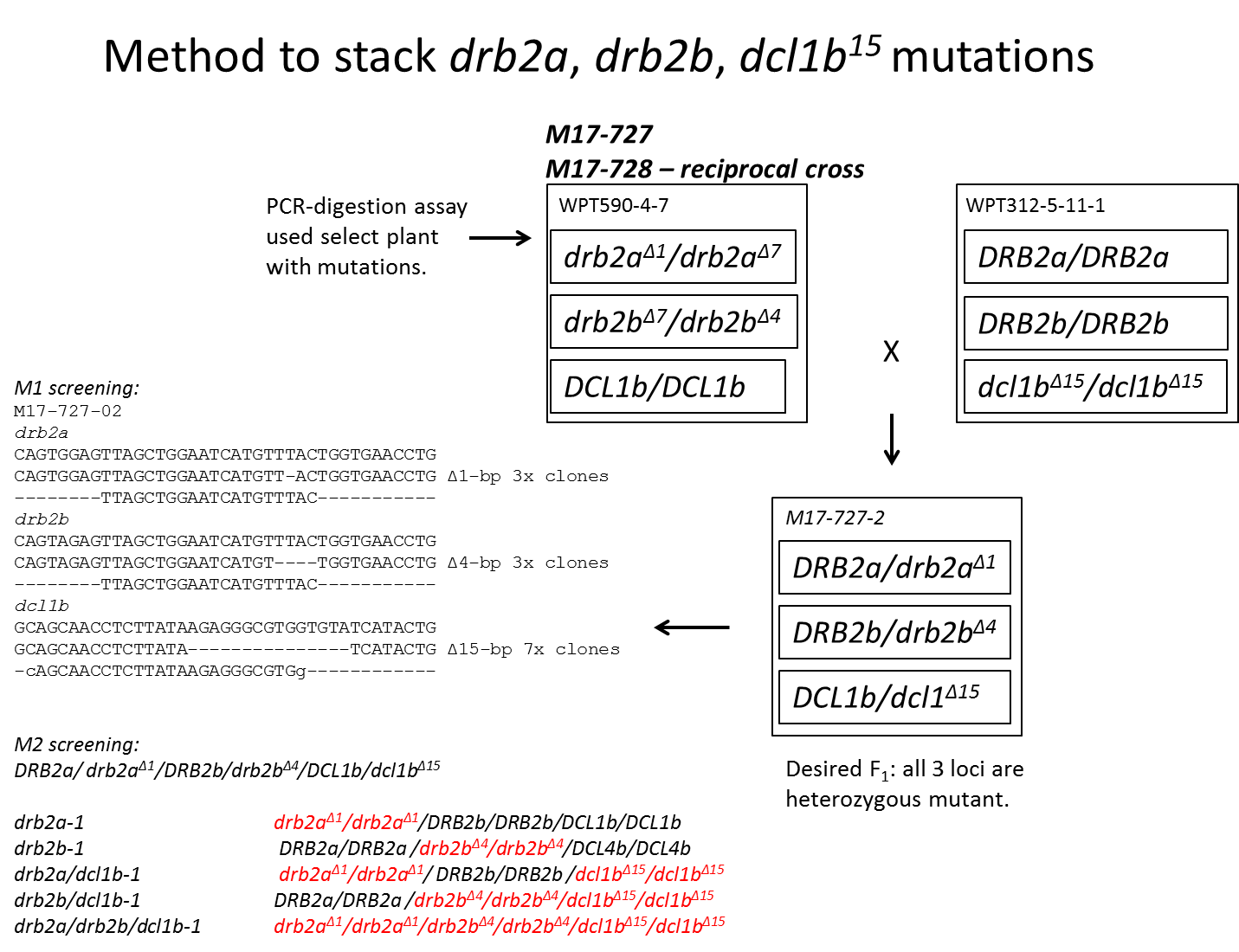


**Figure S27.** Combing the *Gmdrb2a^Δ7^/drb2a^Δ1^*/*drb2b^Δ7^/drb2b^Δ4^* and *Gmdcl1b^Δ15^* mutant alleles. The *drb2ab* double mutant was crossed to the single *dcl1b* mutant plant to recover a heterozygous triple mutant plants. Initial M_1_ progeny were sequenced to confirm the heterozygous *drb2a*, *drb2b* and *dcl1b* mutant alleles. The red lettering indicates expected M_2_ genotypes based on the mutant alleles observed in M_1_ plants.


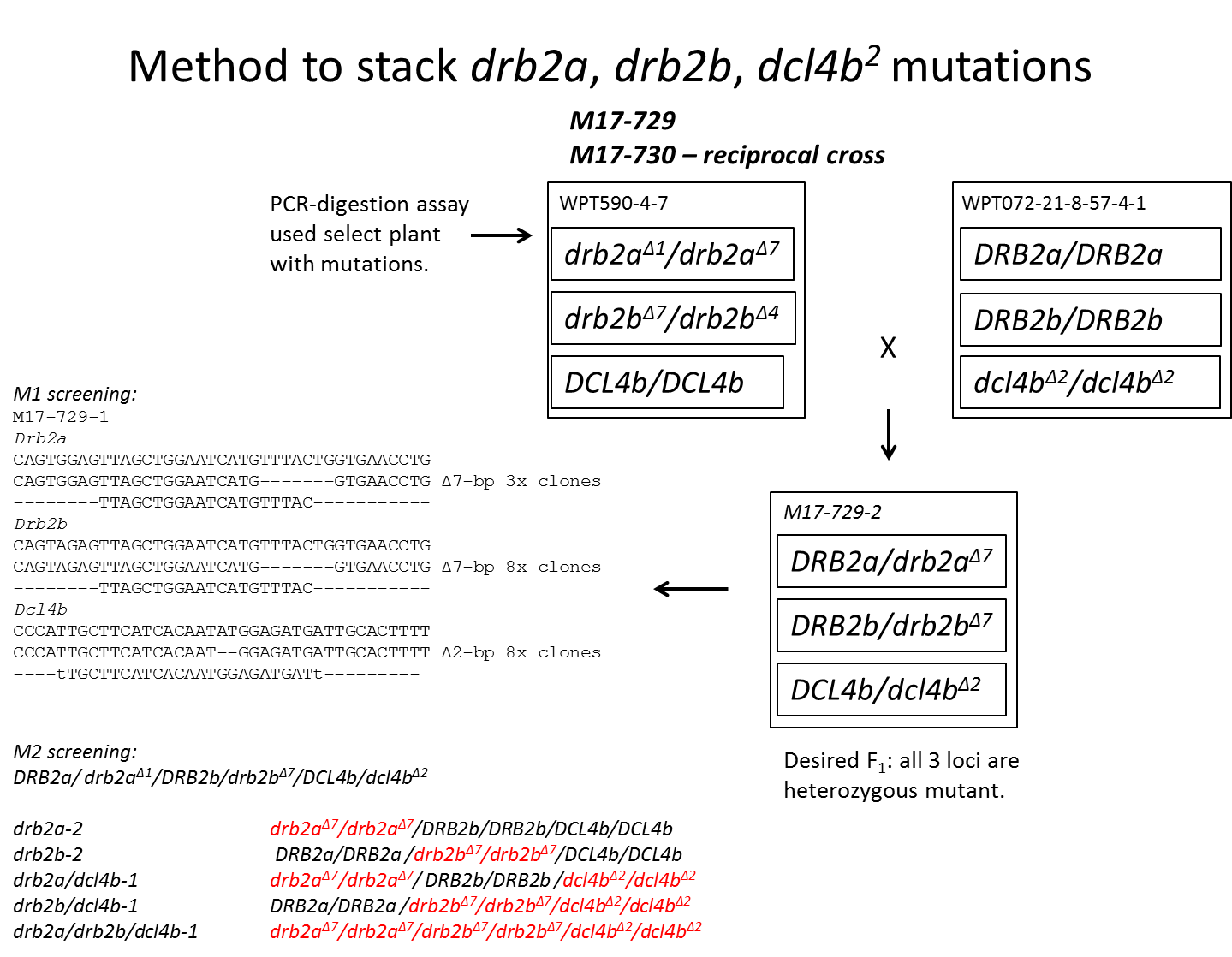


**Figure S28.** Combining the *Gmdrb2a^Δ7^/drb2a^Δ1^*/*drb2b^Δ7^/drb2b^Δ4^* and *Gmdcl4b^Δ2^* mutant alleles. The *drb2ab* double mutant was crossed to the single *dcl4b* mutant plant to recover a heterozygous triple mutant plants. Initial M_1_ progeny were sequenced to confirm the heterozygous *drb2a*, *drb2b* and *dcl4b* mutant alleles. The red lettering indicates expected M_2_ genotypes based on the mutant alleles observed in M_1_ plants.


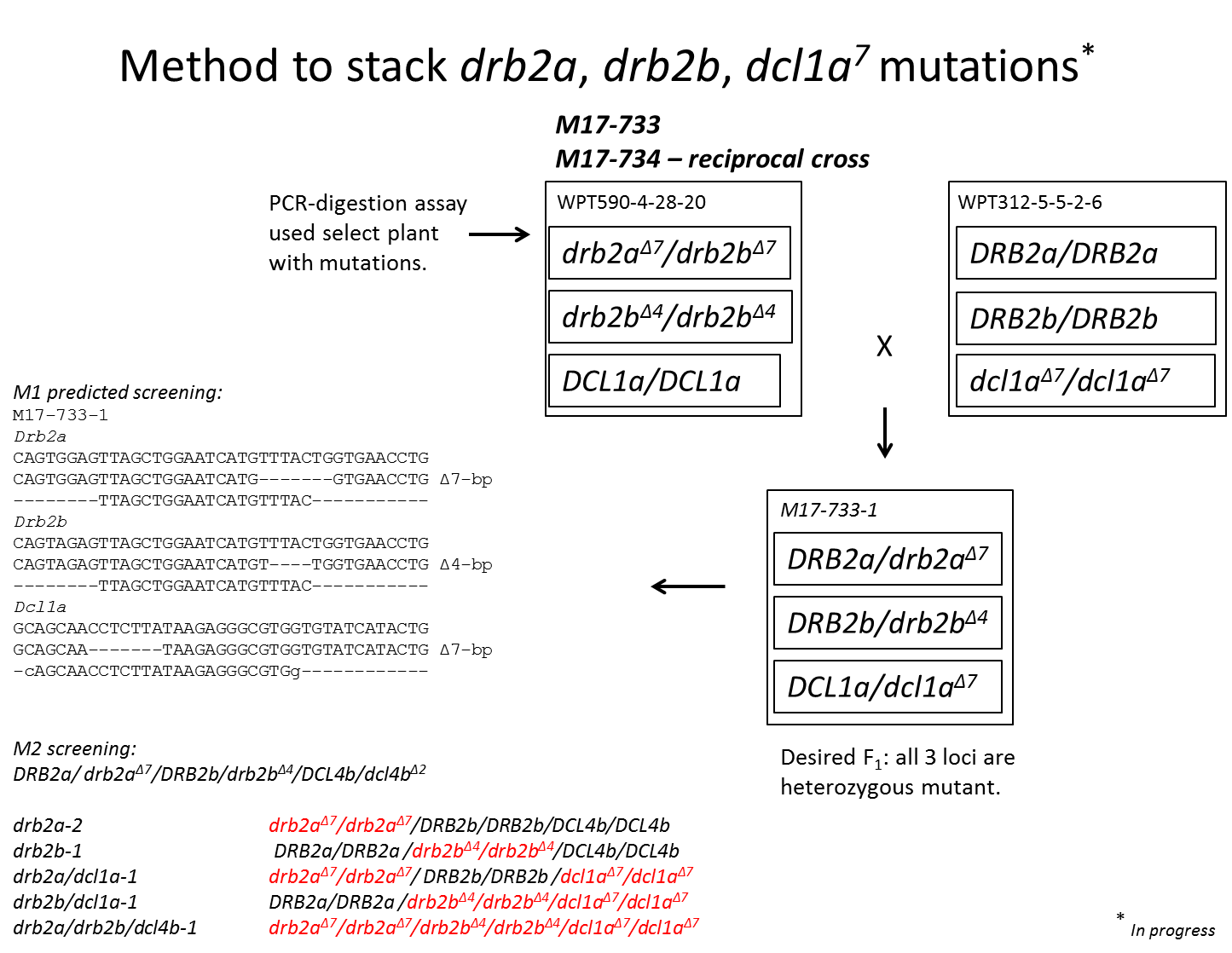


**Figure S29.** Combining the *Gmdrb2a^Δ7^/drb2a^Δ1^*/*drb2b^Δ7^/drb2b^Δ4^* and *Gmdcl1a^Δ7^* mutant alleles. The *drb2ab* double mutant was crossed to the single *dcl1a* mutant plant to recover a heterozygous triple mutant plants. Screening of the initial M_1_ progeny is currently in progress. The predicted mutant alleles are based on the known parent mutant alleles. The red lettering indicates expected M_2_ genotypes based on the mutant alleles observed in M_1_ plants.


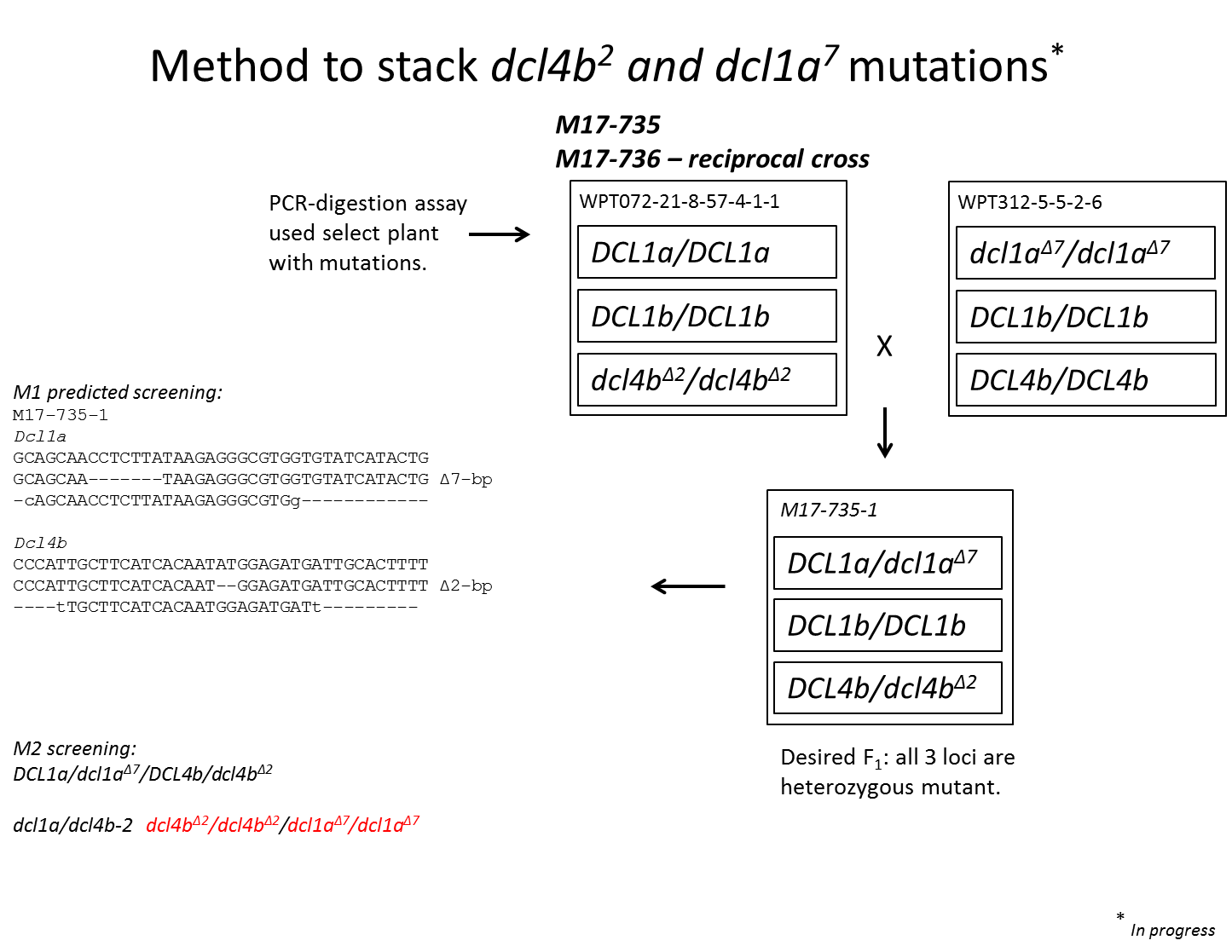


**Figure S30.** Combining the *Gmdcl4b^Δ2^* and *Gmdcl1a^Δ7^* mutant alleles. The *dcl1a* single frame-shift mutant was crossed to the single *dcl4b* mutant plant to recover a heterozygous double mutant plants. Screening of the initial M_1_ progeny is currently in progress. The predicted mutant alleles are based on the known parent mutant alleles. The red lettering indicates expected M_2_ genotypes based on the mutant alleles observed in M_1_ plants.

**
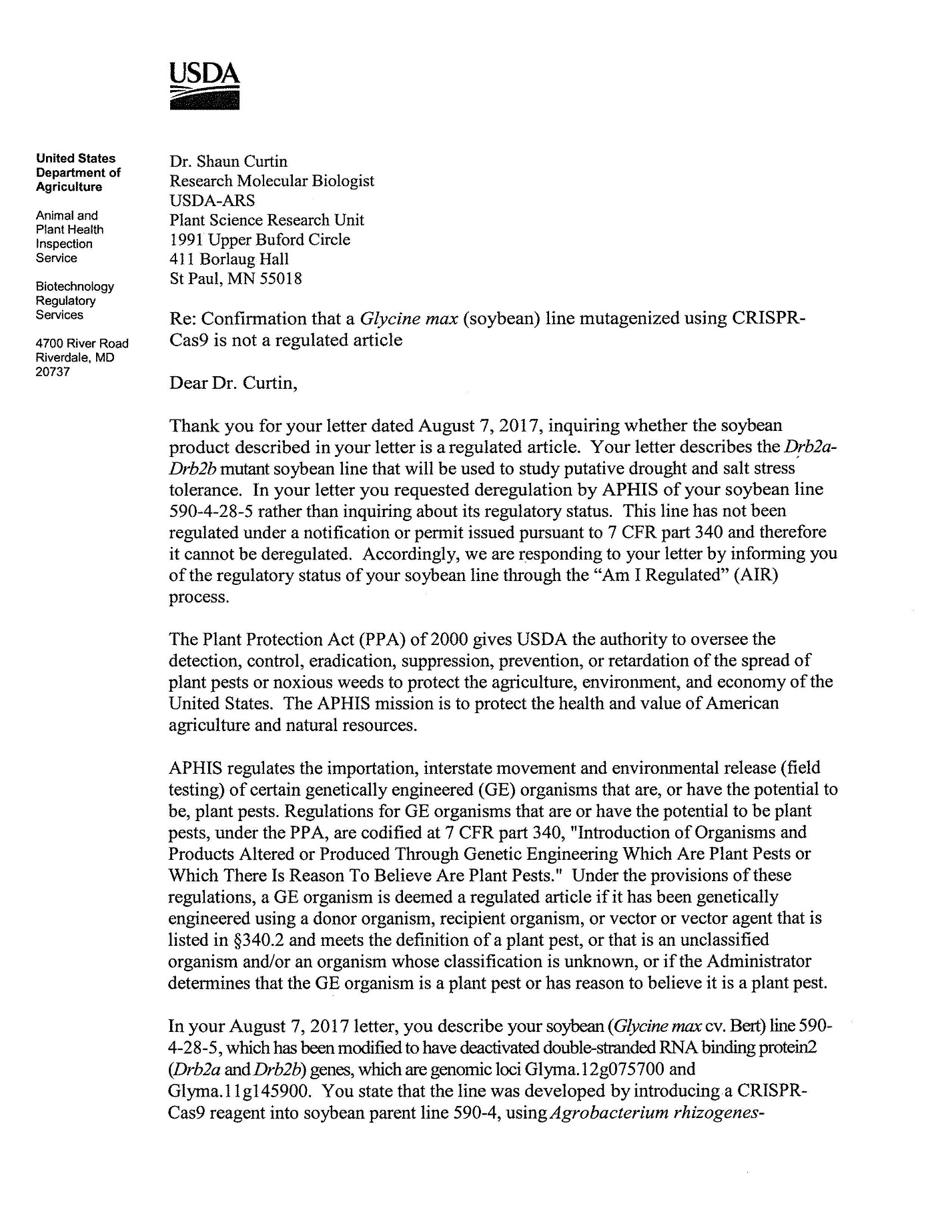
**


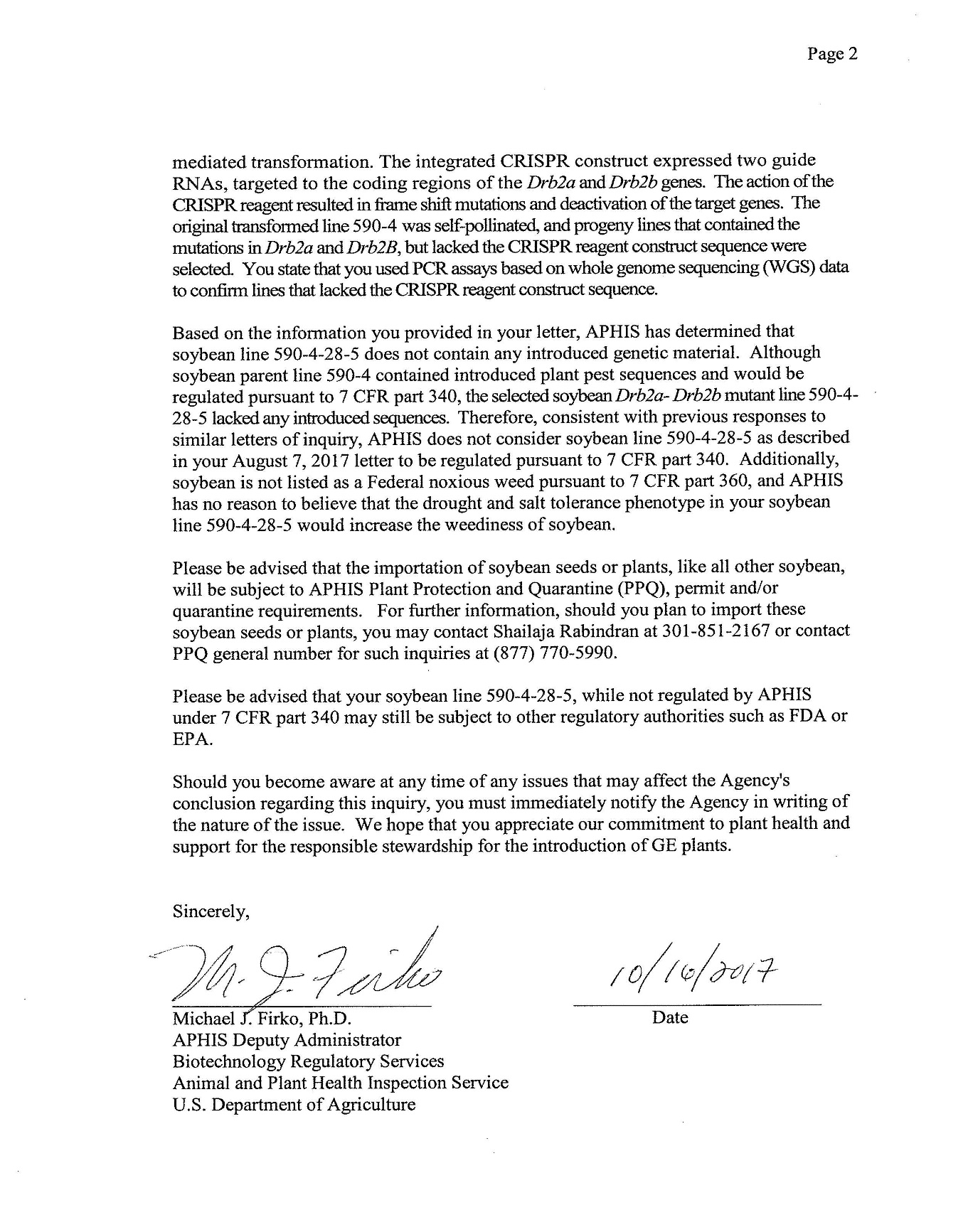


**Figure S31.** USDA-APHIS confirmation that *Glycine max* (soybean) line WPT590-4-28-5 is not a regulated article


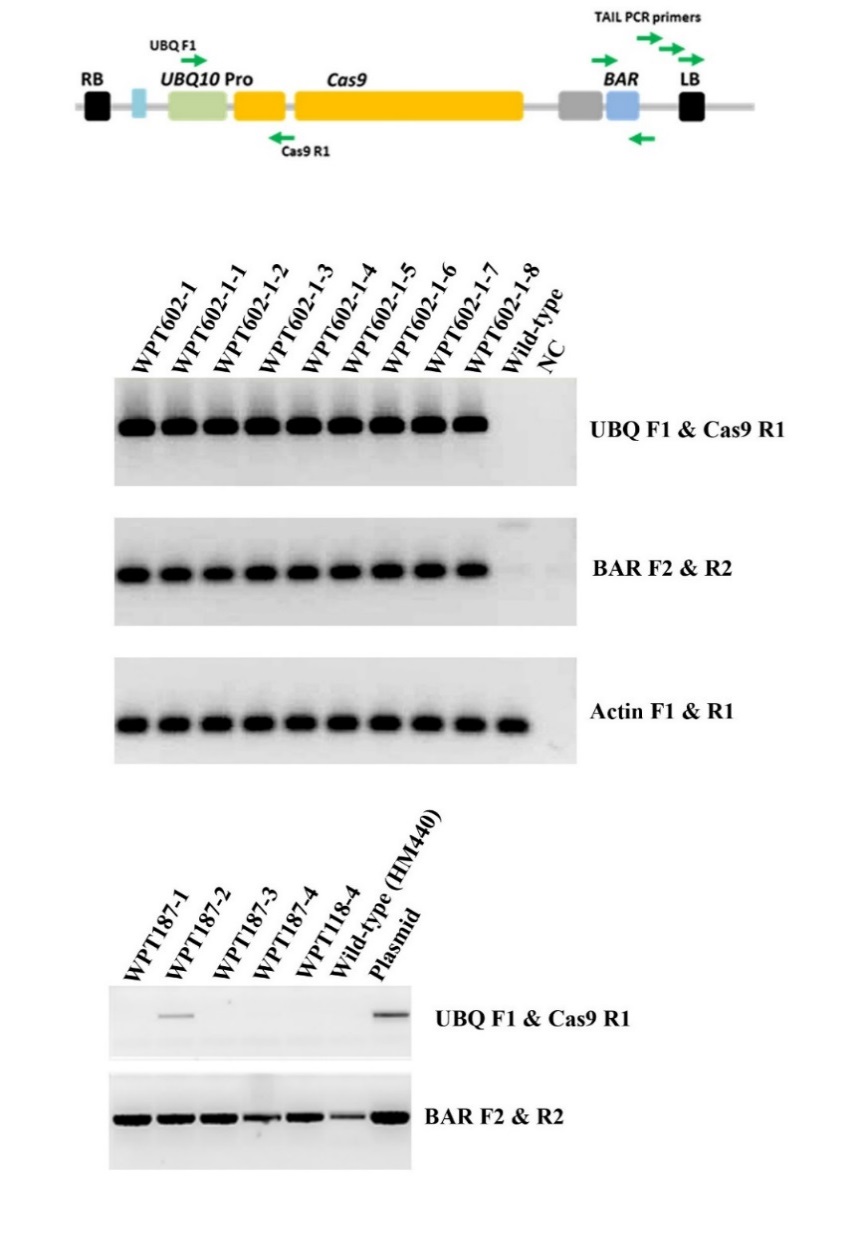


a

b

c

**Figure S32.** The screening of Cas9 over-expression cassette in whole soybean and *M. truncatula* plants. (a) The AtUBQ10 Cas9 construct was transformed into both soybean and *M. truncatula.* Green arrows indicate primers used for analysis. (b) The screening of soybean WPT602 T_0_ and T_1_ plants for the presence the Cas9 transgene. NC indicates negative PCR control (c) The screening of *M. truncatula* WPT187 T_0_ plants for the presence the Cas9 transgene. The T_0_ WPT187-2 was positive for Cas9 and a TAIL PCR assay was carried out to identify its genomic location in *M. truncatula*. (see See Supplemental Table 5 for primers).

**Supplemental Method1.**

**TALEN design and assembly**

Nine target sites, three for each gene, were identified using the publicly available TAL effector Nulceotide Targeter 2.0. (TALE-NT). TAL arrays were first cloned into yeast expression vectors pTAL500 and pTAL501 by a gold-gate cloning assay (Doyle *et al.*, 2012; Cermak *et al.*, 2011). Nuclease activity of all nine TAL arrays were tested using an in *vivo* yeast assay (Townsend *et al.*, 2009). The yeast assay identified three candidate TAL array pairs that exhibited significant cleavage activity for targets *Dcl2a*^240/241^, *Dcl2b*^602/603^ and *Dcl3a*^186/187^ (Figure S3b). Each of the TAL arrays ranged in length from 15 – 21 RVD units with the spacer region ranging from 15-21-bp. For delivery into soybean, the TAL arrays were cloned into the Gateway™-compatible entry vector pZHY013 with the N152/C63 TALEN backbone architecture, so-called for its truncated TAL-effector N-terminus and C-terminus (152 aa and 63 aa) respectively (Zhang *et al.*, 2013). This variant architecture has been shown to improve nuclease activity compared to the wild-type effector protein (Miller *et al.*, 2011). It also houses the EL/KK FokI heterodimers and the viral T2a ribosome skipping peptide for the translation of both left and right TALENs from a single transcript (Zhang *et al.*, 2013).

**Supplemental Method2.**

**Soybean genetic hybridization assay**

The soybean *dcl1a* and *dcl4b* mutants were cross hybridized to combine mutant alleles in the same plant. The crossings were performed in a thoroughly cleaned and vacuumed growth chamber. All walls, grates and backs of doors were spray wiped with bleach. Before planting, the chamber was heated to 45^o^C overnight to remove potential insect contamination and all lamps were re-lamped with new lights. Seeds were germinated in large pots of half-soil/half- metromix and a slow-release fertilizer. Lighting conditions were adjusted to 16 hours light, 8 eight hours dark at 21^o^C and high humidity. The lights were kept at approximately 20mm above the tops of the plants at all times to increase light intensity. Three weeks after germination, the daylight length was reduced by 30 minutes each week until a length 14.30 hours. The light was held at this day length until the completion of all crossings and the pods were approximately 35mm in length. Next, the day length was dropped 20 minutes every four days until 13:00 hours. At this time, the chamber temperature was raised to 27-29^o^C to hasten maturity.

**Supplemental Method3.**

**Heteroduplex assay for detection of targeted mutations**

Following PCR completion, the reaction was incubated for 5 minutes at 95^o^C and for 10 minutes at room temperature. 2.5uL of Novex 5X Hi-Density Sample Buffer (Life Technologies, CA) was added to 10uL of the heteroduplex reaction. The reactions were loaded on a Novex 4-20% TBE non-denaturing polyacrylamide gel electrophoresis (PAGE) (Life Technologies, CA) and run in 1X TBE at 200V for 60 minutes using the Xcell Sure Lock Mini-Cell system (Life Technologies, CA). The gel was stained for 15 minutes with 2.5ul ethidium bromide (10mg/mL) in 100mL deionized water followed by a 15 minute de-stain in deionized water. The gel was visualized using the Bio-rad GelDoc system (Jacobs *et al.*, 2015; Zhu *et al.*, 2014).

**Supplemental Method4.**

**Identifying transgene junctions and CRISPR deletions**

DNA samples were sent to the University of Minnesota’s genomic center for sequencing using an Illumina HiSeq2500 with v4 chemistry to generate 125bp parried-end reads. Reads were checked for initial quality using Fastqc version 0.11.5 and Illumina Truseq adapters were trimmed using cutadapt version 1.8.1 with a minimum read length set to 40bp and quality cutoff set to a phread score of 30 (Andrews, 2010; Martin, 2011). To map reads to the soybean reference genome (Wm82.a2.v1), bwa version 0.7.12 was used with band width set to 100, mark shorter splits as secondary, penalty for mismatch set to 6 (Li and Durbin, 2009). Samtools version 1.3 was used to convert any SAM file format to BAM format, sort, and index files (Li *et al.*, 2009). Identification of transgene insertion sites was performed in a manner similar to (Srivastava *et al.*, 2014). Fasta files were created using the transgene cassette with 100 bp flanking backbone sequence to serve as our reference genome. Sequenced reads were then mapped to transgene reference using the same programs and parameters used to map reads to the reference genome. Orphaned reads were extracted using a modified version of extract_unmapped_mates.pl to accept bam files as input. Orphaned reads we then mapped to the Wm82.a2.v1 reference using bowtie2 version 2.2.4 using -- local -- very-sensitive-local (Langmead *et al.*, 2009). SAM files were then converted to BAM file format, sorted and indexed in the same manner mentioned above. Orphaned reads that mapped to the reference were further investigated upon using IGV version 2.3.90 (Robinson *et al.*, 2011). Orphaned read mapping was then compared to read mapping to the soybean reference and the parental line (BertMN01) as a control. Deletions were investigated using IGV at each CRISPR site throughout the genome.

Scripts used in this study have been archived at the following github site;

<https://github.com/MeeshCompBio/Soybean_Scripts>

**Supplemental Method5.**

**Phenotypic characterization of mutants**

Preliminary analysis of drought tolerance was conducted on the WPT590-4-28 T_1_ progeny derived from the *Gmdrb2a/b* double-mutant. The wild-type control, Bert was used as this is the cultivar in which the WPT590 transformation was performed. Twenty-four mutant and wild-type plants were grown in a growth chamber in pots filled with metro-mix and irrigated daily. Fourteen days after germination irrigation was withheld and drought tolerance monitored for a 7-day period. Following the stress treatment, drought tolerance was scored using a 1 to 5 scale (1 - drought tolerant to 5 - drought sensitive). Stem-loop qPCR quantification of miRNA abundance was performed according to Reis *et al.*, (2016), to assess the level of specific miRNAs in mutant and wild-type tissues. Total RNA was collected from leaflets and apical shoot tips of mutant and wild-type plants fourteen days after germination. SuperScript® IV Reverse Transcriptase (Life Technologies) and SYBR Green PCR Master Mix (Applied Biosystem) were used to perform cDNA synthesis and RT-qPCR, respectively. Soybean U6 was used to normalize miRNA abundance (Turner *et al.*, 2013)

For SEM analysis, leaf pieces were cut into 5mm^2^ pieces and fixed overnight in 2% glutaraldehyde in 0.1M sodium cacodylate buffer under vacuum. Samples were rinsed in 0.1M sodium cacodylate buffer, post-fixed in 1% osmium tetroxide in 0.1M sodium cacodylate buffer for 4h, and rinsed in ultrapure water (NANOpure Infinity®; Barnstead/Thermo Fisher Scientific; Waltham, Maryland). Leaf pieces were then dehydrated in an ethanol series (25%, 50%, 75%, 95% (2x) and 100% (3x); 10 min for each change) and dried in a critical point dryer (Autosamdri-814; Tousimis; Rockville, Maryland). Material was mounted on aluminum stubs using double-sided adhesive carbon tabs and carbon paint, sputter-coated with gold-palladium, and observed in a scanning electron microscope (S3500N; Hitachi High Technologies America, Inc.; Schaumberg, Illinois) at an accelerating voltage of 10 kV.

**References**

**Andrews, S.** (2010) FastQC: A quality control tool for high throughput sequence data. *http://www.bioinformatics.babraham.ac.uk/projects/fastqc/*, http://www.bioinformatics.babraham.ac.uk/projects/.

**Cermak, T., Doyle, E.L., Christian, M., et al.** (2011) Efficient design and assembly of custom TALEN and other TAL effector-based constructs for DNA targeting. *Nucleic Acids Res.*, **39**, e82.

**Christian, M., Cermak, T., Doyle, E.L., Schmidt, C., Zhang, F., Hummel, A., Bogdanove, A.J. and Voytas, D.F.** (2010) Targeting DNA double-strand breaks with TAL effector nucleases. *Genetics*, **186**, 757–61.

**Doyle, E.L., Booher, N.J., Standage, D.S., Voytas, D.F., Brendel, V.P., Vandyk, J.K. and Bogdanove, A.J.** (2012) TAL Effector-Nucleotide Targeter (TALE-NT) 2.0: Tools for TAL effector design and target prediction. *Nucleic Acids Res.*, **40**.

**Jacobs, T., LaFayette, P., Schmitz, R. and Parrott, W.** (2015) Targeted genome modifications in soybean with CRISPR/Cas9. *BMC Biotechnol.*, **15**, 1–10.

**Langmead, B., Trapnell, C., Pop, M. and Salzberg, S.L.** (2009) Ultrafast and memory-efficient alignment of short DNA sequences to the human genome. *Genome Biol.*, **10**, R25.

**Li, H. and Durbin, R.** (2009) Fast and accurate short read alignment with Burrows-Wheeler transform. *Bioinformatics*, **25**, 1754–1760.

**Li, H., Handsaker, B., Wysoker, A., Fennell, T., Ruan, J., Homer, N., Marth, G., Abecasis, G. and Durbin, R.** (2009) The Sequence Alignment/Map format and SAMtools. *Bioinformatics*, **25**, 2078–9.

**Martin, M.** (2011) Cutadapt removes adapter sequences from high-throughput sequencing reads. *EMBnet.journal*, **17**, 10.

**Miller, J.C., Tan, S., Qiao, G., et al.** (2011) A TALE nuclease architecture for efficient genome editing. *Nat. Biotechnol.*, **29**, 143–8.

**Robinson, J.T., Thorvaldsdóttir, H., Winckler, W., Guttman, M., Lander, E.S., Getz, G. and Mesirov, J.P.** (2011) Integrative genomics viewer. *Nat. Biotechnol.*, **29**, 24–26.

**Srivastava, A., Philip, V.M., Greenstein, I., Rowe, L.B., Barter, M., Lutz, C. and Reinholdt, L.G.** (2014) Discovery of transgene insertion sites by high throughput sequencing of mate pair libraries. *BMC Genomics*, **15**, 367.

**Townsend, J.A., Wright, D.A., Winfrey, R.J., Fu, F., Maeder, M.L., Joung, J.K. and Voytas, D.F.** (2009) High-frequency modification of plant genes using engineered zinc-finger nucleases. *Nature*, **459**, 442–5.

**Zhang, Y., Zhang, F., Li, X., Baller, J. a, Qi, Y., Starker, C.G., Bogdanove, A.J. and Voytas, D.F.** (2013) Transcription activator-like effector nucleases enable efficient plant genome engineering. *Plant Physiol.*, **161**, 20–7.

**Zhu, X., Xu, Y., Yu, S., et al.** (2014) An efficient genotyping method for genome-modified animals and human cells generated with CRISPR/Cas9 system. *Sci Rep*, **4**, 6420.
